# Blue Biotechnology: Computational Screening of *Sarcophyton* Cembranoid Diterpenes for SARS-CoV-2 Main Protease Inhibition

**DOI:** 10.3390/md19070391

**Published:** 2021-07-13

**Authors:** Mahmoud A. A. Ibrahim, Alaa H. M. Abdelrahman, Mohamed A. M. Atia, Tarik A. Mohamed, Mahmoud F. Moustafa, Abdulrahim R. Hakami, Shaden A. M. Khalifa, Fahad A. Alhumaydhi, Faris Alrumaihi, Syed Hani Abidi, Khaled S. Allemailem, Thomas Efferth, Mahmoud E. Soliman, Paul W. Paré, Hesham R. El-Seedi, Mohamed-Elamir F. Hegazy

**Affiliations:** 1 Computational Chemistry Laboratory, Chemistry Department, Faculty of Science, Minia University, Minia 61519, Egypt; a.abdelrahman@compchem.net; 2 Molecular Genetics and Genome Mapping Laboratory, Genome Mapping Department, Agricultural Genetic Engineering Research Institute (AGERI), Agricultural Research Center (ARC), Giza 12619, Egypt; matia@ageri.sci.eg; 3 Chemistry of Medicinal Plants Department, National Research Centre, 33 El-Bohouth St., Dokki, Giza 12622, Egypt; tarik.nrc83@yahoo.com; 4 Department of Biology, College of Science, King Khalid University, Abha 9004, Saudi Arabia; hamdony@yahoo.com or; 5 Department of Botany & Microbiology, Faculty of Science, South Valley University, Qena 83523, Egypt; 6 Department of Clinical Laboratory Sciences, College of Applied Medical Sciences, King Khalid University, Abha 61481, Saudi Arabia; ahakami@kku.edu.sa; 7 Department of Molecular Biosciences, The Wenner-Gren Institute, Stockholm University, S-106 91 Stockholm, Sweden; shaden.khalifa@su.se; 8 Department of Medical Laboratories, College of Applied Medical Sciences, Qassim University, Buraydah 51452, Saudi Arabia; f.alhumaydhi@qu.edu.sa (F.A.A.); f_alrumaihi@qu.edu.sa (F.A.); k.allemailem@qu.edu.sa (K.S.A.); 9 Department of Biological and Biomedical Sciences, Aga Khan University, Karachi 74800, Pakistan; m.haniabidi@gmail.com; 10 Department of Pharmaceutical Biology, Institute of Pharmaceutical and Biomedical Sciences, Johannes Gutenberg University, Staudinger Weg 5, 55128 Mainz, Germany; efferth@uni-mainz.de; 11 Molecular Modelling and Drug Design Research Group, School of Health Sciences, University of KwaZulu-Natal, Westville, Durban 4000, South Africa; soliman@ukzn.ac.za; 12 Department of Chemistry & Biochemistry, Texas Tech University, Lubbock, TX 79409, USA; paul.pare@ttu.edu; 13 Department of Chemistry, Faculty of Science, El-Menoufia University, Shebin El-Kom 32512, Egypt; 14 International Research Center for Food Nutrition and Safety, Jiangsu University, Zhenjiang 212013, China; 15 Pharmacognosy Group, Department of Pharmaceutical Biosciences, Uppsala University, Biomedical Centre, Box 574, 751 23 Uppsala, Sweden

**Keywords:** genus *Sarcophyton*, cembranoid diterpenes metabolites, SARS-CoV-2 main protease, molecular docking, molecular dynamics, reactome

## Abstract

The coronavirus pandemic has affected more than 150 million people, while over 3.25 million people have died from the coronavirus disease 2019 (COVID-19). As there are no established therapies for COVID-19 treatment, drugs that inhibit viral replication are a promising target; specifically, the main protease (M^pro^) that process CoV-encoded polyproteins serves as an Achilles heel for assembly of replication-transcription machinery as well as down-stream viral replication. In the search for potential antiviral drugs that target M^pro^, a series of cembranoid diterpenes from the biologically active soft-coral genus *Sarcophyton* have been examined as SARS-CoV-2 M^pro^ inhibitors. Over 360 metabolites from the genus were screened using molecular docking calculations. Promising diterpenes were further characterized by molecular dynamics (MD) simulations based on molecular mechanics-generalized Born surface area (MM-GBSA) binding energy calculations. According to in silico calculations, five cembranoid diterpenes manifested adequate binding affinities as M^pro^ inhibitors with Δ*G*_binding_ < −33.0 kcal/mol. Binding energy and structural analyses of the most potent *Sarcophyton* inhibitor, bislatumlide A (**340**), was compared to darunavir, an HIV protease inhibitor that has been recently subjected to clinical-trial as an anti-COVID-19 drug. In silico analysis indicates that **340** has a higher binding affinity against M^pro^ than darunavir with Δ*G*_binding_ values of −43.8 and −34.8 kcal/mol, respectively throughout 100 ns MD simulations. Drug-likeness calculations revealed robust bioavailability and protein-protein interactions were identified for **340**; biochemical signaling genes included ACE, MAPK14 and ESR1 as identified based on a STRING database. Pathway enrichment analysis combined with reactome mining revealed that **340** has the capability to re-modulate the p38 MAPK pathway hijacked by SARS-CoV-2 and antagonize injurious effects. These findings justify further in vivo and in vitro testing of **340** as an antiviral agent against SARS-CoV-2.

## 1. Introduction

Coronavirus (CoV) belonging to the Coronaviridae family is one of the largest families of positive-sense, RNA viruses [1]. Based on genomic structure and phylogenetics, CoVs are subdivided into genera alpha through delta with the alpha- and beta-genera responsible for currently known diseases in humans [1]. Unlike subtypes of CoVs that cause mild clinical symptoms, SARS-CoV and MERS-CoV are associated with severe respiratory symptoms [2]. CoVs are recognized to cause infection in the respiratory, hepatic, enteric and neurological systems and pandemic conditions have ensued with high infection rates [3]. Since the World Health Organization (WHO) announced this new beta coronavirus in late 2019, cataloged as SARS-CoV-2 (COVID-19) [4], the organization has authorized several COVID-19 preparations for emergency use immunization including vaccines developed by Pfizer/BioNTech, Astrazeneca/Oxford, Serum Institute of India, Johnson & Johnson, Moderna and Sinopharm. Notwithstanding these advances in controlling the pandemic, with high viral transmission via respiratory droplets from coughing and/or sneezing [5], there is still an urgency to developing effective antiviral drugs for COVID-19 treatment.

In screening for potential COVID-19 drug candidates, metabolite repurposing can be used as a starting point to identify metabolites that already have biological activity against some diseases or infections. Drug repurposing can be achieved by conducting systematic drug-drug target interaction and drug-drug interaction analyses [6]. Another approach is to examine a class or source of natural products with established biological activity; indeed, several in silico studies have been developed to screen for SARS-CoV-2 inhibitors as prospective anti-COVID-19 drug candidates [7,8,9,10,11,12,13]. Natural products play a pivotal role in designing novel and efficient treatments to conquer the current COVID-19 epidemic. Among natural products, alkaloids, flavonoids and terpenoids have enticed considerable attention as potential SARS-CoV-2 drug candidates [14,15,16].

The basis of such studies is that the main protease (M^pro^) that cleaves COVID-19 polyproteins is a point of vulnerability for viral replication. The crystal structure of SARS-CoV-2 main protease provides the basis for designing small molecules as inhibitors [17]. As a result, inhibitors can be screened for a suitable molecular structure to bind to the catalytic site of M^pro^ and interrupt viral replication. Here, we examine cembranoids, a class of diterpenes isolated predominantly from the soft coral genus *Sarcophyton* that contain a considerable reservoir of bioactive natural products [18]. While screening for marine natural products with pharmacological activity of has led to the discovery of many potent bioactive metabolites [19], the marine pharmacopeia is still a rich source of biological and chemical diversity [20,21,22,23].

In this *Sarcophyton* specific study, metabolites identified over the last two decades (1998–2019) [18] provided the original database for creating a *Sarcophyton* chemical library; a subset of those compounds were then virtual screened as M^pro^ specific inhibitors. According to the anticipated docking scores, the most potent compounds were subjected to molecular dynamics (MD) simulations combined with binding energy calculations utilizing the molecular mechanics-generalized Born surface area (MM-GBSA) approach. The screen identified a promising anti-SARS-CoV-2 agent. Such in silico screening can provide promising inhibitor leads for subsequent in vitro and in vivo studies.

## 2. Results

The unavailability of approved therapies towards COVID-19 disease indicates a crucial demand to systematically screen and recognize inhibitors that can suppress the SARS-CoV-2 infection and/or replication. M^pro^ has an indispensable role in the viral reproduction in the host and is considered one of the most promising drug targets. In seeking small molecules to prohibit SARS-CoV-2 replication, molecular docking calculations and molecular dynamics (MD) simulations were applied to scrutinize the potency of a chemical library containing 363 diterpenes as prospective anti-COVID-19 inhibitor candidates.

### 2.1. Molecular Docking

The molecular docking technique was employed to anticipate the binding affinities for diterpenes towards M^pro^. The predicted docking scores of all investigated diterpenes metabolites are tabulated in Table S1. What stands out in Table S1 is the wide range of binding affinities ranging from −4.3 to −10.4 kcal/mol. To evaluate the potency of the studied metabolites, docking scores were compared to darunavir, an antiretroviral therapy that has been clinically tested as a COVID-19 drug candidate [24]. Although darunavir has not shown anti-SARS-CoV-2 activity, darunavir exhibits promising binding affinity against M^pro^ based on in silico studies [25,26,27].

Interestingly, approximately 23% of the screened metabolites (i.e., 59 compounds) demonstrated higher binding affinity than that of darunavir with a docking score of −8.2 kcal/mol. 2D docking poses of those fifty-nine diterpenes with the key residues inside the M^pro^ active site are presented in Figure S1 (Supplementary Materials). Most of the investigated metabolites displayed similar M^pro^ binding modes inside the M^pro^’s binding pocket, exhibiting an essential hydrogen bond with GLU166, resulting in high binding affinities (Figure S1). Estimated docking scores, 2D chemical structures, in addition to binding features for the top potent metabolites in complex with M^pro^ are listed in Table 1.

**Figure 1:**
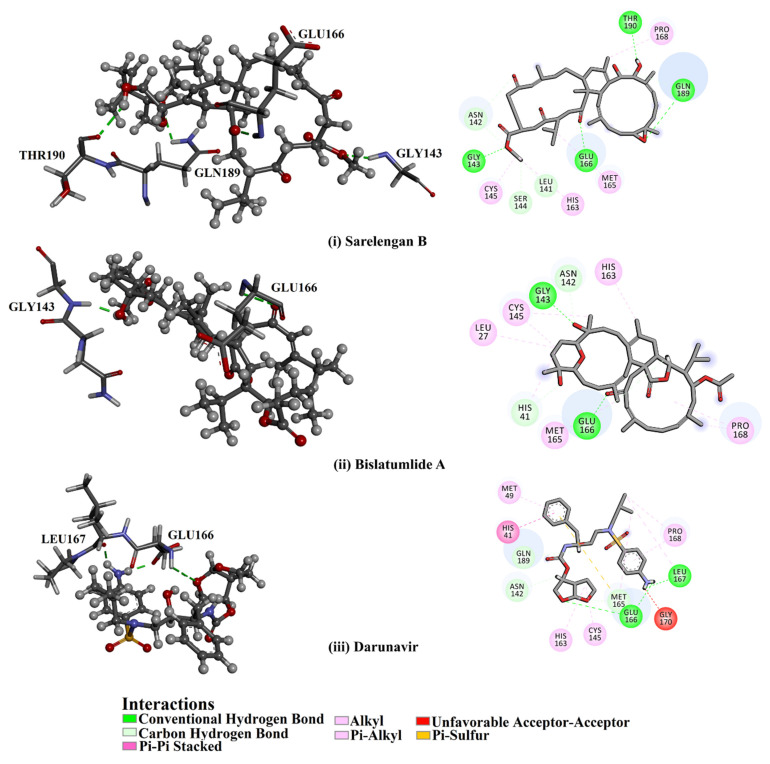
3D and 2D representations of predicted binding modes of *Sarcophyton* cembranoid diterpenes metabolites (**i**) sarelengan B (**363**), (**ii**) bislatumlide A (**340**) and (**iii**) darunavir against SARS-CoV-2 main protease (M^pro^).

From the data in Table 1, it is apparent that **363**, **340**, **347**, **345** and **357** demonstrated solid binding affinities against M^pro^ with docking scores in the range of –8.7 to –9.8 kcal/mol. High docking scores are ascribed to the capability of forming hydrogen bonds, van der Waals, hydrophobic and pi-based interactions with the fundamental amino acid residues within the M^pro^ active site. Sarelengan B (**363**), separated from *S. elegans,* demonstrated the highest binding affinity of the diterpenes towards M^pro^ with a docking score of −9.8 kcal/mol. Investigating the binding mode of **363** inside the M^pro^ active site revealed that two carbonyl groups of (*E*)-cyclotetradec-9-ene-1,4,8-trione formed three hydrogen bonds with the imidazole ring backbone of HIS41, SH group of CYS145 and NH group of GLU166, with bond lengths of 2.01, 2.34 and 2.68 Å, respectively (Figure 1 and Table 1). Additionally, a hydroxy group of **363** interacts with the backbone carbonyl group of GLU166 with a bond length of 2.35 Å (Figure 1 and Table 1).

Bislatumlide A (**340**), separated from *S. latum*, manifested the second-highest binding affinity against M^pro^ with a docking score of −9.6 kcal/mol. Investigating the binding mode of **340** towards the M^pro^ showed that the methanol hydroxy group exhibits a hydrogen bond with the backbone NH of GLY143 with a bond length of 1.88 Å (Figure 1 and Table 1). While the carbonyl group of dihydrofuranone shares a hydrogen bond with the backbone NH group of GLU166 with a bond length of 2.68 Å (Figure 1 and Table 1).

It is worth noting that the five potent metabolites containing a cyclotetradecane-trione ring revealed the vital role of the chemical nucleus in the binding affinity of cembranoids with M^pro^.

Darunavir demonstrated a satisfactory binding affinity (−8.2 kcal/mol), exhibiting three hydrogen bonds with LEU167 and GLU166 with bond lengths of 1.96, 2.88 and 1.94 Å, respectively (Figure 1 and Table 1). More precisely, the NH_2_ of the aniline ring forms two hydrogen bonds with the carboxylate group of GLU166 and carbonyl group of LEU167 with bond lengths of 1.94 and 1.96 Å, respectively. Furthermore, the oxygen atom of the tetrahydrofuran ring exhibits a hydrogen bond with the backbone NH of GLU166 with a bond length of 2.88 Å. A docking comparison of darunavir with **363** and **340** disclosed promising binding affinities of the two metabolites as M^pro^ inhibitors.

### 2.2. MD Simulations and Binding Energy Calculations

**Figure 2:**
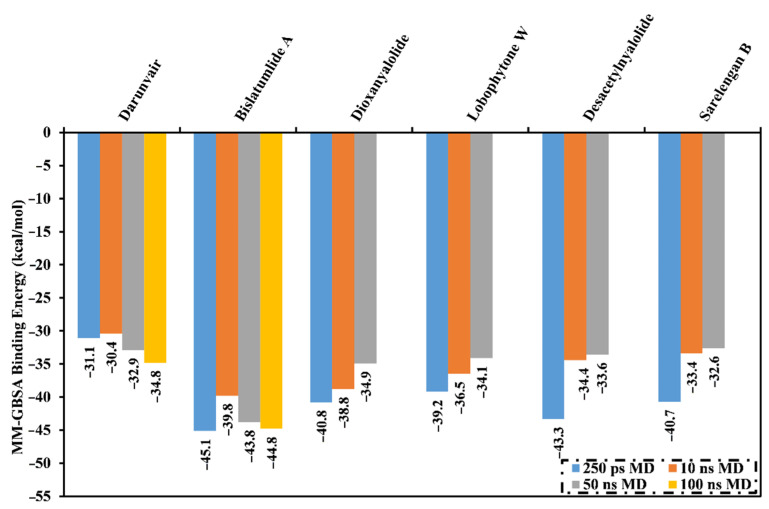
Average MM-GBSA binding energies for darunavir and the most potent diterpenes in complex with SARS-CoV-2 (M^pro^) over 250 ps MD simulation in an implicit water solvent and 10 ns, 50 ns and 100 ns MD simulations in an explicit water solvent.

Molecular dynamics (MD) simulations provide structural details, stability of the ligand-enzyme complexes, conformational flexibilities and the reliability of ligand-enzyme binding affinities [28,29]. The most potent diterpenes (59 compounds with docking scores <−8.2 kcal/mol) in complex with M^pro^ were subjected to MD simulations and binding free energy calculations. To lessen the computational cost and time, the MD simulations were executed in the implicit water solvent for 250 ps in addition to the MM-GBSA approach was used to compute the corresponding binding free energies (see computational methodology section for details). The calculated MM-GBSA binding affinities for the selected metabolites are listed in Table S2. As shown in Table S2, five inhibitors demonstrated lower binding energies (Δ*G*_binding_) than that of darunavir (calc. −31.0 kcal/mol). Generally, the computed MM-GBSA binding energy values were lower than the corresponding docking scores, which might be ascribed to the different evaluation functions of the two employed methods. An over estimation of binding energy using a MM-GBSA approach can occur when not considering the contribution due to entropy [30,31]. To gain more reliable binding affinities of diterpene metabolites in complex with M^pro^, those metabolites were further submitted to 10 ns MD simulations in an explicit water solvent. In addition, the corresponding binding energies were calculated (Figure 2).

What is interesting about the data in Figure 2 is that all investigated metabolites revealed lower binding energies (Δ*G*_binding_) than that of darunavir (calc. −30.4 kcal/mol). Therefore, those potent metabolites were selected and subjected to 50 ns MD simulations in the explicit water solvent, followed by MM-GBSA binding energy calculations (Figure 2). As shown in Figure 2, only **340** demonstrated steady diminution in the binding energies over the simulation times. However, the other investigated molecules exhibited a slight rise in MM-GBSA binding energies throughout the MD simulations. For instance, the evaluated MM-GBSA binding energies for **340** towards M^pro^ were −45.1, −39.8 and −43.8 kcal/mol throughout 250 ps implicit-solvent MD, 10 ns explicit-solvent MD and 50 ns explicit-solvent MD simulations, respectively. This displays the significance of long MD simulations to anticipate reliable binding affinity of the diterpenes with M^pro^. MD simulation for **340** complexed with M^pro^ was then elongated to 100 ns. In addition, the corresponding MM-GBSA binding energy was evaluated (Figure 2).

**Figure 3:**
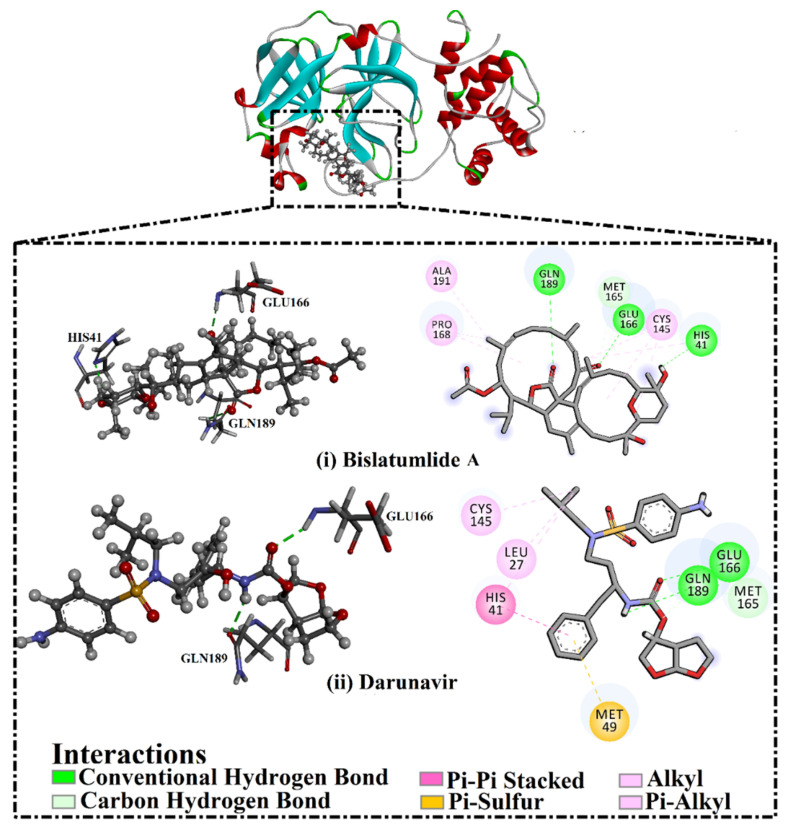
3D and 2D representations of binding modes of (**i**) bislatumlide A (**340**) and (**ii**) darunavir in complex with SARS-CoV-2 main protease (M^pro^) on the basis of the average structure throughout the 100 ns MD simulation.

What is striking about Figure 2 is that there was no appreciable variation between the estimated MM-GBSA binding energy for **340** complexed with M^pro^ over the 50 and 100 ns MD simulations. Comparing the binding affinities of **340** and darunavir, **340** demonstrated strong binding affinity throughout the 100 ns MD simulation against M^pro^ with an average Δ*G*_binding_ of −34.8 kcal/mol. The outstanding potency of **340** was attributed to its capability of exhibiting significant hydrogen bonds, hydrophobic, pi-based interactions and van der Waals interactions with the key amino acid residues within the M^pro^ active site. The average structures for **340** and darunavir inside the active site throughout the 100 ns MD simulations are depicted in Figure 3. The most exciting finding was that **340** preserved three hydrogen bonds with the fundamental amino acid residues of M^pro^ throughout the 100 ns MD simulation (Figure 3). Darunavir also showed satisfactory binding energy over the 100 ns MD simulation towards M^pro^ with an average Δ*G*_binding_ of −34.8 kcal/mol, exhibiting only two hydrogen bonds with the key amino acid residues of M^pro^ (Figure 3). In conclusion, the MM-GBSA binding energy calculations displayed a remarkably higher binding affinity of **340** compared to darunavir.

The MM-GBSA scheme identifies different components the participate in the total binding energy (Δ*G*_binding_), including van der Waals interactions (Δ*E*_vdw_), electrostatic interactions (Δ*E*_ele_), electrostatic solvation free energy evaluated from the generalized Born equation (Δ*E*_GB_), the nonpolar component of the solvation energy (Δ*E*_SUR_), total gas-phase energy (Δ*G*_gas_) and solvation free energy (Δ*G*_Solv_). All these binding free energy components of **340** and darunavir with SARS-CoV-2 M^pro^ are summarized in Table 2.

Based on the evaluated MM-GBSA binding energies, van der Waals interactions (Δ*E*_vdw_) were found to be the prime force inducing molecular complexation with M^pro^ for both bislatumlide A (Δ*E*_vdw_ of −56.1 kcal/mol) and darunavir (Δ*E*_vdw_ of −47.4 kcal/mol). Additionally, electrostatic interactions (*E*_ele_) were an appropriate contributor for **340**- and darunavir-M^pro^ binding affinities with an average value of −27.7 and −15.1 kcal/mol, respectively. It is noted that *E*_vdw_ is approximately two-fold more robust than the Δ*E*_ele_. Together these results provide quantitative data of the binding affinities of **340** and darunavir as putative M^pro^ inhibitors.

**Figure 4:**
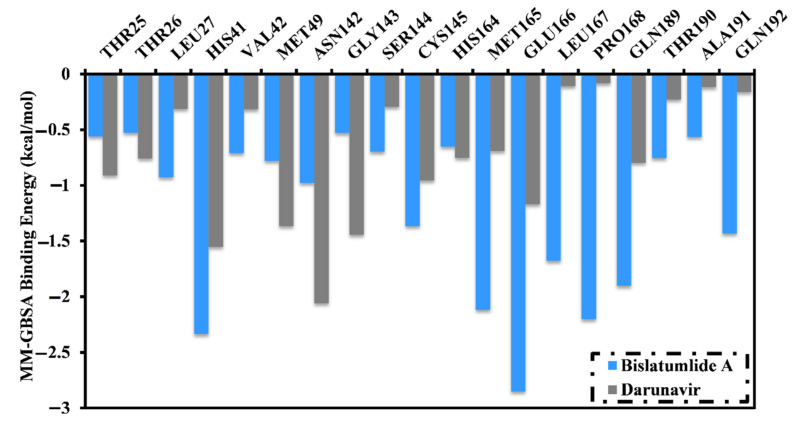
Per-residue decomposition of the total binding free energy (kcal/mol) of bislatumlide A (**340**) and darunavir in complex with SARS-CoV-2 M^pro^.

Moreover, to explore the key residues that exhibit essential contributions to **340**- and darunavir-M^pro^ interactions, the per-residue decomposition of the binding free energy calculations was executed. All the residues with energetic contributions <−0.50 kcal/mol were considered and presented in Figure 4. It is apparent that HIS41, GLY143, MET165, GLU166 and GLN189 amino acid residues contributed to the interactions of **340** and darunavir with M^pro^. A considerable contribution of the GLU166 amino acid residue to the total binding free energy was observed with values of −2.9 and −1.2 kcal/mol for **340**- and darunavir-M^pro^ complexes, respectively. In addition, the hydrophobic residues participate in higher binding affinity as a result of hydrophobic interactions between **340** and the hydrophobic residues.

Mining of naturally occurring plant-based compounds and marine-derived metabolites has evoked an upsurging interest in discovering potential SARS-CoV-2 M^pro^ inhibitors. A comparison of the binding affinity of **340** with those previously reported as SARS-CoV-2 M^pro^ inhibitors would give an informative insight into the preferential inclination of **340** as a prospective drug candidate. Towards an adequate comparison, only natural products and marine metabolites identified by employing a similar in silico technique were considered. Among the explored natural products, salvanolic acid and curcumin accentuated appreciable MM-GBSA binding energies against M^pro^ over 40 ns MD course with values of −44.8 and −34.2 kcal/mol, respectively [11]. Notably, rutin, a flavonol glycoside molecule, exhibited a debilitated binding affinity against M^pro^ (Δ*G*_binding_ = −28.4 kcal/mol over 150 ns MD simulation) [7]. Two flavone nominees dubbed as PubChem-129-716-607 and PubChem-885-071-27 showed considerable binding affinities against M^pro^, over 150 ns MD course, with Δ*G*_binding_ values of −69.0 and −68.1 kcal/mol, respectively [7]. Erylosides B, a terpene marine natural product, demonstrated preferential MM-GBSA binding energy with M^pro^ over 100 ns MD simulation (calc. −51.9 kcal/mol) [8]. Comparing the MM-GBSA binding affinity of **340** to the earlier identified compounds robustly unveiled its competing binding affinity as a prospective M^pro^ inhibitor. As a consequence, further investigation of the structural and energetic stability of **340** over 100 ns MD simulation is desired.

### 2.3. Post-MD Analyses

Structural and energetic analyses were conducted throughout the 100 ns MD simulations, as well as compared to those of darunavir to further emphasized the stability and behavior of **340** in complex with M^pro^. For structural and energetic analyses, four characteristics were evaluated from respective simulation trajectories: root-mean-square deviation (RMSD), hydrogen bond length, binding energy per frame and center-of-mass (CoM) distance.

#### 2.3.1. Binding Energy per Frame

**Figure 5:**
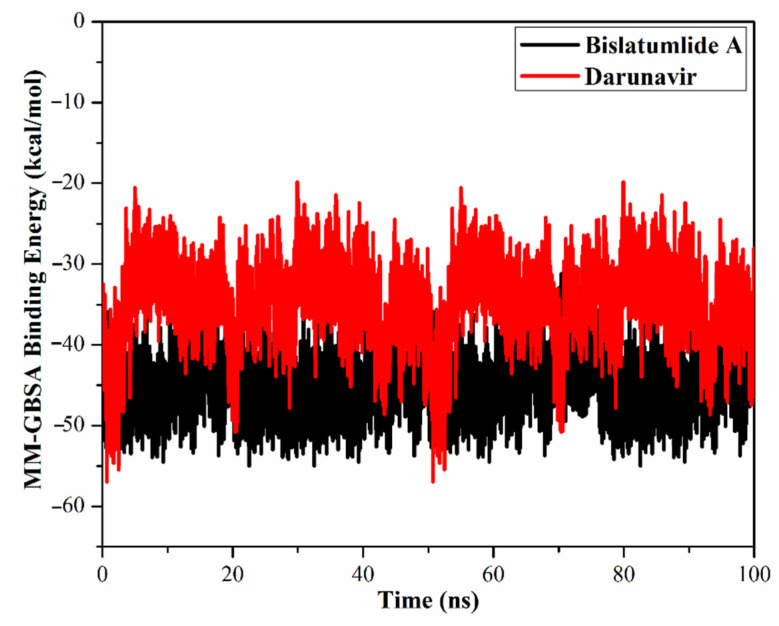
Estimated MM-GBSA binding energies per frame for bislatumlide A (**340**) (black) and darunavir (red) in complex with SARS-CoV-2 M^pro^ throughout 100 ns MD simulations.

The correlation between single-trajectory MM-GBSA binding energy and time was used to evaluate the comprehensive structural stability of **340** and darunavir in complex with M^pro^ throughout the 100 ns MD simulations (Figure 5). An interesting aspect of this graph is the general stabilities for **340** and darunavir with average binding energies (Δ*G*_binding_) of −44.8 and −34.8 kcal/mol, respectively. The most obvious finding to emerge from the analysis is that all complexes maintained stability over 100 ns MD simulations.

#### 2.3.2. Hydrogen Bond Length

Hydrogen bonds have a vital role in preserving the binding of investigated compounds with a protein. Consequently, hydrogen bond analysis was carried out for **340** and darunavir in complex with M^pro^ throughout the 100 ns MD simulations (Table 3). Table 3 demonstrated that the two inhibitors manifested stable hydrogen bonds with GLU166 with H-bond occupancy values of 90.3% and 85.7% for bislatumlide A and darunavir, respectively. The high H-bond occupancy confirms the significant role of GLU166 within the active site of M^pro^. A comparison of the data, summarized in Table 3, reveals a higher stability for **340** than darunavir. More exactly, **340** exhibited three stable hydrogen bonds with HIS41, GLU166 and GLN189 with an average H-bond distance of 2.9, 2.8 and 2.6 Å, respectively. However, darunavir formed only a stable hydrogen bond with GLU166 with an average H-bond distance of 2.7 Å. The high stability for the bislatumlide A-M^pro^ complex compared to darunavir-M^pro^ complex is clearly supported by the current findings.

#### 2.3.3. Center-of-Mass Distance

**Figure 6:**
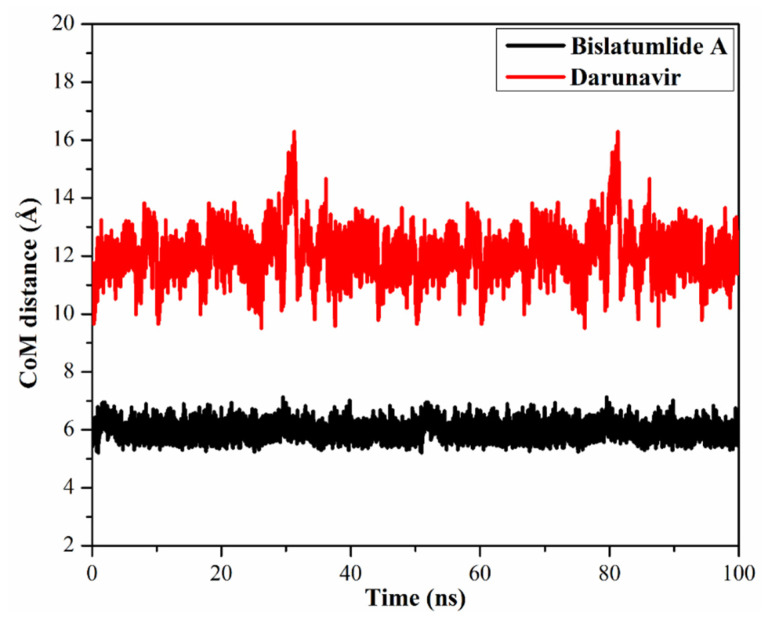
Center-of-mass (CoM) distances (in Å) between bislatumlide A (black) and darunavir (red) and GLU166 of M^pro^ throughout 100 ns MD simulations.

Center-of-mass (CoM) distances were adopted to allow a deeper insight into the stability of inhibitor-M^pro^ complexes over the 100 ns MD simulations (Figure 6). From the data, it is apparent that CoM distances were more consistent for **340** complexed with M^pro^ compared to darunavir with average values of 6.0 and 12.1 Å, respectively. The current results propose that **340** bound more tightly to the M^pro^ complex compared to darunavir.

#### 2.3.4. Root-Mean-Square Deviation

**Figure 7:**
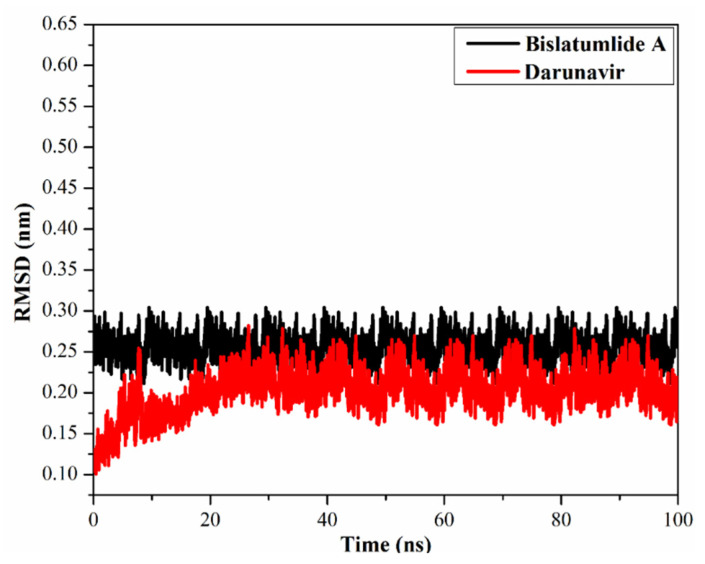
Root-mean-square deviation (RMSD) of the backbone atoms from the initial structure of bislatumlide A (black) and darunavir (red) with M^pro^ over a 100 ns MD simulations.

The root-mean-square deviation (RMSD) of the whole complex backbone atoms were plotted as a function of time to examine the structural stability of the **340** and darunavir-M^pro^ complexes throughout the simulation time (Figure 7). RMSD analysis demonstrated that the scrutinized complexes initiated stabilization after 20 ns and kept their stabilities until the end of the 100 ns MD simulations. The evaluated RMSD values for these complexes stayed beneath 0.26 nm throughout the MD simulation time. Overall, the current results demonstrated that **340** is tightly bonded and does not influence the structural stability of the M^pro^, as well as conserved structural integrity.

### 2.4. In Silico Drug-Likeness

The efficiency of curative drugs fundamentally relies on the molecular property and bioactivity of the molecules [32] was examined. To consider the drug-likeness in addition to bioactivity of **340**, in silico molecular features were evaluated using a SwissADME web server and compared to those of darunavir. The predicted properties are shown in Table 4.

The permeability through the cell membrane, as inspected by the mlogP value, was less than five (4.3 and 1.2 for bislatumlide A and darunavir, respectively), proposing that these inhibitors have adequate membrane permeability. Additionally, the number of hydrogen bond donors (nOHNH) and acceptors (nON) were less than 5 and 10, respectively. Moreover, the molecular weights for bislatumlide A and darunavir were 694.9 and 547.7, respectively, suggesting that these inhibitors are readily transported and/or diffused in the absorption process. Another parameter pointing out the molecular bio-absorption is the topological polar surface area (TPSA). The TPSA of **340** and darunavir were 119.4 and 148.8 Å, respectively, indicating a satisfactory cell membrane permeability and oral bioavailability level.

### 2.5. Molecular Target Prediction and Network Analysis

**Figure 8:**
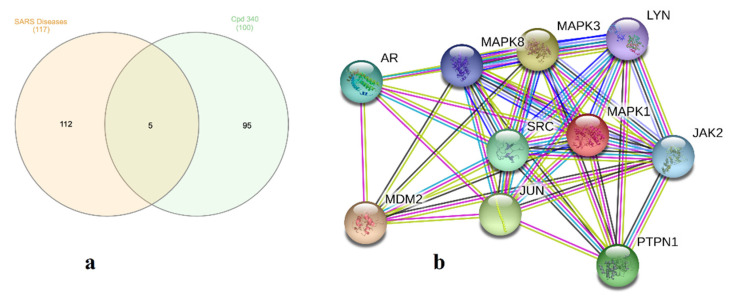
(**a**) Venn diagram analysis for bislatumlide A (**340**) towards SARS disease genes and (**b**) STRING PPI network for the top targets known by network analyzer for bislatumlide A (**340**) as a SARS-CoV-2 main protease (M^pro^) inhibitor.

One hundred and seventeen genes for severe acute respiratory syndrome diseases (SARS, C1175175) were recorded utilizing DisGeNET online tools. Using Venn diagram comparison analysis, frequently participated genes for **340** involved PRKCA, PRKCB, MAPK1 and MAPK14. A STRING database was utilized to recognize protein-protein interactions for **340** included the biochemical signaling genes AR, MAPK1, MAPK3, MAPK8, LYN, JAK2, SRC, MDM2 PTPN1 and JUN as an in silico natural-product inhibitor towards M^pro^ and supplies a ligand lead for in vitro enzyme investigations (Figure 8 and Table S3).

### 2.6. Pathway Enrichment Analysis (PEA)

**Figure 9:**
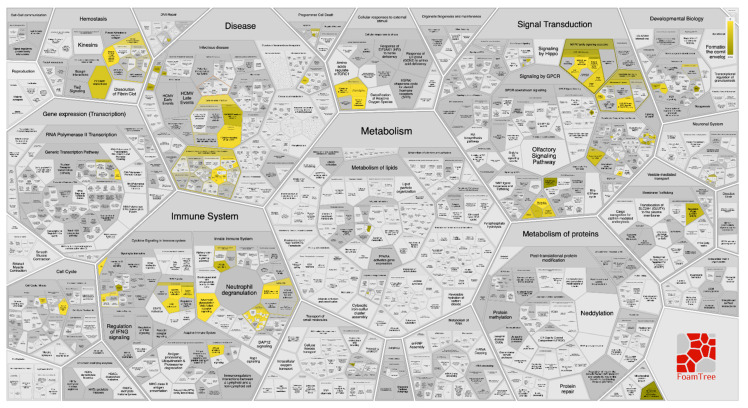
FoamTree diagram showing a genome-wide enriched pathways overview based on Voronoi tessellation analysis for the top 10 genes targets stimulated by bislatumlide A (**340**) as a proposed SARS-CoV-2 inhibitor.

Toward better genome-wide mining for **340**, stimulated targets and their interactors, were determined using pathway enrichment analysis (PEA) and Boolean network analysis (BNA). A genome-wide hierarchy map representation of the pathways affected by **340** treatment was constructed (Figure S2). The hierarchy map showed that among the most stimulated pathways was the “disease” pathway, particularly the potential therapeutic for the SARS pathway. In addition, a reacfoam tree of the top targeted/influenced pathways by the top 10 gene targets stimulated by **340** treatment against SARS-CoV-2 infection was constructed (Figure 9). Although the PEA results revealed that the top 10 gene targets stimulated by **340** were identified in 833 reactome pathways (each pathway was hit by at least one of them), the top three significantly enriched major pathways are (A) signal transduction pathway, (B) disease pathway and (C) immune system pathway, with a false discovery rate (FDR) of <0.00001%. Under these pathways, the interaction between the top 10 genes/signals stimulated with **340** treatment was visualized and mapped (Figure S3). Additionally, interactors with these top 10 genes are listed (Table S4).

**Figure 10:**
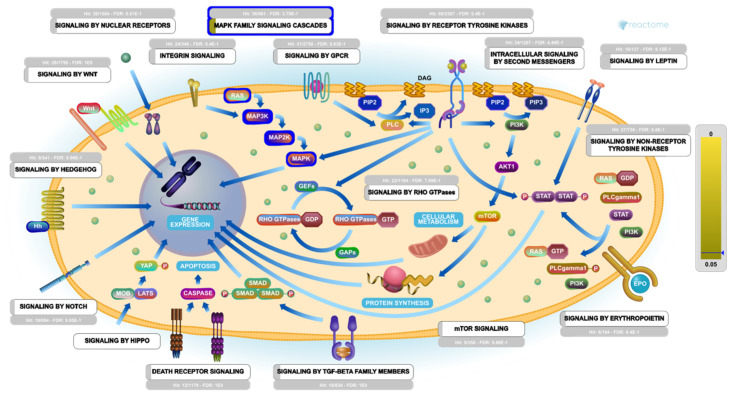
A diagrammatic model shows the Signal Transduction Reactome Network.

Remarkably, under the most enriched major pathway (signal transduction), it was found that the MAPK family signaling cascades pathway was on the top of the most enriched pathway influenced by **340** within the human biological system (Figure 10). Mining of the PEA analysis outcomes indicated that a set of five genes (MAPK1, MAPK3, JAK2, JUN and SRC) were significantly modulated as biological targets to **340** as potent SARS-CoV-2 inhibitor. Furthermore, the interactome results showed that these five genes were found to interact with other 48 genes/interactors (Table S4).

Previous studies show that various alterations in a few signaling pathways can control many central biological functions of the cell after SARS-CoV-2 infection [33,34]. Recently, several research reports found that the p38 mitogen-activated protein kinase (MAPK), nuclear factor kappa-light-chain-enhancer of activated B cells (NF-κB) and epidermal growth factor receptor (EGFR) signaling pathways are altered following coronavirus infection. These pathways play a crucial role in oppressing the host antiviral response and are essential for coronavirus entry, replication and apoptosis [35].

Indeed, accumulated evidence confirmed that the p38 MAPK signaling pathway can operate various essential biological activities depending on the stimuli and the type of stimulated tissue. Therefore, modulation of p38 MAPK signaling was found to raise cell death and survival [36]. Additionally, many reports emphasized the critical role of p38 MAPK signaling in several viral infections targeting the respiratory system; in HCoV-229E, the triggering of p38 MAPK is required to induce cytopathic effect (CPE), as well as the viral replication process. In addition, the upregulation of p38 MAPK may also promote the viral entry through the ACE2 endocytosis and cause subsequent inflammations, thrombosis and could initiate multi-organ failure in COVID-19 patients [37].

Notably, one of the most common consequences of SARS-CoV-2 infection is pulmonary injury. In COVID-19 patients, the p38 MAPK signaling pathway, via p53, or its alternatives TGF-β1, or syntenin, drives apoptosis and ends with lung injury, which means that SARS-CoV-2 is likely to modulate the p38 MAPK signaling pathway to provoke the apoptosis and lung damage. Here, based on the Reactome mining, we found that **340** showed a potential therapeutic effect (Hit:32/144 with FDR:9.17E-1) against SARS-CoV-2 viral infection (Figure S4). This potentiality may be attributed to **340** capability to re-modulate the p38 MAPK signaling pathway hijacked by SARS-CoV-2 viral infection and antagonize its harmful effects. This speculation was supported by recent reports that demonstrated the possibility of inhibiting the p38 MAPK signaling pathway as a promising therapeutic strategy against the SARS-CoV-2 pandemic [38,39].

## 3. Materials and Methods

### 3.1. M^pro^ Preparation

A high-resolution (2.16 Å) biological unit for the X-ray crystallographic structure of SARS-CoV-2 main protease (M^pro^) deposited in Protein Data Bank by Jin et al. (PDB ID: 6LU7 [17]) in complex with peptidomimetic inhibitor (N3) was retrieved and utilized as a template for all molecular docking calculations and molecular dynamics (MD) simulations. The protein structure was prepared by eliminating all crystallographic water molecules, heteroatoms and ions, keeping only the amino acid residues. H++ webserver was applied to assign the protonation states of M^pro^ [40]. Additionally, all missing hydrogen atoms were added. In H++ calculations, physiologic conditions of external dielectric constant = 80, pH = 7, salinity = 0.15 and internal dielectric constant = 10 were used to evaluate the pK_a_ values of M^pro^ amino acid residues.

### 3.2. Inhibitor Preparation

A chemical library containing metabolites from the genus *Sarcophyton* reported over the last twenty years (1998–2019) [18] was assembled. Structures were retrieved from the PubChem database (https://pubchem.ncbi.nlm.nih.gov) in SDF format, except for 2D structures of bislatumlides A and B that were taken from the original article [41]. Omega2 software was used to generate three-dimensional (3D) structures of the investigated compounds [42,43]. Geometrical structures were minimized with the assistance of Merck Molecular Force Field 94 (MMFF94S), implemented inside SZYBKI software [44,45]. Undefined stereocenters were enumerated with the help of flipper application inside Omega2 software. A conformational search was performed to generate all conformers within the energy window value of 10 kcal/mol. The lowest energy conformer was subjected to minimization with the assistance of Merck Molecular Force Field 94 (MMFF94S), implemented inside SZYBKI software [44,45]. The protonation state and tautomer enumeration of the compounds were examined by fixpka and tautomer applications, respectively, included in the QUACPAC software [46]. Two-dimensional (2D) structures are presented in Table S1.

### 3.3. Molecular Docking

In the current study, AutoDock4.2.6 software was applied to perform all molecular docking calculations [47]. According to AutoDock protocol [48], the pdbqt file of SARS-CoV-2 main protease (M^pro^) was prepared. In AutoDock4.2.6, the maximum number of energy evaluations (*eval*) and the number of genetic algorithm (*GA*) run variables were set to 250 and 25,000,000, respectively. All other docking parameters were preserved at their default settings. The size of the box (60 Å × 60 Å × 60 Å) was specified to encompass the SARS-CoV-2 M^pro^ active site appropriately. The binding site of SARS-CoV-2 M^pro^ was precisely located based on the availability of resolved structures of M^pro^ in complex with inhibitors [49]. An AutoGrid4.2.6 program was applied to generate maps with a grid spacing value of 0.375 Å. The coordinates of the grid center were placed at −13.069, 9.740 and 68.490 (XYZ assignments, respectively). The Gasteiger method was utilized to compute the atomic partial charges of the studied compounds [50]. The anticipated binding modes for each compound were handled using the built-in clustering analysis (1.0 Å RMSD tolerance) and conformation with the lowest energy within the largest cluster was opted as a representative pose.

### 3.4. Molecular Dynamics Simulations

Molecular dynamics (MD) simulations were carried out for the most potent diterpenes in complex with M^pro^ using AMBER16 software [51]. An AMBER force field of 14SB was employed to describe the M^pro^ catalytic site [52], while the general AMBER force field (GAFF2) [53] was used to characterize individual metabolites. Both implicit-solvent and explicit-solvent MD simulations were performed. In the implicit-solvent MD simulations, an AM1-BCC method with the assistance of the Antechamber tool implemented inside AMBER16 software was used to assign the atomic partial charges of the diterpenes [54]. Neither cutoff distance for nonbonded nor periodic boundary conditions were applied. Furthermore, the solvation influence was considered by utilizing igb = 1 solvent model [55]. Energy minimization was initially executed on the docked diterpene in complex with M^pro^ for 500 steps. Gentle heating was carried out as well on the minimized complexes from 0 K to 300 K over 10 ps NVT MD simulations. The production stage was then performed over 250 ps, in addition to snapshots recorded every 1 ps. Therefore, 250 snapshots were derived from each MD simulation. Herein, the CPU version of pmemd (pmemd.MPI) implemented inside AMBER16 software was employed to conduct all implicit-solvent MD simulations.

In explicit-solvent MD simulations, atomic partial charges of the investigated metabolites were assigned at the HF/6-31G* level with assistance of Gaussian09 software with the restrained electrostatic potential (RESP) fitting approach [56,57]. A water-solvated cubic box was constructed using a TIP3P water model with a minimum distance to the box edge of 15.0 Å (1.5 nm). All solvated systems were neutralized by adding Na^+^ or Cl^−^ counter-ions [58]. The maximum number of energy minimization steps were adjusted to 5000 utilizing combined steepest and conjugate gradient algorithms. The minimized systems were thereafter heated gradually to 300 K over 50 ps in the NVT ensemble. Additionally, the systems were sufficiently equilibrated for 1 ns under NPT conditions. The production stages were subsequently executed under an NPT ensemble for each studied M^pro^-metabolite complex over simulation times of 10 ns, 50 ns and 100 ns. Snapshots were gathered every 10 ps for post-MD analyses. The long-range electrostatic interactions were estimated using the Particle Mesh Ewald (PME) method. A short-range nonbonded interaction cutoff distance of 12 Å was applied [59]. The gamma_ln collision frequency applied was 1.0 ps^−1^; in addition, the Langevin thermostat was utilized to maintain the temperature at 298 K [60]. The pressure was controlled using a Berendsen barostat with a pressure relaxation time of 2 ps [61]. All bonds, including hydrogen atoms, were constrained using a SHAKE algorithm with a time step of 2 fs [62]. All explicit-solvent MD simulations were executed with the help of a GPU version of pmemd (pmemd.cuda) implemented inside an AMBER16 package. All in silico calculations, including molecular docking calculations, quantum mechanics calculations and MD simulations, were executed on the CompChem GPU/CPU hybrid cluster (hpc.compchem.net). Molecular graphics and analyses were executed with the assistance of BIOVIA DS Visualize 2020 [63].

### 3.5. MM-GBSA Binding Energy Calculations

The binding free energies for the most potent metabolites that complexed with M^pro^ were computed using molecular mechanical-generalized Born surface area (MM-GBSA) approach implemented inside AMBER16 software [64]. The MM-GBSA (Δ*G*_binding_) binding free energy calculations were estimated based on the snapshots collected from MD simulations. The average binding free energy (Δ*G*_binding_) was evaluated as follows:

Δ*G*_binding_ = *G*_complex_ − (*G_Sarcophyton_* + *G*_Mpro_) (1)

where the energy term (*G*) is estimated as:

*G* = *E*_ele_ + *E*_vdw_ + *G*_SA_ + *G*_GB_ (2)

*E*_ele_ and *E*_vdw_ are electrostatic and van der Waals energies, respectively. *G*_SA_ stands for the nonpolar solvation-free energy, generally estimated via a linear function of the solvent-accessible surface area (SASA) using a LCPO algorithm [65]. *G*_GB_ is the electrostatic solvation free energy computed from the generalized Born equation. The polar component of the desolvation energy was assigned via Onufriev’s GB [66] (igb = 2). The solvent (exterior) and solute (interior) dielectric constants were set to 80 and 1, respectively. A single-trajectory method was applied, in which the coordinates of each metabolite-M^pro^, M^pro^ and metabolite were obtained from a single trajectory. The configurational entropy (TS) is typically neglected due to the higher computational costs [30,31].

### 3.6. Drug-Likeness Properties

For the identified cembranoids, physicochemical properties including molecular weight (MW), octanol/water partition coefficient (LogP), hydrogen bond acceptor (HBA), topological polar surface area (TPSA), rotatable bond count (RB) and hydrogen bond donor (HBD) were estimated with assistance of SwissADME web server (http://www.swissadme.ch/).

### 3.7. Protein Interactions Network and Pathway Enrichment Analysis (PEA)

Cembranoids were screened via an online website-based tool of SwissTargetPredicition (http://www.swisstargetprediction.ch) to recognize the possible targets for each ligand. InteractiVenn online tool was applied to design Venn Diagram [67] for accessible database for severe, acute respiratory syndrome diseases (DisGeNET database; https://www.disgenet.org). The top 100 genes for the most potential metabolite were retrieved. A functional database of STRING for top anticipated targets was then used to generate protein-protein interaction (PPI) [68].

Cytoscape 3.8.2 was used to inspect all probable target-function relations according to a network topology [69]. Furthermore, to explore all potential target-functions relationships for the top 10 targeted genes, pathway enrichment analysis was accomplished using Cytoscape 3.8.2 [69]. In addition, to investigate the impact of bislatumlide A (**340**) as a COVID-19 inhibitor candidate on human biological pathways computationally, Boolean-network modeling was implemented. The fuzzy logic simulation approach [70] was implemented to mimic Boolean-network dynamic behavior, which consequently transformed from Reactome pathways by extending two-state Boolean variables to variables with values ranging from zero to one. In addition, a FoamTree representation based on Voronoi tessellation analysis for the top 10 gene targets stimulated by **340** was constructed. Finally, a Reactome FIViz plugin tool was utilized for genome-wide visualization of all possible drug-targets interactions [71].

## 4. Conclusions

COVID-19 is a universal risk for positive human health and economic losses continue to build unabated. A rapid proceed, especially in vaccine and drug development, is substantial to conquer the further outbreak in prevalence and loss of human life from COVID-19 disease. Herein, *Sarcophyton* cembranoid diterpenes were screened as prospective M^pro^ inhibitors using combined molecular docking and molecular dynamics approaches. Based on molecular docking calculations, MD simulations, as well as molecular mechanics-generalized born surface area (MM-GBSA) binding energy calculations, **340** manifested a convenient binding affinity with Δ*G*_binding_ < −44.0 kcal/mol against M^pro^. The energetic and structural analyses throughout 100 ns MD simulations emphasized the high stability of **340**. Drug-likeness properties for **340** were evaluated and demonstrated favorable physicochemical properties. The compound has the potential to re-modulate the p38 MAPK signaling pathway. In vitro and in vivo explorations are predicted to further identify the role of **340** as a potential inhibitor therapeutic for COVID-19 treatment.

## Figures and Tables

**Table 1:** Calculated binding affinities (in kcal/mol), 2D chemical structures and binding features for darunavir and the top five most promising cembranoid diterpenes against SARS-CoV-2 main protease (M^pro^) ^a^.

Compound Name (Number)	Genus	2D Chemical Structure	Docking Score (kcal/mol)	Binding Features ^b^
Darunavir	*---*	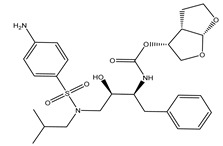	−8.2	LEU167 (1.96 Å), GLU166 (1.94, 2.88 Å)
Sarelengan B (**363**)	*S. elegans*	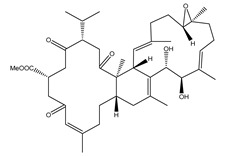	−9.8	GLY143 (2.39 Å), GLU166 (1.94 Å), GLN189 (2.58 Å), THR190 (2.33 Å)
Bislatumlide A (**340**)	*S. latum*	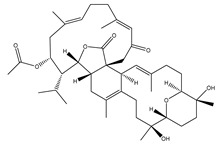	−9.6	GLY143 (1.88 Å), GLU166 (2.68 Å)
Dioxanyalolide (**347**)	*S. elegans*	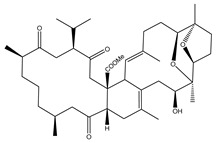	−9.5	GLU166 (2.07 Å)
Desacetylnyalolide (**345**)	*S. elegans*	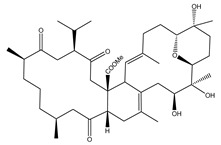	−9.1	GLU166 (1.66, 2.12 Å), THR190 (2.42 Å)
Lobophytone W (**357**)	*S. elegans*	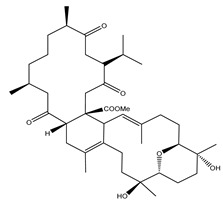	−8.7	HIS41 (2.01 Å), CYS145 (2.34 Å), GLU166 (2.35, 2.86 Å)

^a^ The potent *Sarcophyton* cembranoid diterpenes metabolites were selected based on latter MM-GBSA binding energy calculations. ^b^ Only hydrogen bonds (in Å) were displayed.

**Table 2:** Components of the MM-GBSA binding energies for **340**- and darunavir- SARS-CoV-2 main protease (M^pro^) complexes over 100 ns MD simulations.

Compound Name	Estimated MM-GBSA Binding Energy (kcal/mol)						
	Δ*E*_vdw_	Δ*E*_ele_	Δ*E*_GB_	Δ*E*_SUR_	Δ*G*_gas_	Δ*G*_Solv_	Δ*G*_binding_
Bislatumlide A (**340**)	−56.1	−27.7	45.6	−6.6	−83.8	39.0	−44.8
Darunavir	−47.4	−15.1	33.8	−6.2	−62.5	27.7	−34.8

**Table 3:** Hydrogen bond distance, angle and occupancy for bislatumlide A (**340**) and darunavir with essential M^pro^ amino acid residues throughout 100 ns MD simulations.

Compound Name	Acceptor	Donor	Angle (Degree) ^a^	Distance (Å) ^a^	Occupied (%) ^b^
Bislatumlide A (**340**)	HIS41@ND1	Bislatumlide A@O-H16	164	2.9	67.9
	GLU166@O	Bislatumlide A@O2-H25	142	2.8	90.3
	GLN189@O	Bislatumlide A@O3-H47	145	2.6	88.9
Darunavir	GLU166@O	Darunavir @O5-H36	151	2.8	85.7

^a^ The hydrogen bonds are investigated via the acceptor-donor atom distance of <3.5 Å and acceptor-H-donor angle of >120°. ^b^ Only hydrogen bonds with occupancy higher than 50% were illustrated.

**Table 4:** Predicted physiochemical parameters and structural descriptors of bislatumlide A (**340**) and darunavir as prospective SARS-CoV-2 M^pro^ inhibitors.

Compound Name	mLogP	TPSA	nON	nOHNH	Nrotb	MWt	%ABS
Bislatumlide A (**340**)	4.3	119.4	8	2	3	694.9	67.8%
Darunavir	1.2	148.8	8	3	13	547.7	57.7%

## Data Availability

The data presented in this study are available in the article/supplementary material.

## Supplementary Materials

The following are available online at https://www.mdpi.com/article/10.3390/md19070391/s1, Figure S1: 2D representations of interactions of darunavir and the top 59 potent *Sarcophyton* cembranoid diterpenes metabolites with the proximal amino acid residues of SARS-CoV-2 main protease (M^pro^), Figure S2: A genome-wide overview of the Reactome Pathway Analysis (RPA) results for the top 20 genes targets stimulated by bislatumlide A (**340**) against SARS-CoV-2, Figure S3: Pathway digram analysis for enzymes/signals of the top 10 genes products/targets stimulated by bislatumlide A (**340**), Figure S4: A diagrammatic model shows the potential therapeutic level of bislatumlide A (**340**) against SARS-CoV-2 viral infection, Table S1: Estimated docking score (in kcal/mol) for darunavir and the investigated *Sarcophyton* cembranoid diterpenes metabolites towards SARS-CoV-2 main protease (M^pro^), Table S2: Computed Autodock and MM/GBSA binding energies (in kcal/mol) for darunavir and the top 58 potent *Sarcophyton* cembranoid diterpenes metabolites against SARS-CoV-2 main protease (M^pro^) over 250 ps implicit solvent MD simulations, Table S3: Network topological analysis for the predicted targets for bislatumlide A (**340**), Table S4: Interactors list showing the top 10 genes stimulated by bislatumlide A (**340**), their UniProt Ids and list of genes Ids which Interacts with SARS-CoV-2.
