# Supplementary material for: Blue Biotechnology: Computational Screening of Sarcophyton Cembranoid Diterpenes for SARS-CoV-2 Main Protease Inhibition
Source: Mar Drugs. 2021 Jul 13;19(7):391. doi: 10.3390/md19070391 (PMC8308023; doi:10.3390/md19070391)
Supplement: Supplementary file 1 [file marinedrugs-19-00391-s001.zip › marinedrugs-1254353-supplementary.pdf]

Article

Blue Biotechnology: Computational Screening of *Sarcophyton* Cembranoid Diterpenes for SARS-CoV-2 Main Protease Inhibition

Mahmoud A. A. Ibrahim [1,*], Alaa H. M. Abdelrahman [1], Mohamed A. M. Atia [2], Tarik A. Mohamed [3], Mahmoud F. Moustafa [4,5], Abdulrahim R. Hakami [6], Shaden A. M. Khalifa [7], Fahad A. Alhumaydhi [8], Faris Alrumaihi [8], Syed Hani Abidi [9], Khaled S. Allemailem [8], Thomas Efferth [10], Mahmoud E. Soliman [11], Paul W. Paré [12], Hesham R. El-Seedi [13,14,15,*] and Mohamed-Elamir F. Hegazy [3,10,*]

[1] Computational Chemistry Laboratory, Chemistry Department, Faculty of Science, Minia University, Minia 61519, Egypt; a.abdelrahman@compchem.net
[2] Molecular Genetics and Genome Mapping Laboratory, Genome Mapping Department, Agricultural Genetic Engineering Research Institute (AGERI), Agricultural Research Center (ARC), Giza 12619, Egypt; matia@ageri.sci.eg
[3] Chemistry of Medicinal Plants Department, National Research Centre, 33 El-Bohouth St., Dokki, Giza 12622, Egypt; tarik.nrc83@yahoo.com
[4] Department of Biology, College of Science, King Khalid University, Abha 9004, Saudi Arabia; hamdony@yahoo.com or mfmostfa@kku.edu.sa
[5] Department of Botany & Microbiology, Faculty of Science, South Valley University, Qena 83523, Egypt
[6] Department of Clinical Laboratory Sciences, College of Applied Medical Sciences, King Khalid University, Abha 61481, Saudi Arabia; ahakami@kku.edu.sa
[7] Department of Molecular Biosciences, The Wenner-Gren Institute, Stockholm University, S-106 91 Stockholm, Sweden; shaden.khalifa@su.se
[8] Department of Medical Laboratories, College of Applied Medical Sciences, Qassim University, Buraydah 51452, Saudi Arabia; f.alhumaydhi@qu.edu.sa (F.A.A.); f_alrumaihi@qu.edu.sa (F.A.); k.allemailem@qu.edu.sa (K.S.A.)
[9] Department of Biological and Biomedical Sciences, Aga Khan University, Karachi 74800, Pakistan; m.haniabidi@gmail.com
[10] Department of Pharmaceutical Biology, Institute of Pharmaceutical and Biomedical Sciences, Johannes Gutenberg University, Staudinger Weg 5, 55128 Mainz, Germany; efferth@uni-mainz.de
[11] Molecular Modelling and Drug Design Research Group, School of Health Sciences, University of KwaZulu-Natal, Westville, Durban 4000, South Africa; soliman@ukzn.ac.za
[12] Department of Chemistry & Biochemistry, Texas Tech University, Lubbock, TX 79409, USA; paul.pare@ttu.edu
[13] Department of Chemistry, Faculty of Science, El-Menoufia University, Shebin El-Kom 32512, Egypt
[14] International Research Center for Food Nutrition and Safety, Jiangsu University, Zhenjiang 212013, China
[15] Pharmacognosy Group, Department of Pharmaceutical Biosciences, Uppsala University, Biomedical Centre, Box 574, 751 23 Uppsala, Sweden
* Correspondence: m.ibrahim@compchem.net (M.A.A.I.); hesham.elseedi@su.se (H.R.E.-S.); elamir77@live.com (M.-E.F.H.); Tel.: +20-10-241-61-444 (M.A.A.I.); Tel.: +46-70-043-4343 (H.R.E.-S.); Tel.: +20-33-371-635 (M.-E.F.H.)

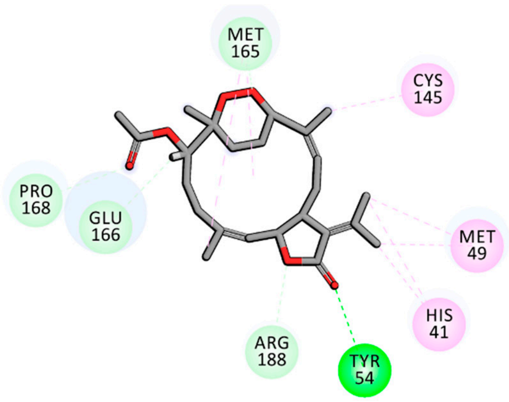

10

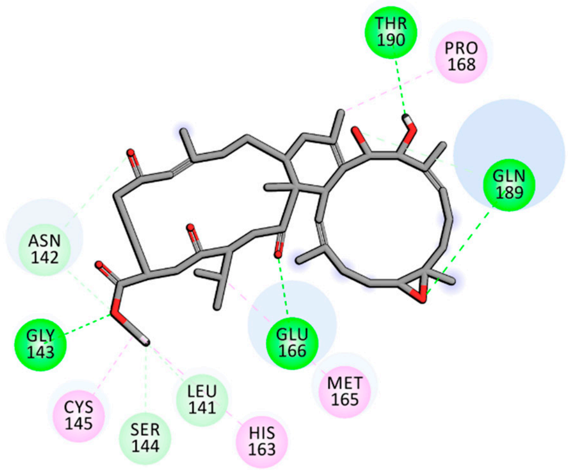

363

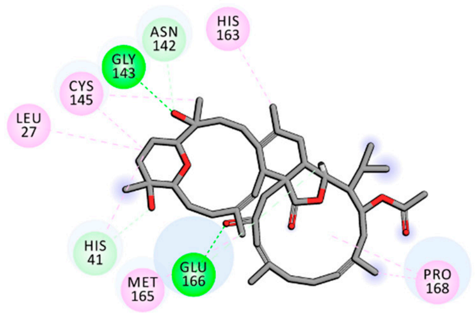

340

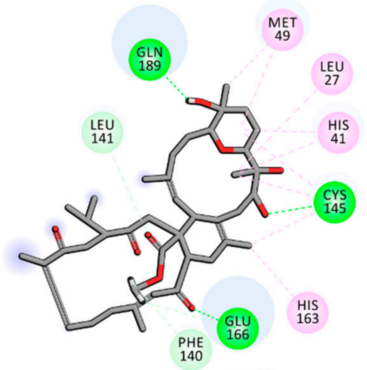

325

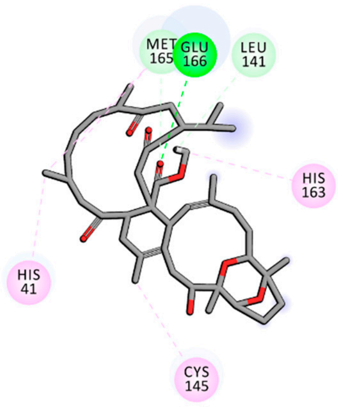

347

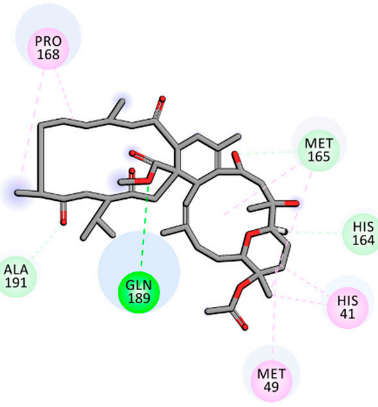

336

Figure S1. 2D representations of interactions of darunavir and the top 59 potent *Sarcophyton* cembranoid diterpenes metabolites with the proximal amino acid residues of SARS-CoV-2 main protease (M^{pro}).

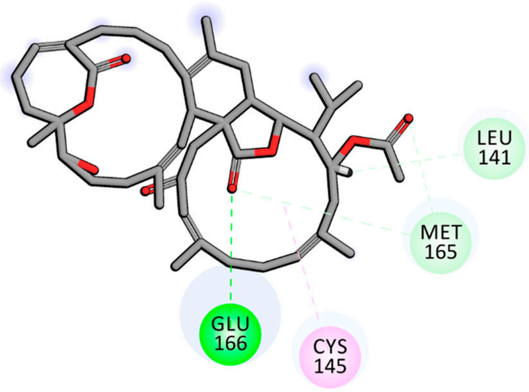

329

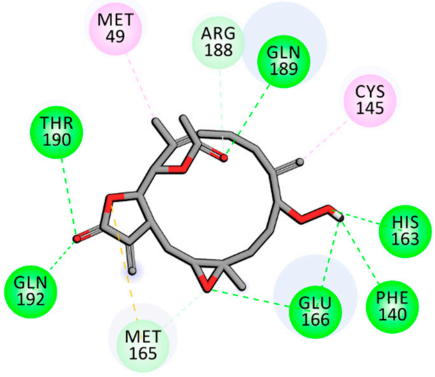

151

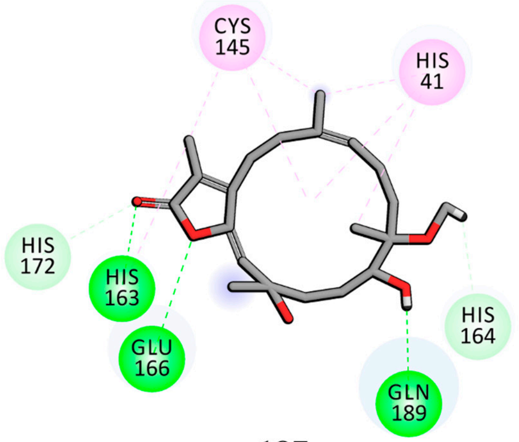

187

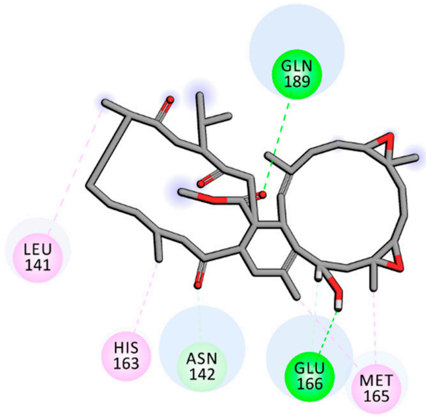

331

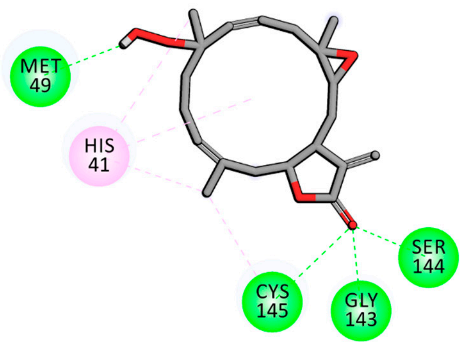

147

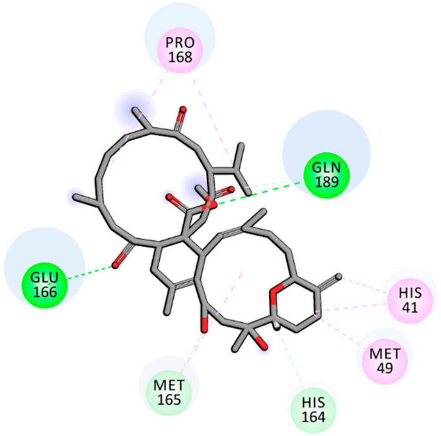

335

Figure S1. *Continued.*

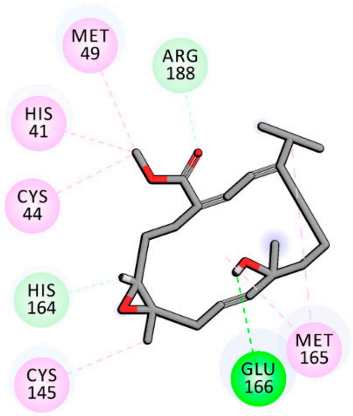

174

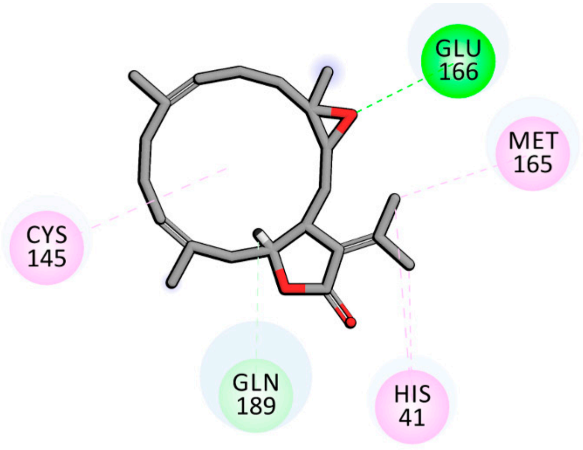

8

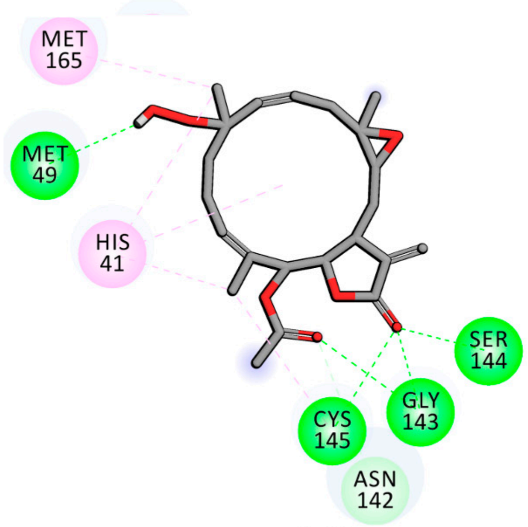

143

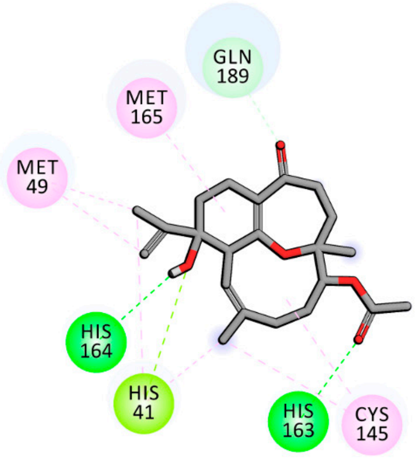

223

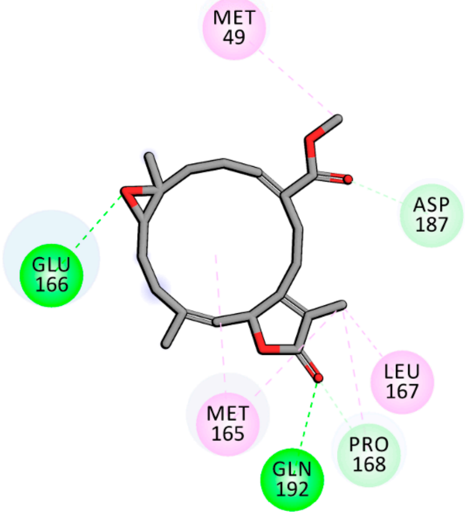

158

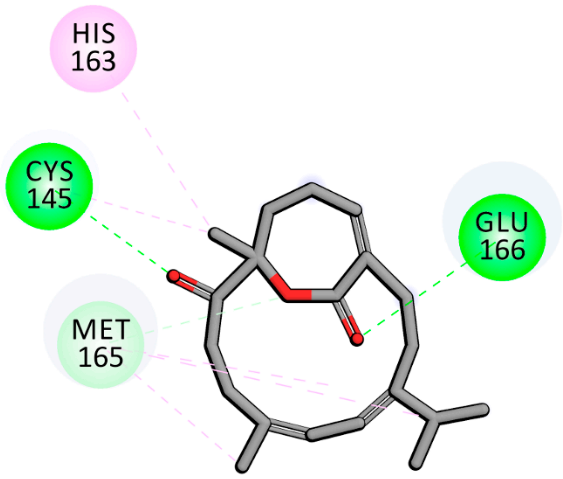

31

Figure S1. *Continued.*

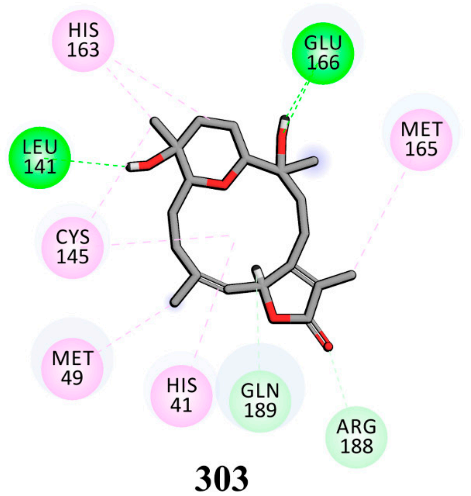

303

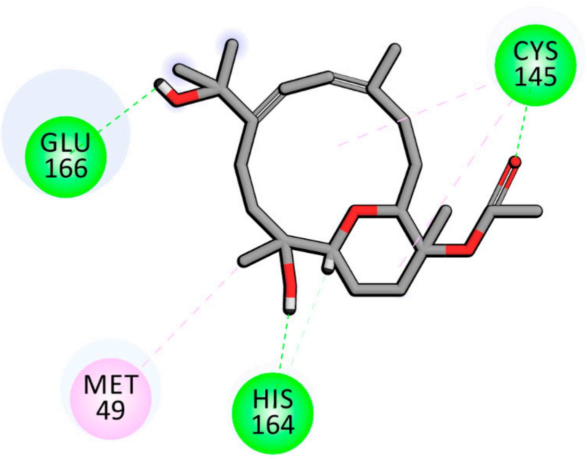

180

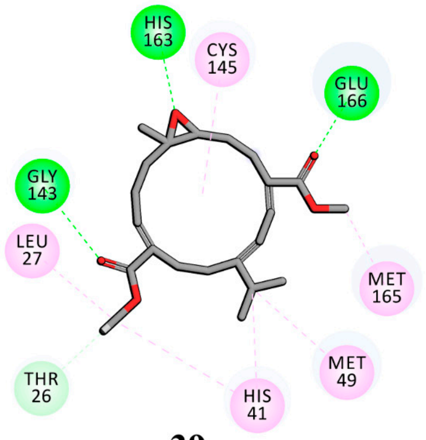

39

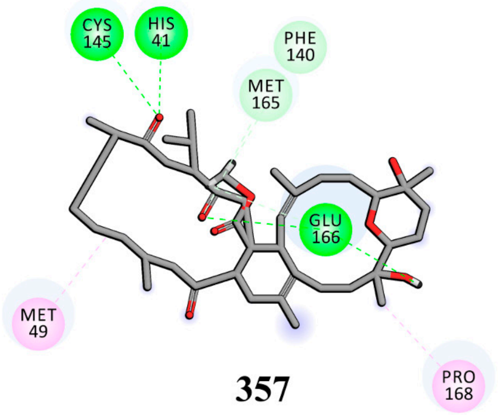

357

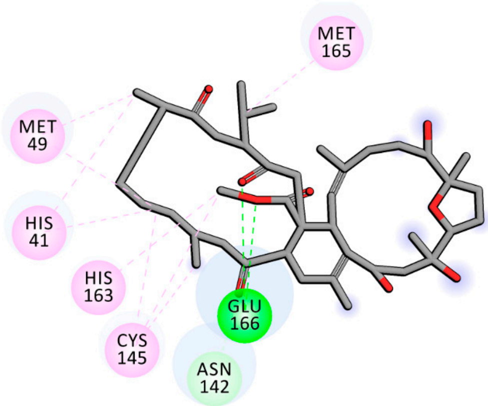

361

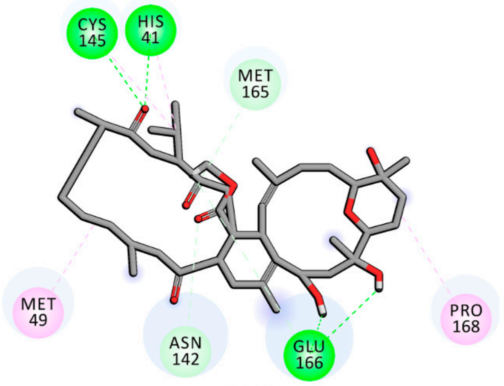

358

Figure S1. *Continued.*

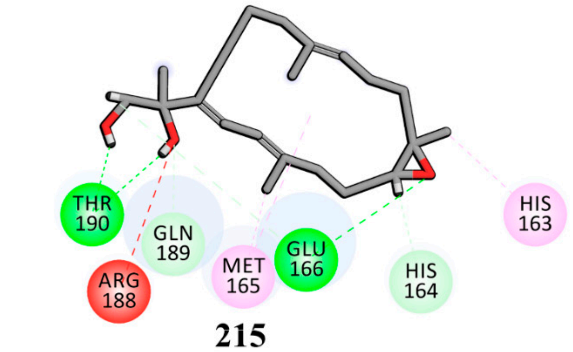

215

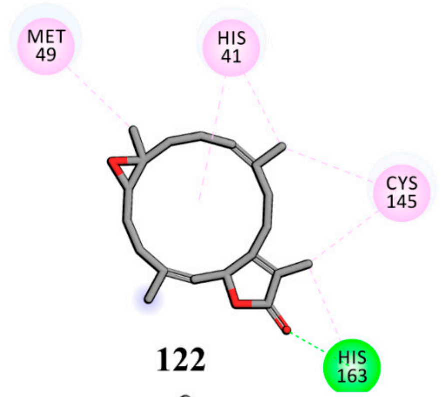

122

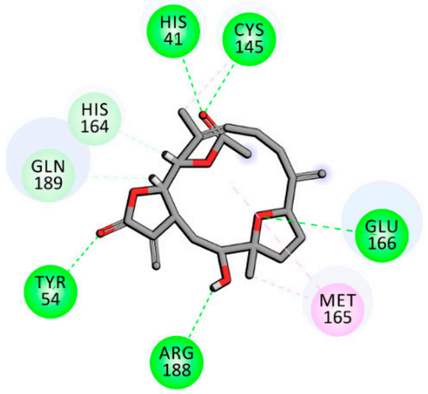

138

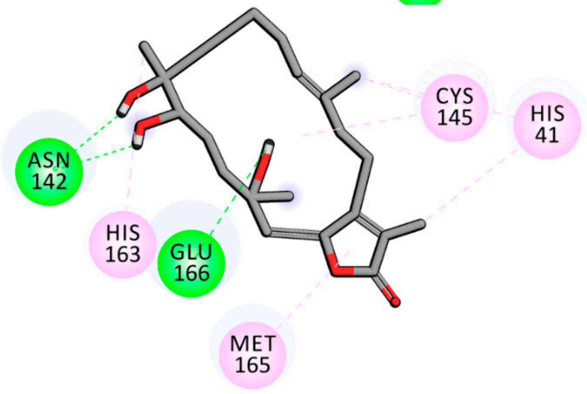

292

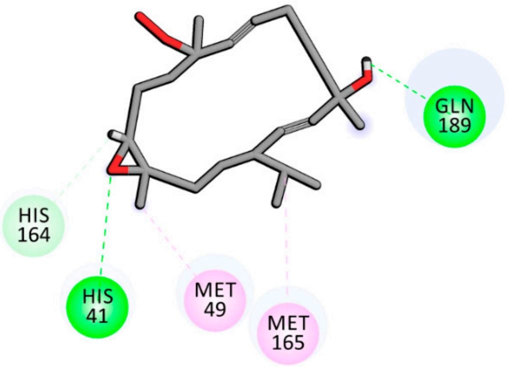

318

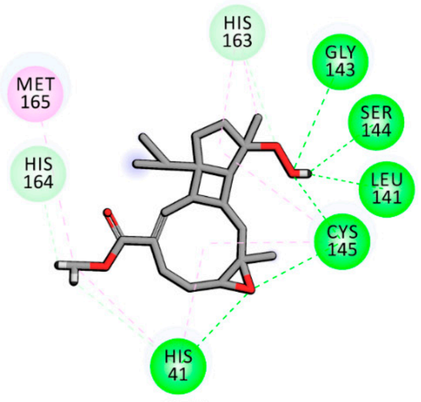

173

Figure S1. *Continued*.

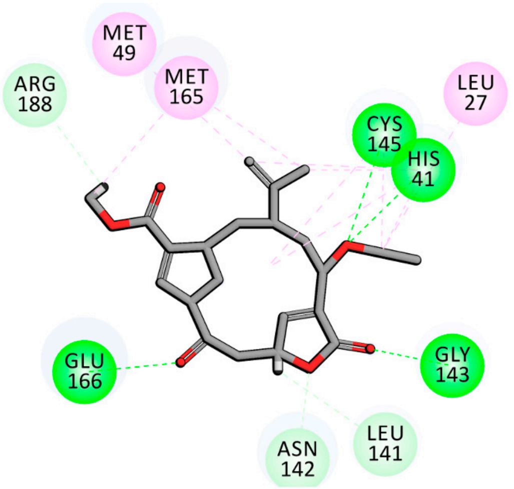

127

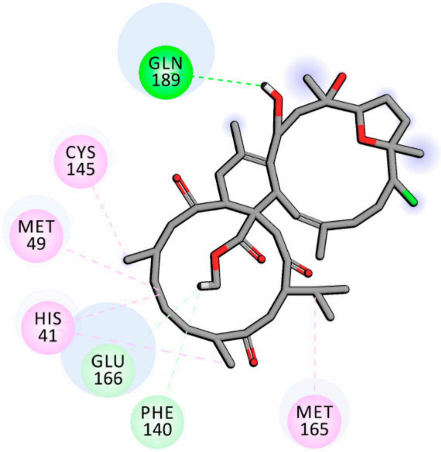

355

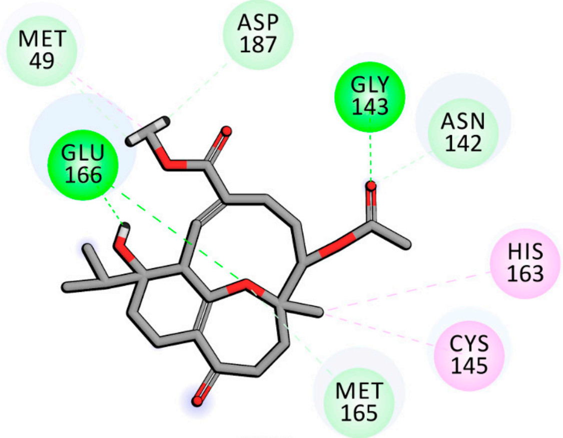

201

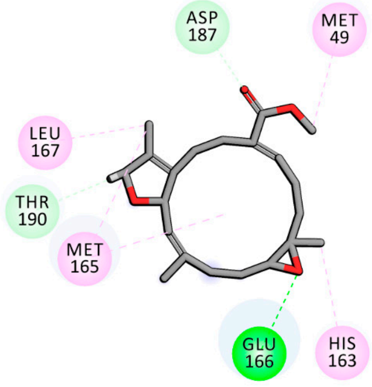

162

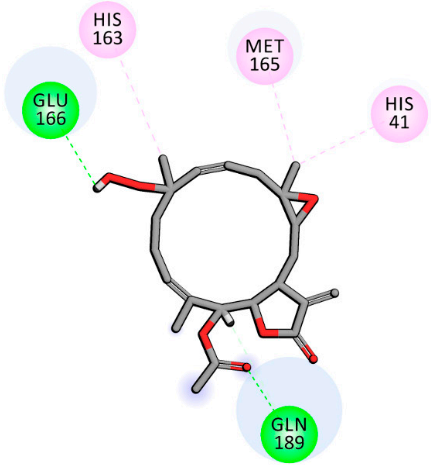

144

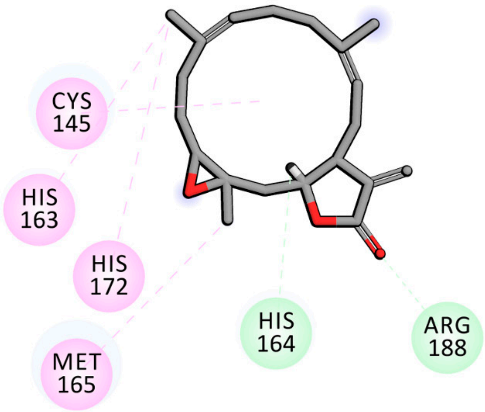

7

Figure S1. *Continued.*

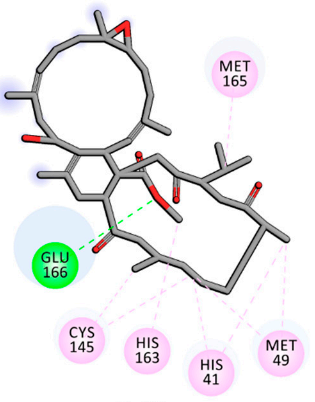

349

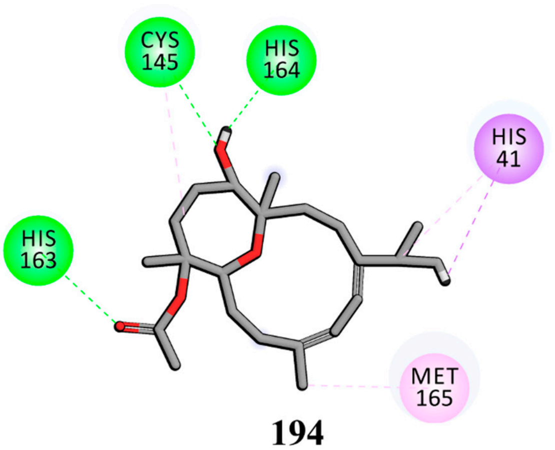

194

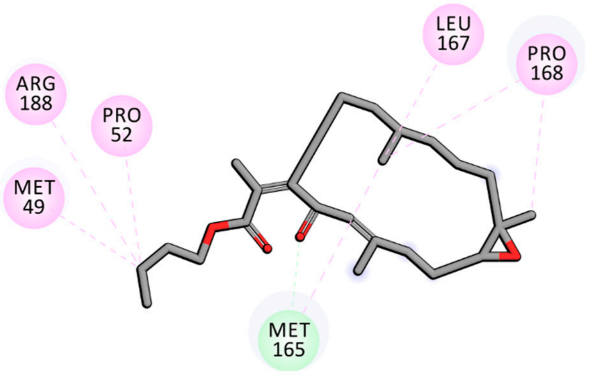

121

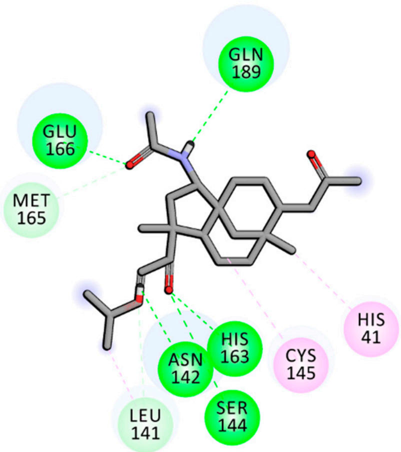

316

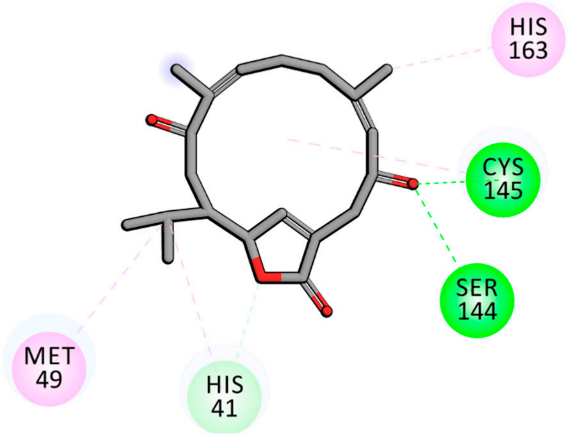

105

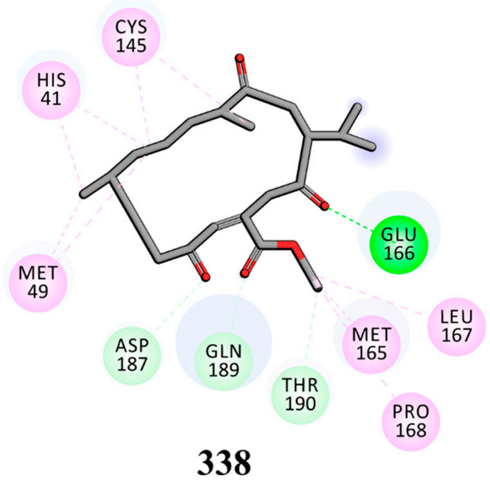

338

Figure S1. *Continued.*

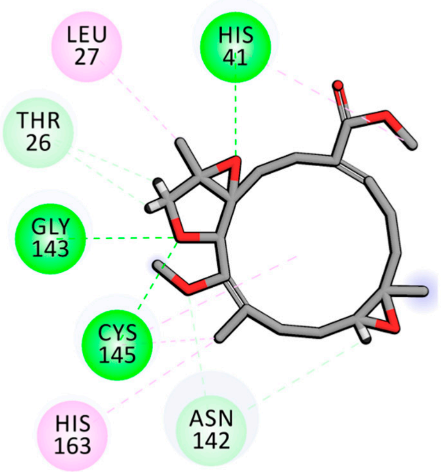

248

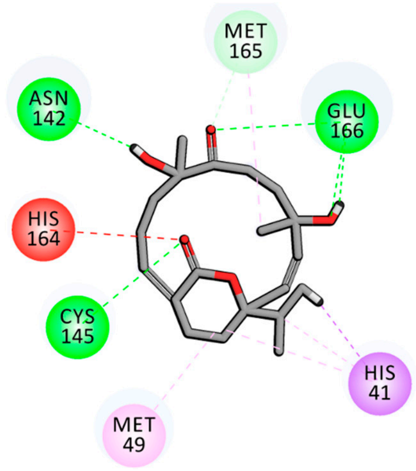

299

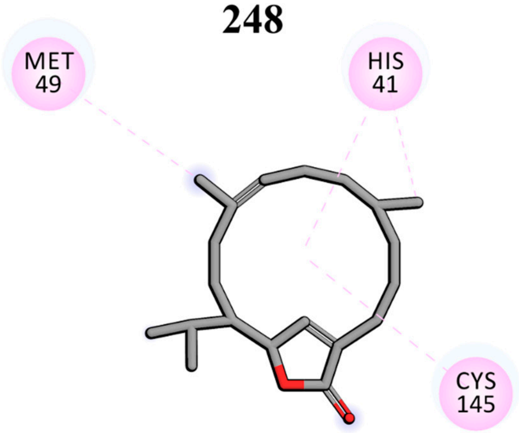

40

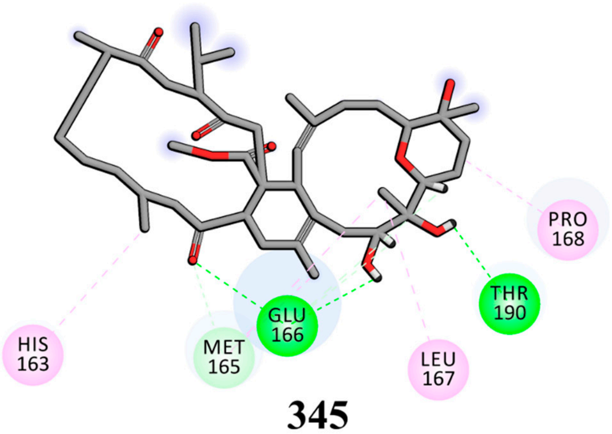

345

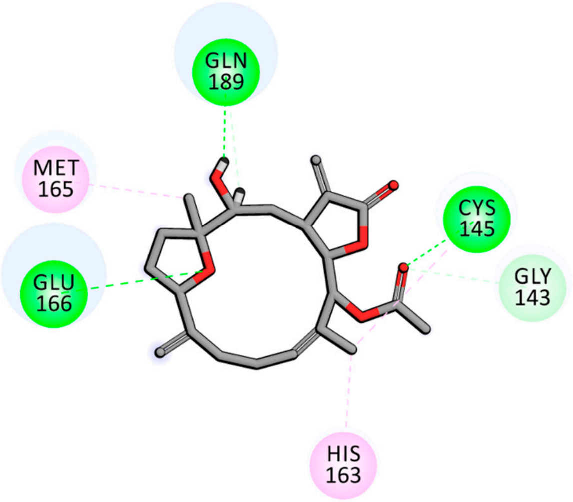

137

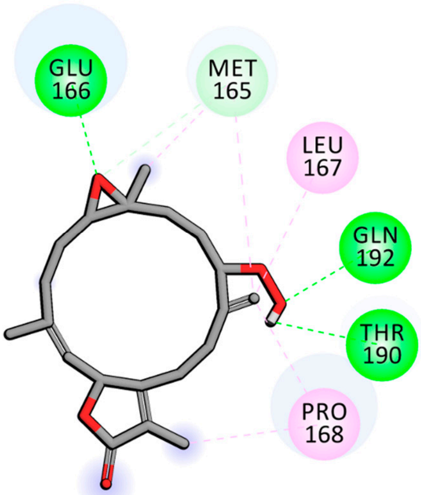

169

Figure S1. *Continued.*

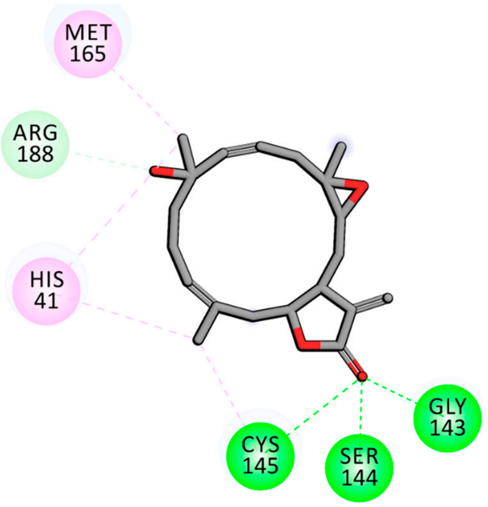

148

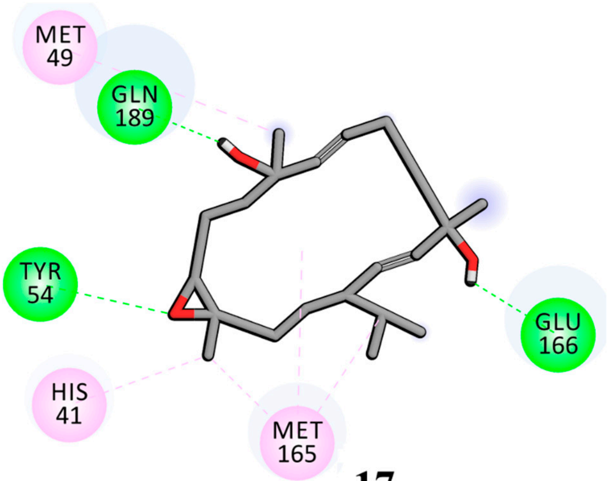

17

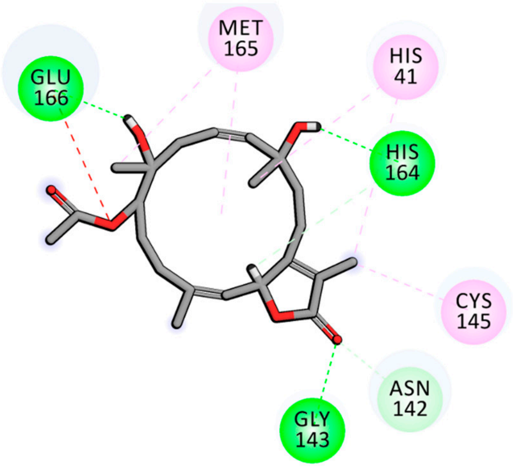

131

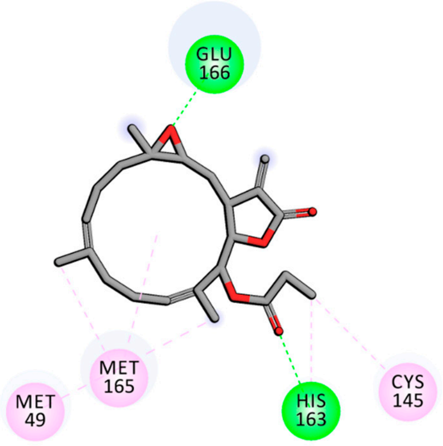

141

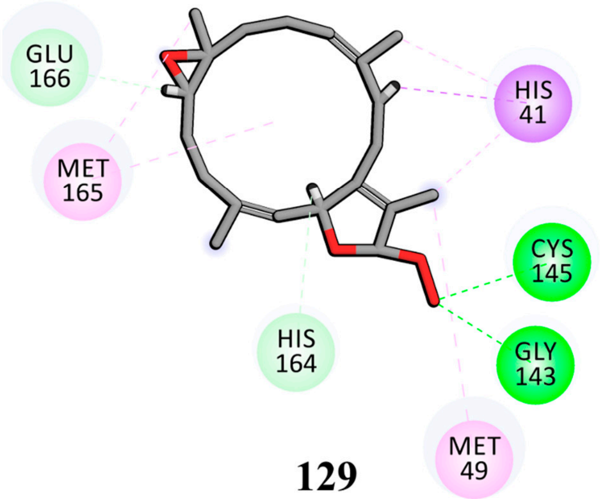

129

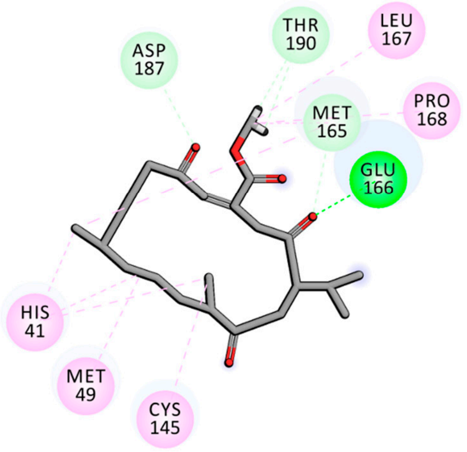

342

Figure S1. *Continued.*

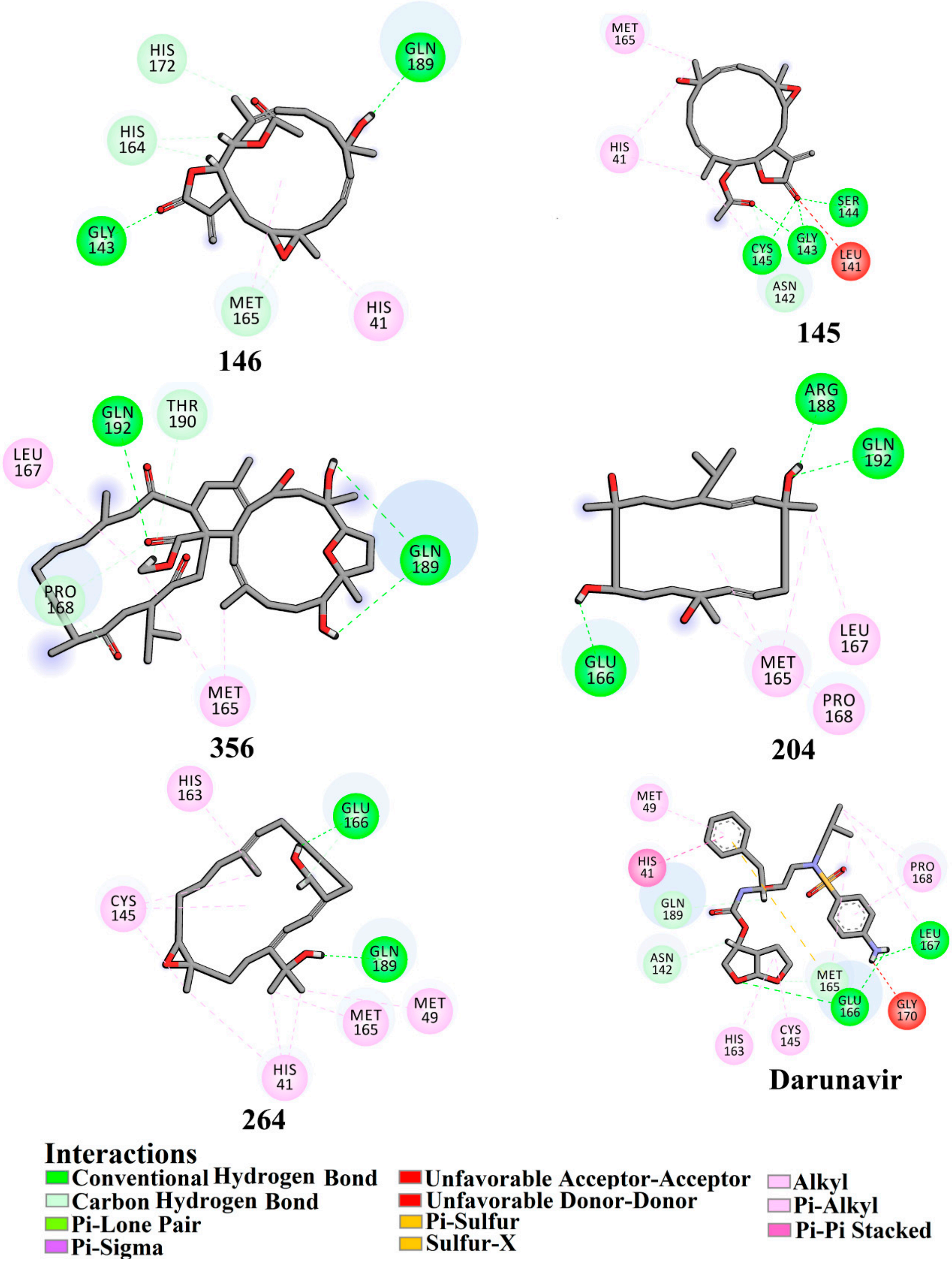

Figure S1. *Continued.*

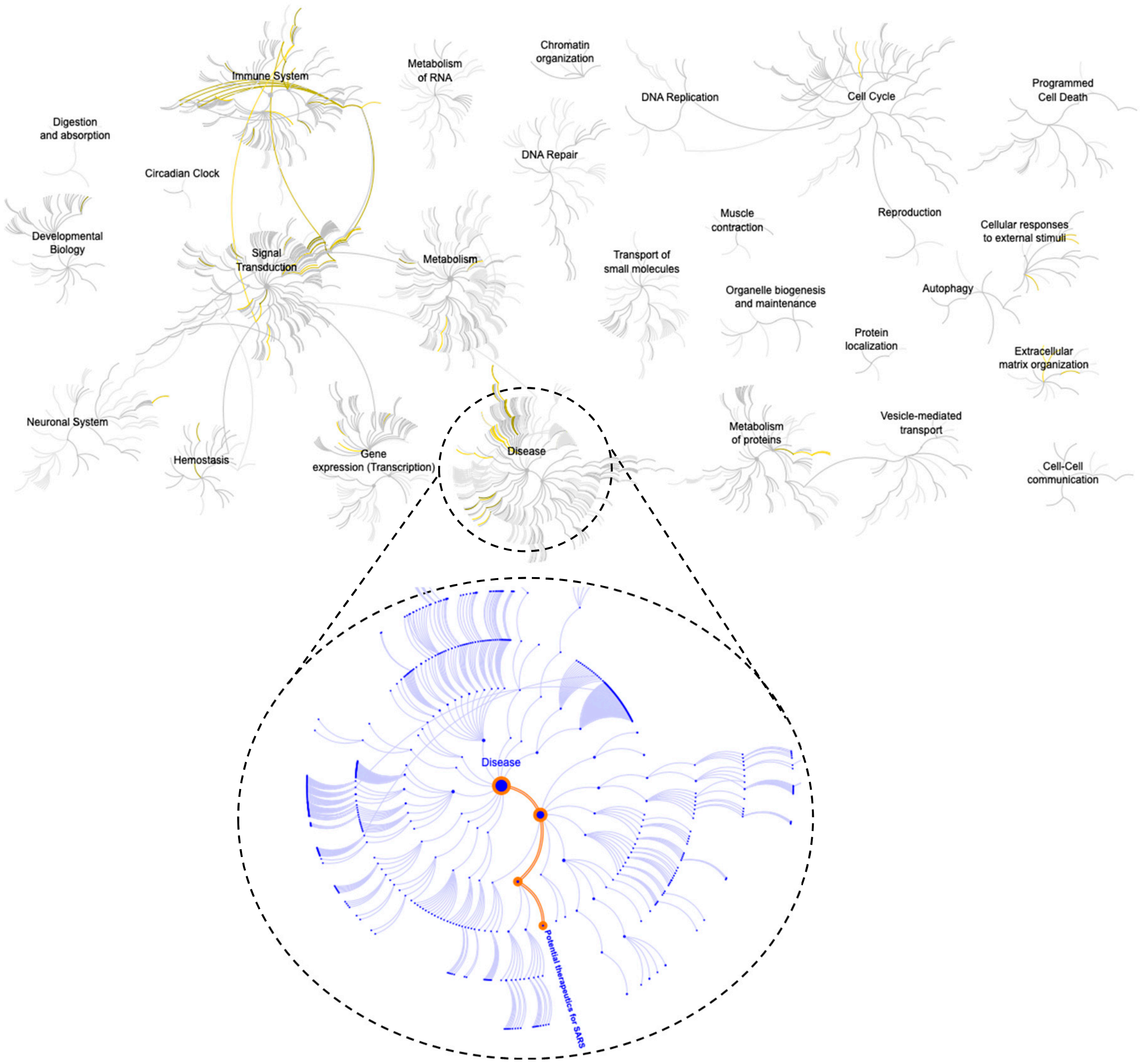

Figure S2. A genome-wide overview of the Reactome Pathway Analysis (RPA) results for the top 20 genes targets stimulated by bislatumlide A (**340**) against SARS-CoV-2. Pathways are organized in a hierarchy form. The light grey color indicates that these pathways are not significantly over-represented. In comparison, light yellow color indicates that these pathways are significantly over-represented by the input gene dataset.

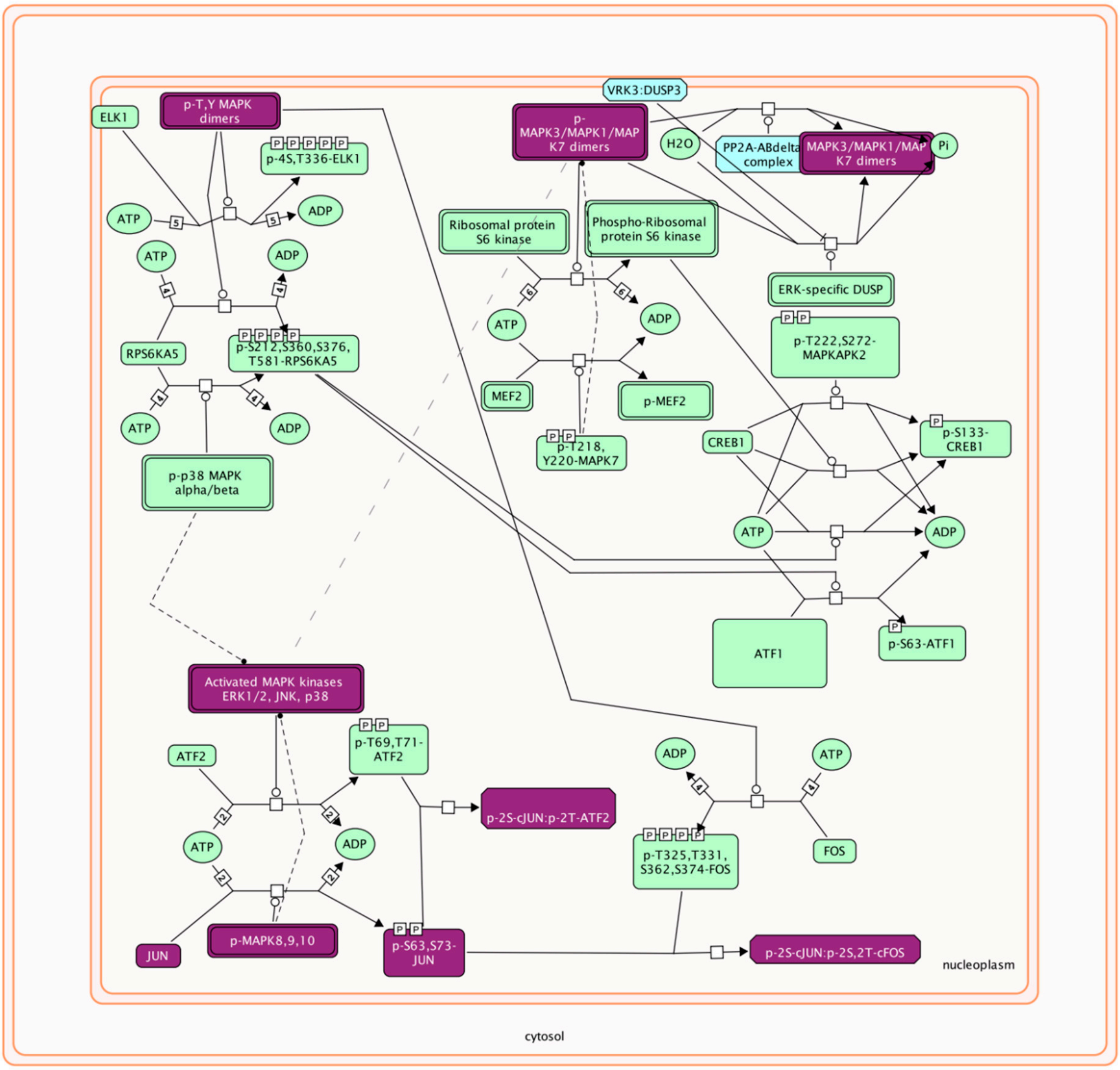

Figure S3. Pathway digram analysis for enzymes/signals of the top 10 genes products/targets (highlited in red) stimulated by bislatumlide A (**340**).

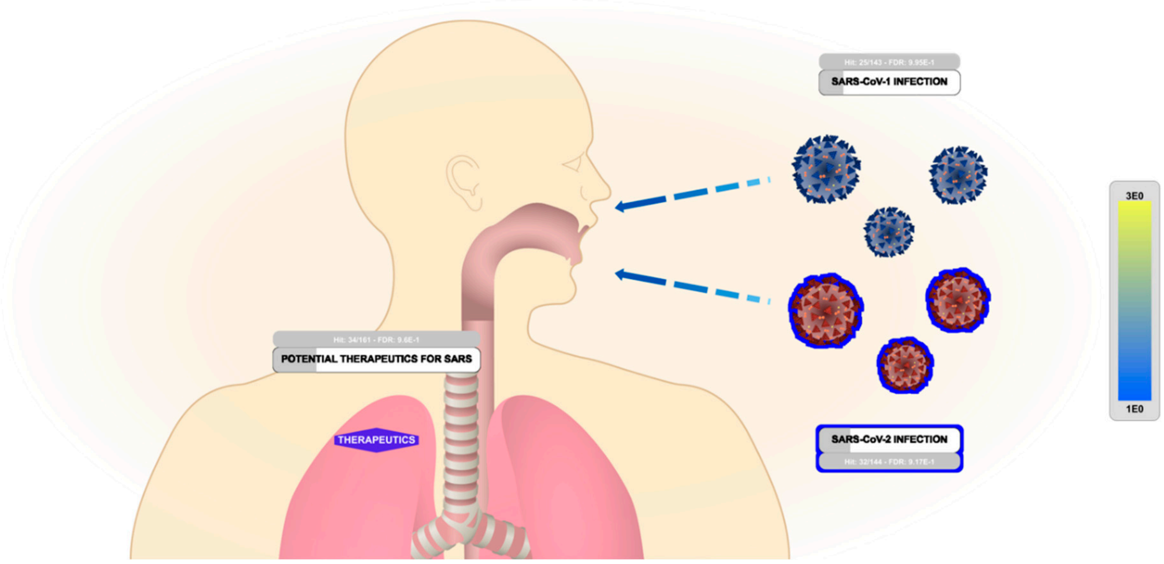

Figure S4. A diagrammatic model shows the potential therapeutic level of bislatumlide A (340) against SARS-CoV-2 viral infection.

Table S1. Estimated docking score (in kcal/mol) for darunavir and the investigated *Sarcophyton* cembranoid diterpenes metabolites towards SARS-CoV-2 main protease (M^pro^)[a].

No.	Compound Name	Soft Coral	Chemical Structure	Docking Score (kcal/mol)
	Darunavir	-----	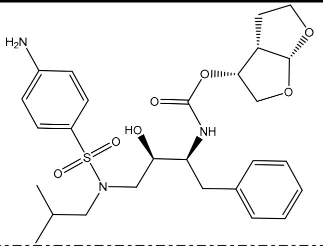	–8.2
1	Sarcophytol T	*Sarcophyton ehrenbergi*	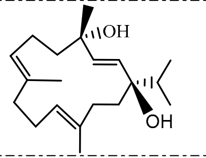	–7.7
2	(1E,3E,7E,11R*12R*)-15-(acetoxymethyl)cembra-11,12-epoxy-1,3,7-triene	*Sarcophyton ehrenbergi*	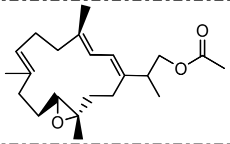	–7.9
3	(11S*,12S*)-15-(acetoxymethyl) cembra-3,4:11,12-diepoxy-1,7-diene	*Sarcophyton ehrenbergi*	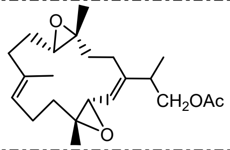	–7.6
4	Isoneocembrene A	*Sarcophyton ehrenbergi*	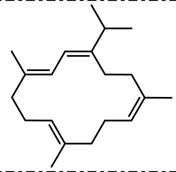	–7.5
5	Neocembrane A	*Sarcophyton ehrenbergi*	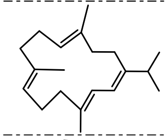	–7.7
6	(2S*,11R*,12R*)-isosarcophytoxide	*Sarcophyton ehrenbergi*	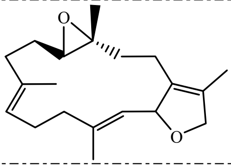	–8.1
7	Crassolide	S. crassocaule	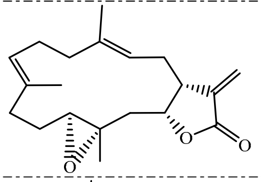	–8.5
8	Sarcocrassolide A	*S. crassocaule*	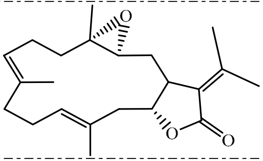	–9.0
9	13-acetoxysarcocrassolide	*S. crassocaule*	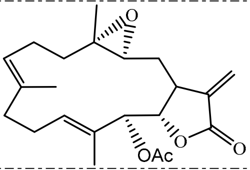	–7.4

10	Denticulatolide	*S. crassocaule* *Sarcophyton sp.*	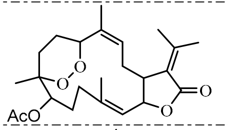	–9.9
11	7,8-epoxy-1(E),3(E),11(E)cembratrien-15-ol	*S. trocheliophorum*	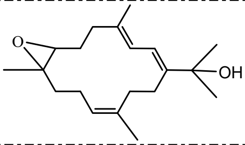	–7.8
12	7,8-epoxy1(E),3(E),11(E)-cembratriene	*S. trocheliophorum*	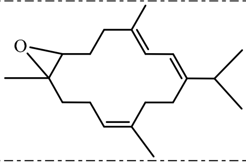	–7.6
13	Sarcophin	*S. trocheliophorum*	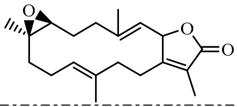	–7.1
14	Acutanol	*S. acutangulum*	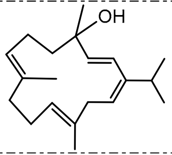	–7.8
15	Sarcophytol A	*S. acutangulum*	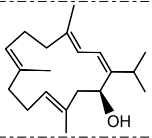	–7.5
16	Sarcophytol A acetate	*S. infundibuliforme*	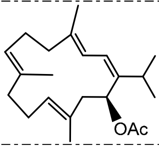	–7.0
17	(1S,2E,4R,6E,8S,11S,12S)-11,12-Epoxy-2,6-cembrane-4,8-diol	*Sarcophyton sp.*	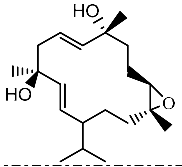	–8.3
18	(1S,2E,4R,6E,8R,11S,12S)-11,12-Epoxy-2,6-cembrane-4,8-diol	*Sarcophyton sp.*	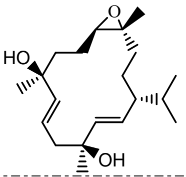	–8.2
19	(1S,2E,4R,7S)-11,12-Epoxy-2,8(19)-cembradiene-4,7-diol	*Sarcophyton sp.*	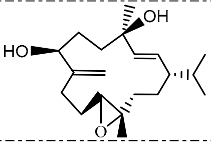	–8.2
20	(1S,2E,4R,7R)-11,12-Epoxy-2,8(19)-cembradiene-4,7-diol	*Sarcophyton sp.*	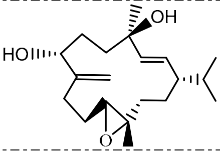	–8.1
21	Sarcophycrassolide A	*S. crassocaule*	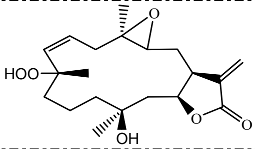	–7.9

22	Sacophyocrassolide B	*S. crassocaule*	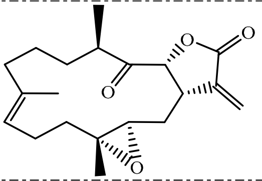	–7.8
23	13-dehydroxysarcoglaucol	*S. cherbonnieri*	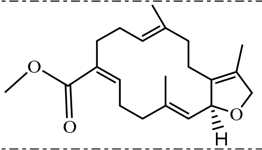	–7.4
24	13-dehydroxysarcoglaucol-16-one	*S. cherbonnieri*	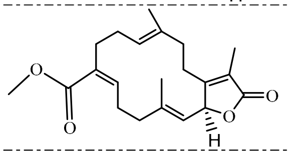	–8.1
25	Sarcoglaucol-16-one	*S. cherbonnieri*<br>*S. ehrenbergi*	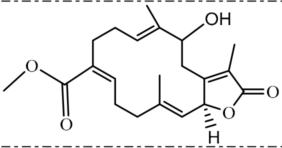	–7.8
26	(3E)-7-hydroxy-4,8,15,15-tetramethyl-1-[(E)-12-methyl-10-oxo-12-pentenyl]-3,8-decadienyl acetate	*S. cherbonnieri*	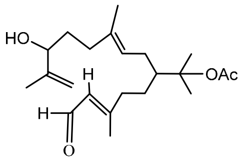	–7.4
27	(3E)-7-hydroxy-4,8,15,15-tetramethyl-1-[(Z)-12-methyl-10-oxo-12-pentenyl]-3,8-decadienyl acetate	*S. cherbonnieri*	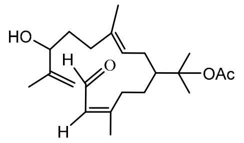	–7.1
28	Sarcoglaucol	*S. cherbonnieri*	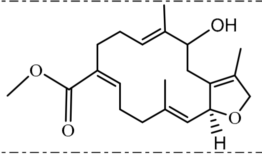	–7.5
29	Decaryiol	*S. cherbonnieri*	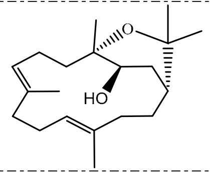	–6.8
30	(4Z,8S,9S,12Z,14E)-9-Hydroxy-1-isopropyl-8,12-dimethyl-oxabicyclo [9.3.2]-hexadeca-4,12,14-trien18-one	*Sarcophyton new sp.*	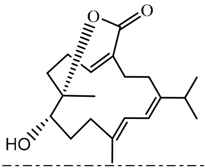	–7.3
31	(4Z,12Z,14E)-sarcophytolide	*Sarcophyton new sp.*	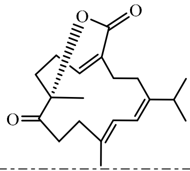	–8.8
32	Sarcophytolide	*Sarcophyton new sp.*	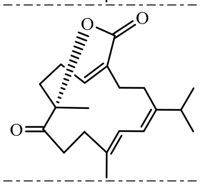	–7.5

33	4Z,8S,9R,12E,14E)-9-Hydroxy-1-isopropyl-8,12-dimethyloxabicyclo[9.3.2]-hexadeca-4,12,14-trien-18-one	*Sarcophyton new sp.*	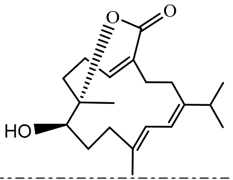	–7.8
34	(4Z,8S,9R,12E,14E)-1-Isopropyl-8,12-dimethyl-18-oxo-oxabicyclo[9.3.2]-hexadeca-4,12,14-trien-2-yl acetate	*Sarcophyton new sp.*	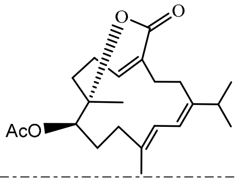	–8.0
35	Sarcophytolol	*S. glaucum*	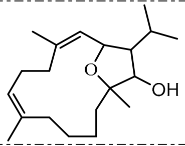	–7.0
36	Sarcophytolide B	*S. glaucum*	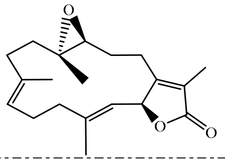	–7.3
37	Sarcophytolide C	*S. glaucum*	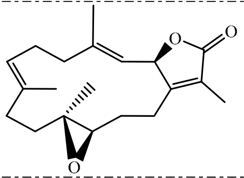	–7.9
38	Sarcophytonolide A	*S. tortuosum*	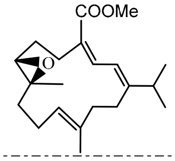	–8.2
39	Sarcophytonolide B	*S. crassocaule*	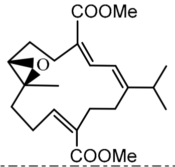	–8.7
40	Sarcophytonolide C	*S. tortuosum*	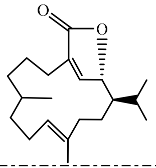	–8.4
41	Sarcophytonolide D	*S. tortuosum*	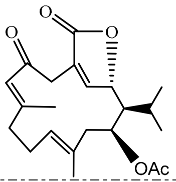	–8.0
42	Sarcophytonolide E	*S. latum*	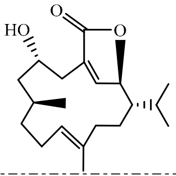	–7.7
43	Sarcophytonolide F	*S. latum*	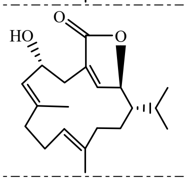	–7.9

44	Sarcophytonolide G	*S. latum*	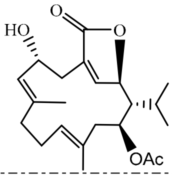	–7.8
45	Sarcophytonolide H	*S. latum*	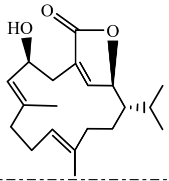	–8.0
46	Sarcophytonolide I	*S. latum*	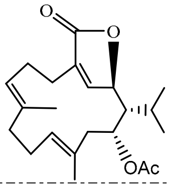	–7.7
47	Sarcophytonolide J	*S. infundibuliforme*	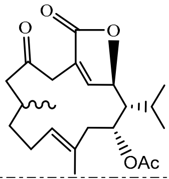	–7.5
48	Sarcophytonolide K	*S. trocheliophorum*	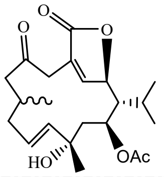	–8.1
49	Sarcophytonolide L	*S. latum*	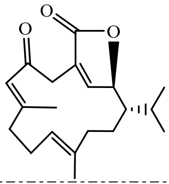	–7.6
50	Sarcophytonolide N	*S. trocheliophorum*	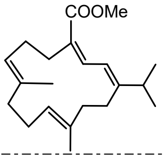	–7.3
51	Sarcophytonolide O	*S. trocheliophorum*	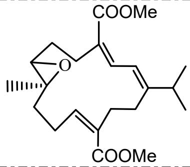	–8.2
52	Sarcophytonolide P	*S. trocheliophorum*	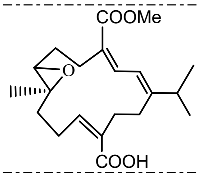	–8.2
53	Sarcophytonolide Q	*S. trocheliophorum*	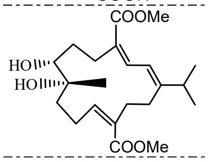	–8.2
54	Sarcophytonolide R	*S. trocheliophorum*	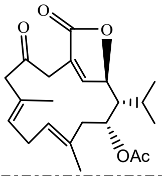	–7.3

55	ketoemblide	*S. trocheliophorum*	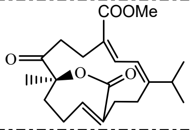	–8.2
56	(E,E,E)-1-isopropenyl-4,8,12-trimethylcyclotetradeca-3,7,11-tiene	*S. trocheliophorum*	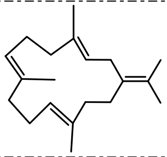	–7.7
57	Sarcophytonolide S	*S. trocheliophorum*	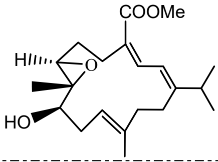	–7.9
58	Sarcophytonolide T	*S. trocheliophorum*	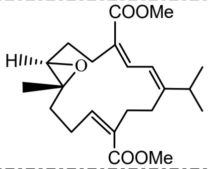	–8.1
59	Sarcophytonolide U	*S. trocheliophorum*	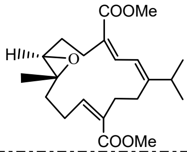	–8.1
60	Sartrolide H	*S. trocheliophorum*	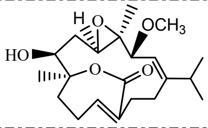	–7.4
61	Sartrolide I	*S. trocheliophorum*	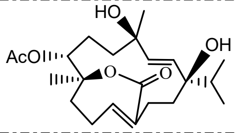	–7.3
62	Sartrolide J	*S. trocheliophorum*	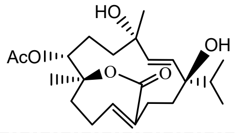	–7.5
63	Sartrolide A	*S. trocheliophorum*	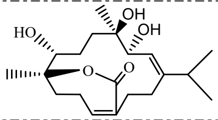	–7.0
64	Sartrolide B	*S. trocheliophorum*	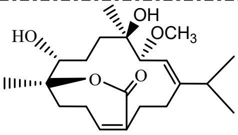	–7.3
65	Sartrolide C	*S. trocheliophorum*	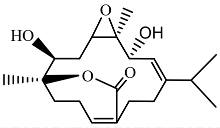	–6.7
66	Sartrolide D	*S. trocheliophorum*	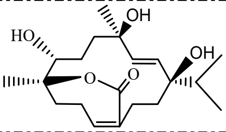	–7.9
67	Sartrolide E	*S. trocheliophorum*	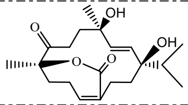	–7.5

68	Sartrolide F	*S. trocheliophorum*	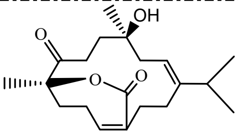	–7.6
69	Sartrolide G	*S. trocheliophorum*	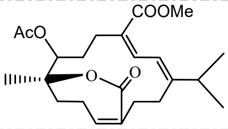	–8.2
70	Bissartrolide dimer	*S. trocheliophorum*	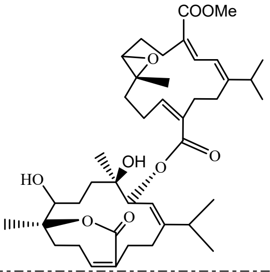	–8.1
71	Yalongene A	*S. trocheliophorum*	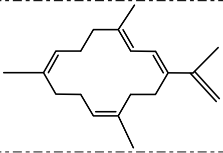	–8.0
72	Yalongene A	*S. trocheliophorum*	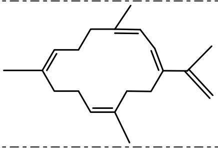	–6.5
73	Trochelioid A	*S. trocheliophorum*	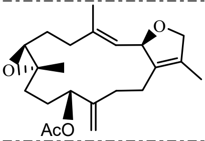	–7.8
74	Trochelioid B	*S. trocheliophorum*	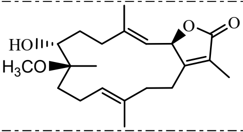	–8.1
75	16-oxosarcophytonin E	*S. trocheliophorum*	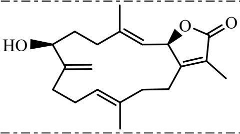	–7.5
76	Sarcrassin A	*S. crassocaule*	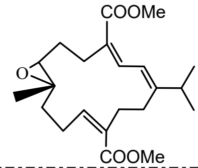	–8.2
77	Sarcrassin B	*S. crassocaule*	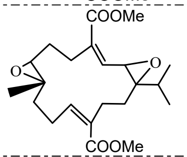	–6.4
78	Sarcrassin C	*S. crassocaule*	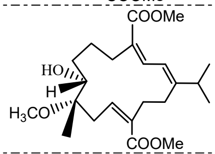	–7.8
79	Sarcrassin D	*S. crassocaule*	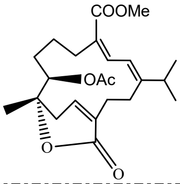	–8.2

80	Sarcrassin E	*S. crassocaule*	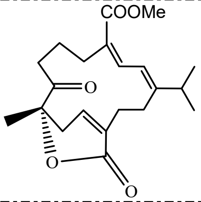	–8.1
81	Emblide	*S. crassocaule*<br>*S. tortuosum*	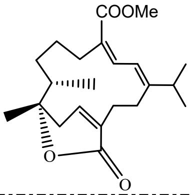	–8.2
82	Crassocolide A	*S. crassocaule*	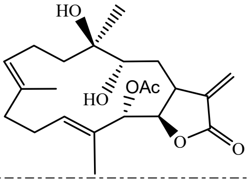	–8.1
83	Crassocolide B	*S. crassocaule*	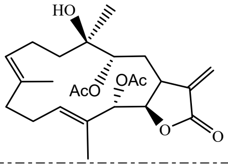	–7.4
84	Crassocolide C	*S. crassocaule*	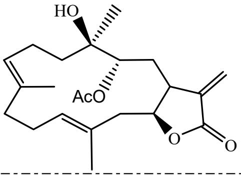	–6.5
85	Crassocolide D	*S. crassocaule*	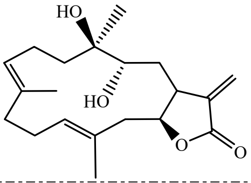	–7.7
86	Crassocolide E	*S. crassocaule*	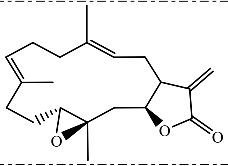	–8.2
87	Crassocolide F	*S. crassocaule*	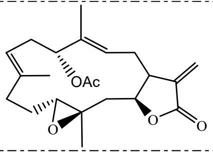	–8.1
88	Lobophytolide	*S. crassocaule*	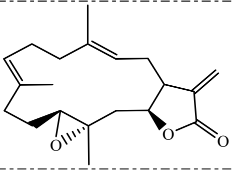	–7.5
89	Crassocolide G	*S. crassocaule*	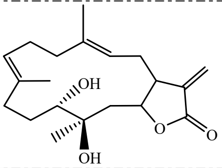	–6.3

90	Crassocolide H	*S. crassocaule*	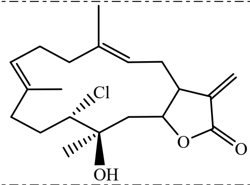	–7.0
91	Crassocolide I	*S. crassocaule*	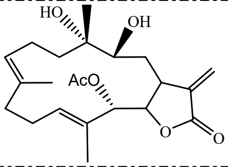	–7.0
92	Crassocolide J	*S. crassocaule*	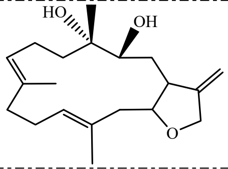	–7.7
93	Crassocolide K	*S. crassocaule*	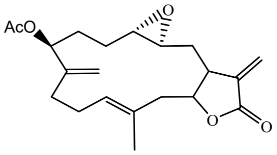	–8.1
94	Crassocolide L	*S. crassocaule*	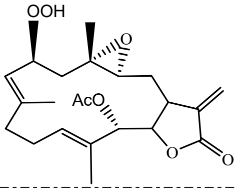	–8.0
95	Crassocolide M	*S. crassocaule*	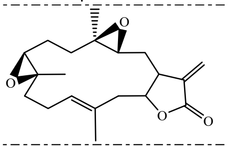	–7.7
96	Crassocolide N	*S. crassocaule*	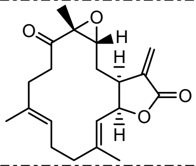	–7.6
97	Crassocolide O	*S. crassocaule*	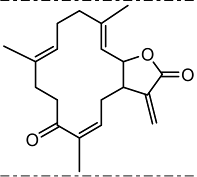	–8.1
98	Crassocolide P	*S. crassocaule*	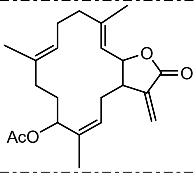	–8.1
99	Flexusine A	*S. flexuosum*	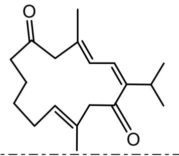	–7.4
100	Flexusine B	*S. flexuosum*	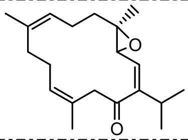	–8.1

101	Epimukulol	*S. flexuosum*	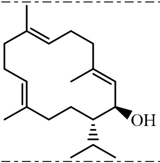	–7.4
102	Sarcostolide A	*S. stolidotum*	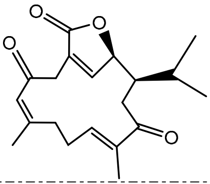	–8.2
103	Sarcostolide B	*S. stolidotum*	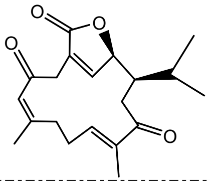	–8.2
104	Sarcostolide C	*S. stolidotum*	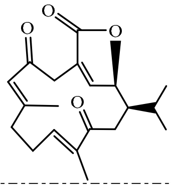	–8.0
105	Sacostolide D	*S. stolidotum*	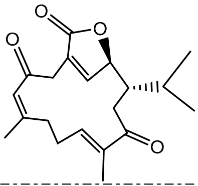	–8.4
106	Sarcostolide E	*S. stolidotum*	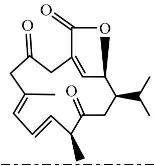	–7.6
107	Sarcostolide F	*S. stolidotum*	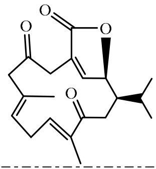	–7.8
108	Sacostolide G	*S. stolidotum*	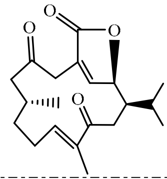	–7.4
109	Isosarcophin	*S. stolidotum*	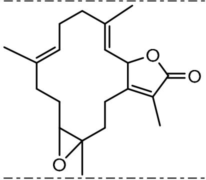	–6.9
110	(-)-7β-hydroxy-8α-methoxy-deepoxy-sarcophytoxide	*S. mililatensis*	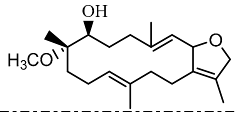	–6.9
111	(+)-7β,8β-Dihydroxy-deepoxy-sarcophytoxide	*S. mililatensis*	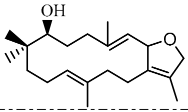	–6.6
112	(-)-17-hydroxysarcophytonin A	*S. mililatensis*	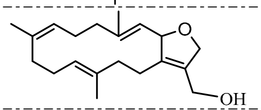	–7.3

113	Sarcophytol V	*S. mililatensis*	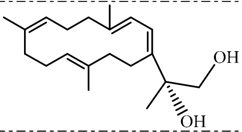	–6.8
114	Sarcophytoxide	*S. mililatensis*<br>*S. glaucum*<br>*Sarcophyton sp.*<br>*S. trocheliophorum*	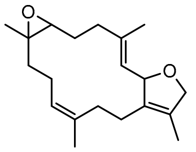	–6.9
115	17-hydroxysarcophytoxide	*S. glaucum*	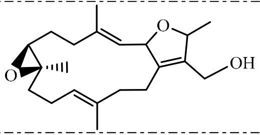	–6.7
116	7β-acetoxy-8α-hydroxydeepoxysarcophine	*S. glaucum*	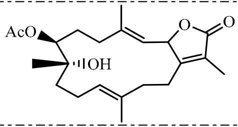	–7.6
117	7α,8βDihydroxydeepoxysarcophine	*S. elegans*<br>*S. auritum*<br>*S. glaucum*	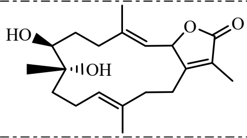	–7.2
118	Sarcophytonin A	*S. mililatensis*	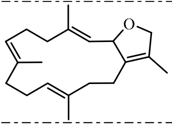	–6.8
119	(–)-β-Elemene	*Sarcophyton sp.*	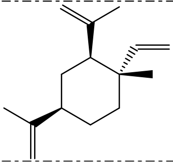	–4.3
120	(7R,8S)-dihydroxydeepoxy-ent-sarcophine	*S. glaucum*	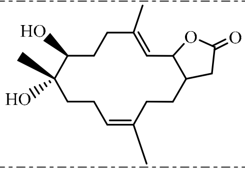	–7.5
121	Secosarcophinolide	*S. glaucum*	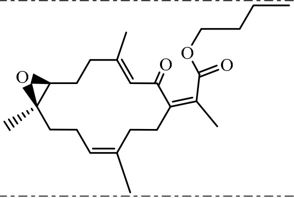	–8.4
122	Ent-sarcophine	*S. glaucum*	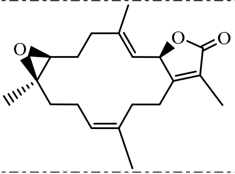	–8.6
123	(7S,8R)-dihydroxydeepoxysarcophine	*S. glaucum*	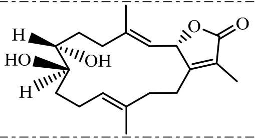	–7.2
124	5-epi-sinuleptolide	*Sarcophyton sp.*	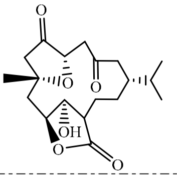	–7.3

125	Lobohedleolide	*Sarcophyton sp.*	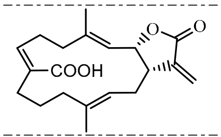	–8.2
126	(7Z)-lobohedleolide	*Sarcophyton sp.*	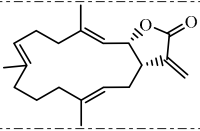	–8.2
127	Sarcofuranocembrenolide A	*Sarcophyton sp.*	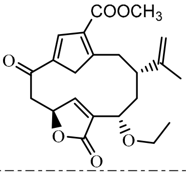	–8.5
128	Sarcofuranocembrenolide B	*Sarcophyton sp.*	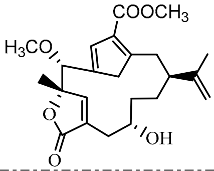	–7.7
129	Sarcophytonins F	*Sarcophyton sp.*	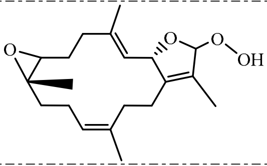	–8.3
130	Sarcophytonin G	*Sarcophyton sp.*	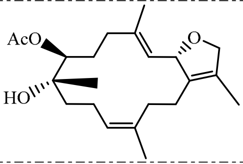	–6.9
131	7-Acetyl-8-epi- sinumaximol G	*S. ehrenbergi*	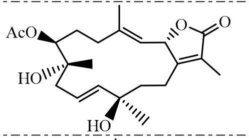	–8.3
132	8-epi-sinumaximol G	*Sarcophyton sp.*	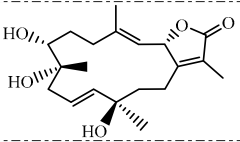	–7.9
133	12-acetyl-7, 12-episinumaximol G	*Sarcophyton sp.*	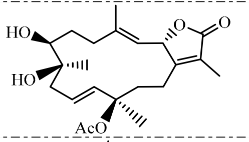	–8.0
134	12-hydroxysarcoph-10-ene	*S. ehrenbergi*	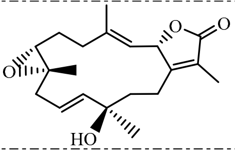	–7.7
135	8-hydroxy-epi-sarcophinone	*S. ehrenbergi*	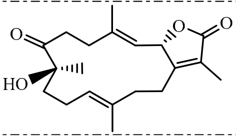	–7.3
136	Sinumaximol G	*S. ehrenbergi*	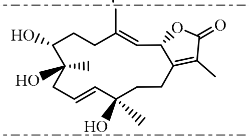	–7.4
137	Sarcocrassocolide A	*S. crassocaule*	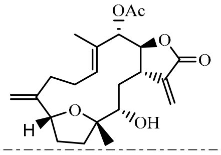	–8.3

138	Sarcocrassocolide B	*S. crassocaule*	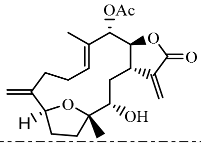	–8.6
139	Sarcocrassocolide C	*S. crassocaule*	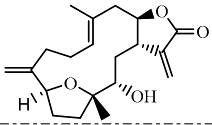	–8.2
140	Sarcocrassocolide D	*S. crassocaule*	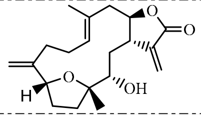	–8.1
141	Sarcocrassocolide E	*S. crassocaule*	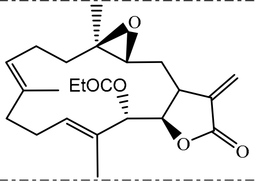	–8.3
142	Sinularolide	*S. crassocaule*	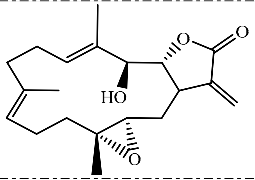	–7.9
143	Sarcocrassocolide F	*S. crassocaule*	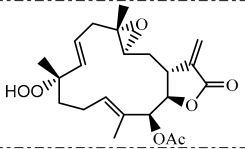	–9.0
144	Sarcocrassocolide G	*S. crassocaule*	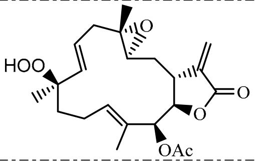	–8.5
145	Sarcocrassocolide H	*S. crassocaule*	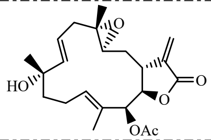	–8.3
146	Sarcocrassocolide I	*S. crassocaule*	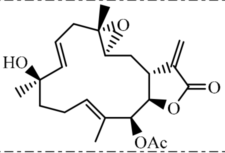	–8.3
147	Sarcocrassocolide J	*S. crassocaule*	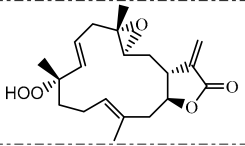	–9.1
148	Sarcocrassocolide K	*S. crassocaule*	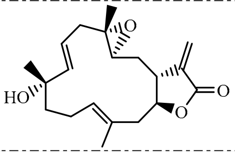	–8.3
149	Sarcocrassocolide L	*S. crassocaule*	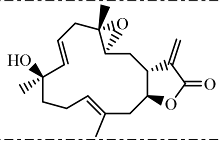	–8.1
150	Sarcocrassocolide M	*S. crassocaule*	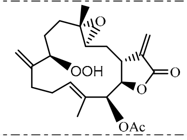	–7.5

151	Sarcocrassocolide N	*S. crassocaule*	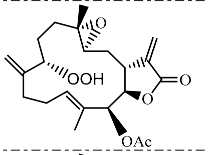	–9.4
152	Sarcocrassocolide O	*S. crassocaule*	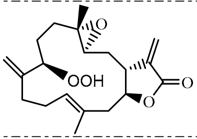	–7.5
153	Sarcocrassocolide P	*S. crassocaule*	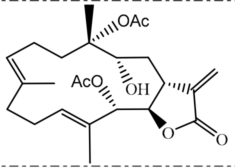	–7.1
154	Sarcocrassocolide Q	*S. crassocaule*	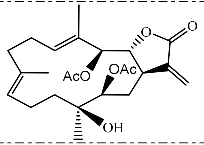	–7.8
155	Sarcocrassocolide R	*S. crassocaule*	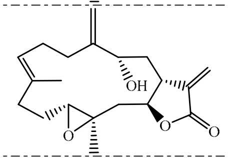	–7.3
156	(+)-7,8-epoxy-7,8-dihydrocembrene C	*S. ehrenbergi*	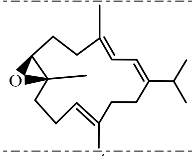	–8.0
157	(+)-12-carboxy-11Z-sarcophytoxide	*S. ehrenbergi*	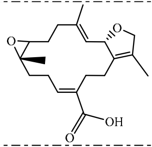	–8.0
158	(+)-12-methoxycarbonyl-11Z-sarcophine	*S. ehrenbergi*	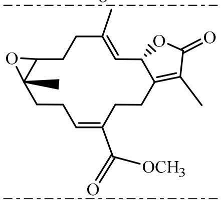	–8.9
159	Ehrenberoxide A	*S. ehrenbergi*	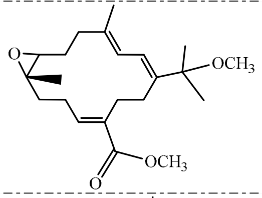	–8.1
160	Ehrenberoxide B	*S. ehrenbergi*	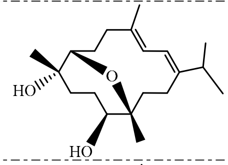	–7.0
161	Ehrenberoxide C	*S. ehrenbergi*	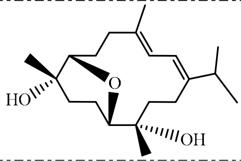	–7.2

162	Lobophynin C	*S. ehrenbergi*	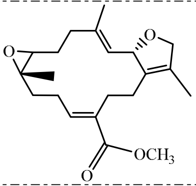	–8.5
163	Cembrene C	*S. trocheliophorum*	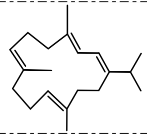	–7.5
164	Sarcophytol B	*Sarcophyton sp.*	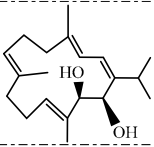	–7.9
165	Sarcophytol E	*S. infundibuliforme*	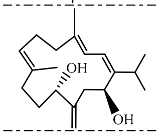	–7.7
166	Sarcophytol H	*S. infundibuliforme*	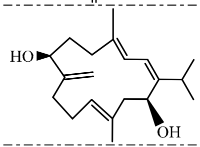	–7.5
167	(-)-marasol	*S. infundibuliforme* *S. glaucum*	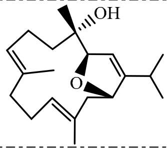	–7.2
168	2R,7R,8R-dihydroxydeepoxysarcophine	*S. glaucum*	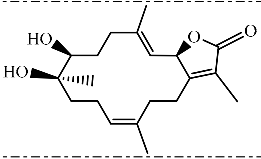	–7.8
169	11(S)-hydroperoxylsarcoph-12(20)-ene	*S. glaucum*	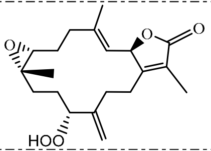	–8.3
170	12(S)-hydroperoxylsarcoph-10-ene	*S. glaucum*	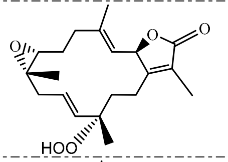	–8.2
171	8-epi-sarcophinone	*S. glaucum*	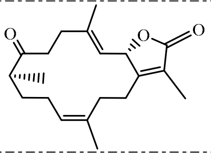	–6.7
172	Methyl sarcotroate A	*S. trocheliophorum*	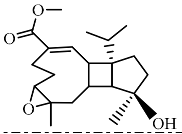	–8.1
173	Methyl sarcotroate B	*S. trocheliophorum*	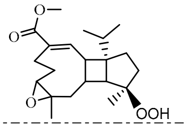	–8.5

174	Sarcophytonolide M	*S. trocheliophorum*	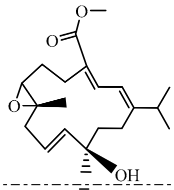	–9.0
175	(1S,2E,4R,6E,8S,11R,12S)-8,11-epoxy-4,12-epoxy- 2,6-cembradiene	*S. glaucum*	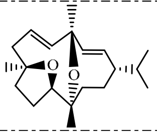	–7.6
176	(1S,2E,4R,6E,8R,11S,12R)-8,12-epoxy-2,6-cembradiene-4,11-diol	*S. glaucum*	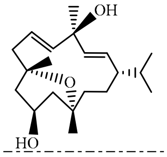	–7.7
177	(1S,4R,13S)-cembra-2E,7E,11E-trien-4,13-diol	*S. glaucum*	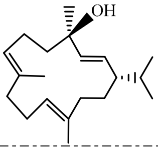	–7.8
178	(+)-12-ethoxycarbonyl-11Z-sarcophine	*S. ehrenbergi*	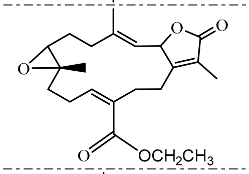	–7.9
179	Ehrenbergol A	*S. ehrenbergi*	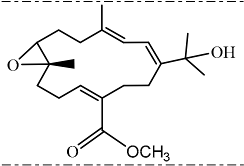	–8.0
180	Ehrenbergol B	*S. ehrenbergi*	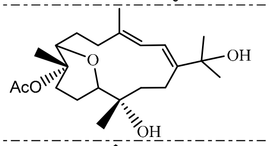	–8.8
181	Sarcophyolide B	*S. elegans*	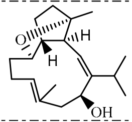	–7.2
182	Sarcophyolide C	*S. elegans*	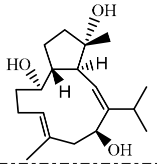	–7.8
183	Sarcophyolide D	*S. elegans*	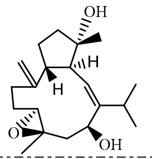	–6.9
184	Sarcophyolide E	*S. elegans*	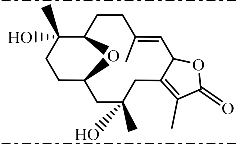	–7.9
185	Sarcophytol L	*S. elegans*	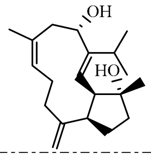	–7.2

186	13α-hydroxysarcophytol L	*S. elegans*	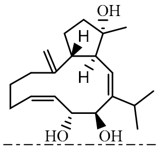	–7.3
187	Sarcophyolide A	*S. elegans*	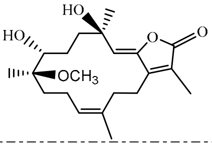	–9.3
188	Sarcophinone	*S. elegans*	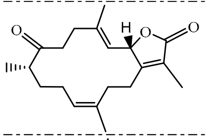	–6.7
189	7α-hydroxy-Δ^{8}(19)-deepoxysarcophine	*S. elegans*	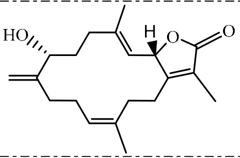	–7.1
190	4β-hydroxy-Δ^{2}(3)-sarcophine	*S. elegans*	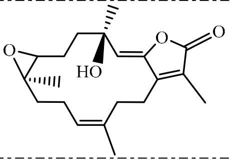	–7.6
191	1,15β-epoxy-2-epi-16-deoxysarcophine	*S. elegans*	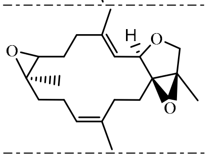	–7.6
192	Sarcophytol Q	*S. elegans*	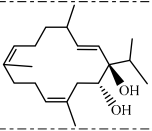	–7.8
193	Lobocrasol	*S. elegans*	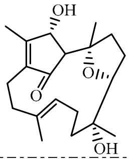	–6.6
194	Acetyl ehrenberoxide B	*S. ehrenbergi*	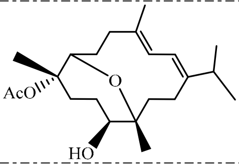	–8.4
195	Ehrenbergol C	*S. ehrenbergi*	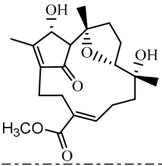	–7.4
196	Sarcophytol W	*S. infundibuliforme*	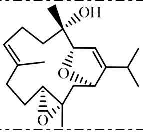	–7.5
197	(2E,7E)-4,11-dihydroxy-1,12-oxidocembra-2,7-dien	*S. glaucum*	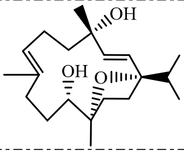	–7.5

198	(+)-11,12-epoxy-11,12-dihydrocembrene-C	*Sarcophyton sp.*	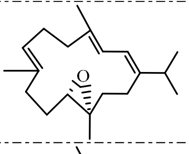	–7.3
199	(+)-11,12-epoxysarcophytol A	*Sarcophyton sp.*	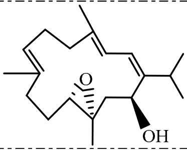	–7.2
200	Sarcolactone A	*Sarcophyton sp.*	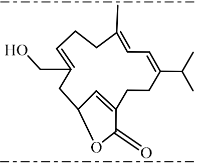	–7.9
201	Tortuosene A	*S. tortuosum*	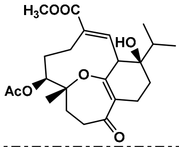	–8.5
202	Tortuosene B	*S. tortuosum*	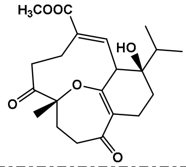	–8.0
203	2-epi-sarcophine	*S. auritum*	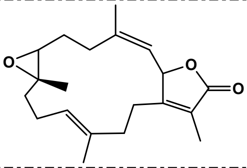	–7.6
204	(1R,2E,4S,6E,8R,11R,12R)-2,6-cembradiene-4,8,11,12-tetrol	*S. auritum*	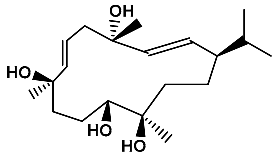	–8.3
205	Sarcophyton A	*Sarcophyton sp.*	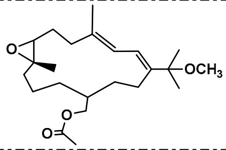	–8.2
206	Sarcophyton B	*Sarcophyton sp.*	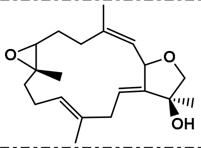	–7.8
207	Sarcophyton C	*Sarcophyton sp.*	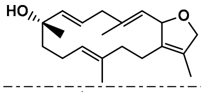	–7.2
208	Sarcophyton D	*Sarcophyton sp.*	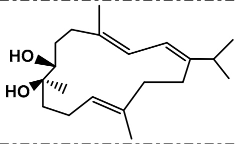	–7.2
209	2-[(E,E,E)-7`,80-epoxy-4`,8`,12`-trimethylcyclotetradeca-1`,3`,11-trienyl]propan-2-ol	*Sarcophyton sp.*	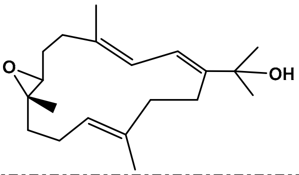	–8.2

210	(1E,3E,7R*,8R*,11E)-1-(2-methoxy-propan-2-yl)-4,8,12-trimethyloxabicyclo[12.1.0]-pentadeca-1,3,11-triene	*Sarcophyton sp.*	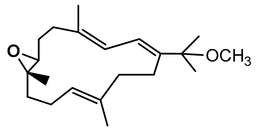	–7.5
211	Crassumol C	*Sarcophyton sp.*	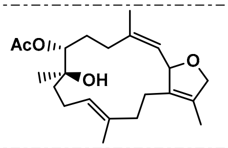	–7.4
212	Laevigatol A	*Sarcophyton sp.*	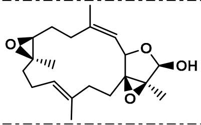	–7.0
213	Sarcotrocheliol acetate	*S. glaucum* *S.trocheliophorum*	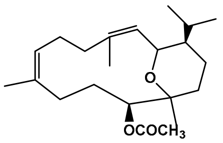	–7.3
214	Sarcotrocheliol	*S. trocheliophorum*	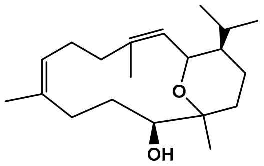	–7.6
215	Sarcophinediol	*S. glaucum*	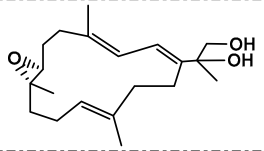	–8.6
216	7-keto-8α-hydroxy-deepoxysarcophine	*S. ehrenbergi*	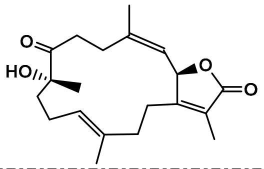	–7.8
217	7β-chloro-8α-hydroxy12acetoxy-deepoxysarcophine	*S. ehrenbergi*	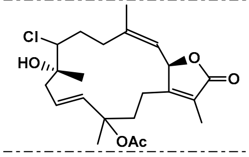	–7.4
218	Dihydrosarsolenone	*S. trocheliophorum*	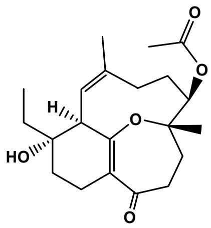	–8.1
219	Methyl dihydrosarsolenoneate	*S. solidum*	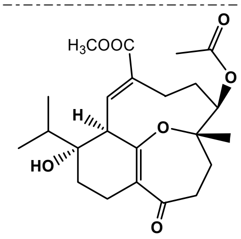	–8.1

220	Sarsolilide B	*S. trocheliophorum*	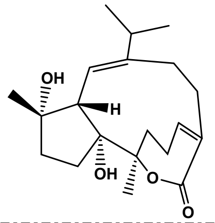	–7.4
221	Sarsolilide C	*S. trocheliophorum*	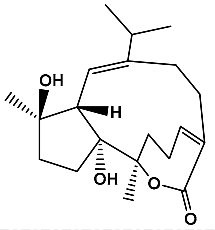	–7.1
222	Sarsolilide A	*S. solidum*	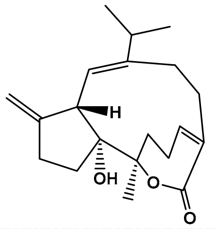	–7.4
223	Sarsolenone	*S. solidum*	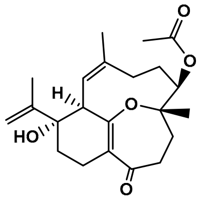	–8.9
224	Trocheliophol A	*S. trocheliophorum*	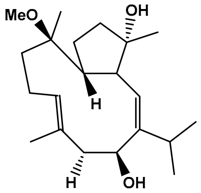	–7.3
225	Trocheliophol B	*S. trocheliophorum*	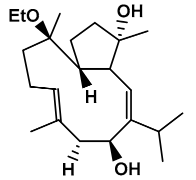	–7.4
226	Trocheliophols C	*S. trocheliophorum*	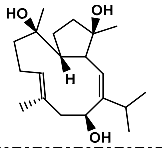	–6.8
227	Trocheliophol D	*S. trocheliophorum*	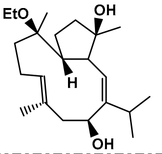	–6.8
228	Trocheliophol E	*S. trocheliophorum*	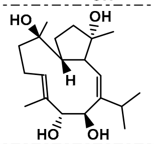	–6.9
229	Trocheliophol F	*S. trocheliophorum*	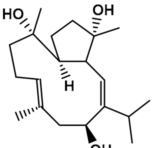	–7.4

230	Trocheliophol G	*S.*<br>*trocheliophoru*<br>*m*	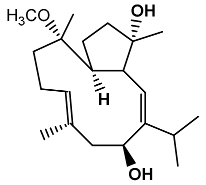	–7.3
231	Trocheliophol H	*S.*<br>*trocheliophoru*<br>*m*	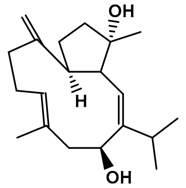	–7.3
232	Trocheliophol I	*S.*<br>*trocheliophoru*<br>*m*	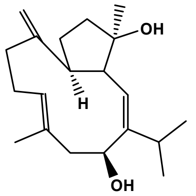	–6.9
233	Trocheliophol J	*S.*<br>*trocheliophoru*<br>*m*	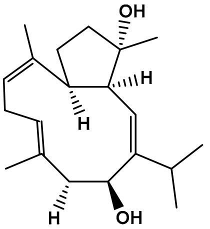	–7.1
234	Trocheliophol K	*S.*<br>*trocheliophoru*<br>*m*	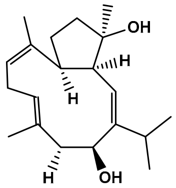	–7.7
235	Trocheliophol L	*S.*<br>*trocheliophoru*<br>*m*	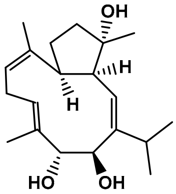	–7.6
236	Trocheliophol M	*S.*<br>*trocheliophoru*<br>*m*	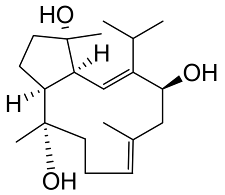	–6.6
237	Trocheliophol N	*S.*<br>*trocheliophoru*<br>*m*	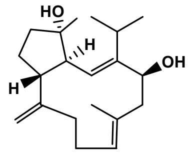	–6.9
238	Trocheliophol O	*S.*<br>*trocheliophoru*<br>*m*	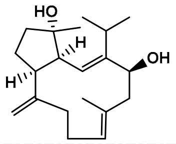	–6.9
239	Trocheliophol P	*S.*<br>*trocheliophoru*<br>*m*	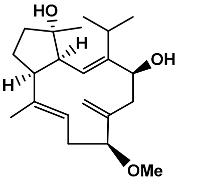	–7.6

240	Trocheliophol Q	*S. trocheliophorum*	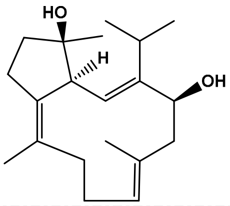	–6.9
241	Trocheliophol R	*S. trocheliophorum*	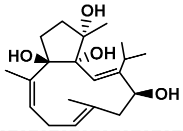	–6.9
242	Trocheliophol S	*S. trocheliophorum*	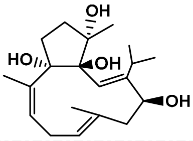	–6.1
243	4-epi-sarcophytol L	*S. elegans*	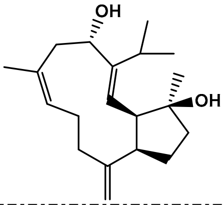	–8.0
244	Sarcophelegan A	*S. elegans*	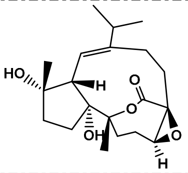	–7.4
245	Sarcophelegan B	*S. elegans*	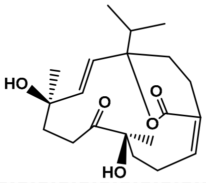	–7.5
246	Sarcophelegan C	*S. elegans*	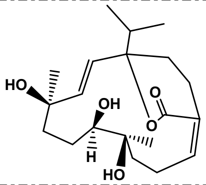	–7.8
247	Sarcophelegan D	*S. elegans*	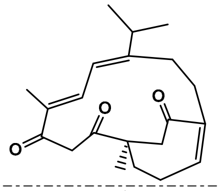	–7.9
248	(+)-1,15-epoxy-2-methoxy-12methoxycarbonyl-11E-sarcophytoxide	*S. ehrenbergi*	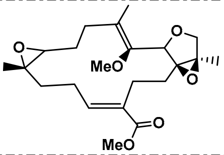	–8.4
249	(+)-2-epi-12-methoxycarbonyl-11E-sarcophine	*S. ehrenbergi*	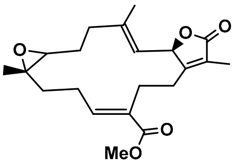	–7.4
250	3,4-epoxyehrenberoxide A	*S. ehrenbergi*	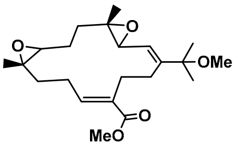	–6.9

251	Ehrenbergol D	*S. ehrenbergi*	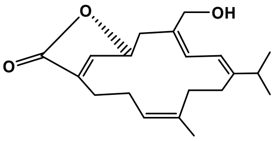	–6.7
252	Ehrenbergol E	*S. ehrenbergi*	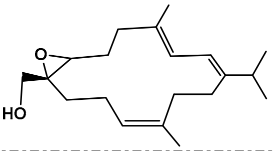	–7.7
253	7-deacetyl-sarsolenone	*S. solidum*	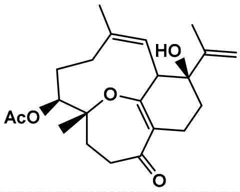	–8.0
254	Trocheliane	*S. trocheliophorum*	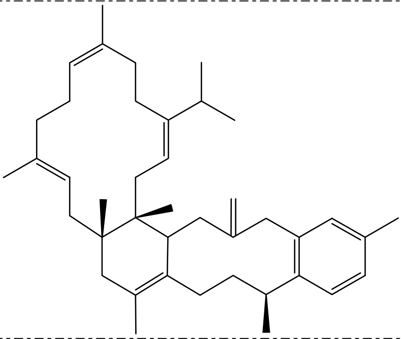	–6.9
255	Sarcotrocheldiol A	*S. trocheliophorum*	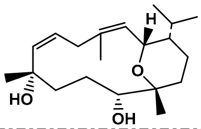	–8.2
256	Sarcotrocheldiol B	*S. trocheliophorum*	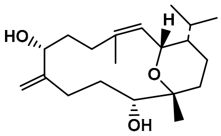	–6.8
257	16-hydroxycembra-1,3,7,11-tetraene	*Sarcophyton sp.*	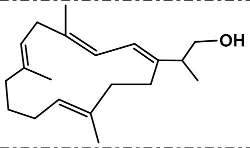	–7.8
258	15-hydroxycembra-1,3,7,11-tetraene	*Sarcophyton sp.*	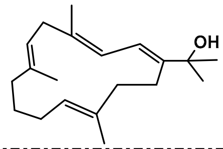	–8.0
259	Sarcophytrol D	*S. trocheliophorum*	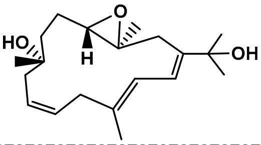	–7.4
260	Sarcophytrol E	*S. trocheliophorum*	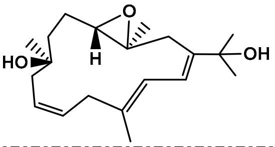	–7.9
261	Sarcophytrol F	*S. trocheliophorum*	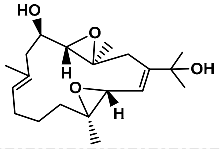	–7.2

262	11,12-epoxy-1(E),3(E), 7(E)-cembratrien-15-ol	*S. trocheliophorum*	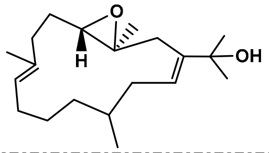	–7.9
263	Sinugibberol	*S. trocheliophorum*	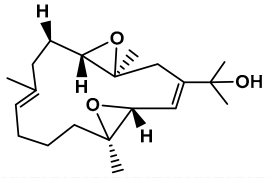	–7.7
264	Sarcophytrol G	*S. trocheliophorum*	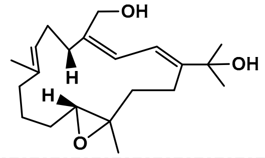	–8.3
265	Sarcophytrol H	*S. trocheliophorum*	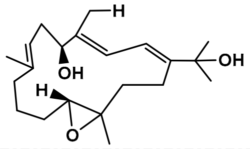	–7.8
266	Sarcophytrol I	*S. trocheliophorum*	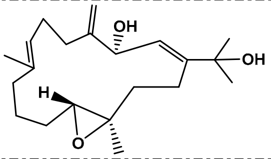	–7.8
267	Sarcophytrol J	*S. trocheliophorum*	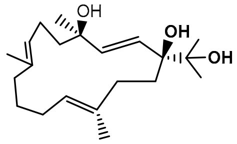	–6.1
268	Sarcophytrol K	*S. trocheliophorum*	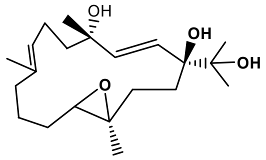	–6.6
269	Sarcophytrol L	*S. trocheliophorum*	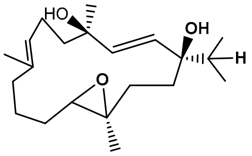	–6.4
270	Crassumol A	*S. trocheliophorum*	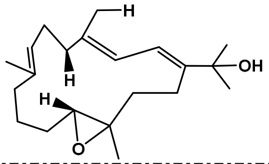	–8.0
271	Sarcophytrol M	*S. trocheliophorum*	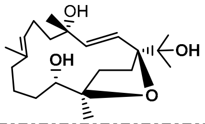	–7.0
272	Sarcophytrol N	*S. trocheliophorum*	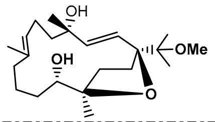	–7.0
273	Sarcostolide O	*S. trocheliophorum*	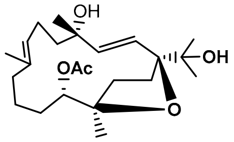	–7.6

274	Sarcophytrol P	*S. trocheliophorum*	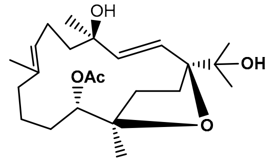	–6.9
275	Sarcophytrol Q	*S. trocheliophorum*	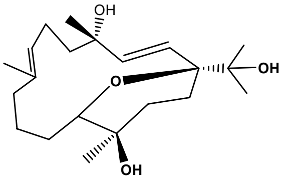	–7.5
276	Sarcophytrols R	*S. trocheliophorum*	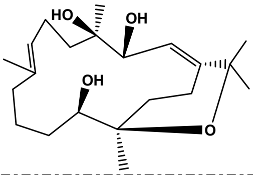	–7.4
277	Sarcophytrol S	*S. trocheliophorum*	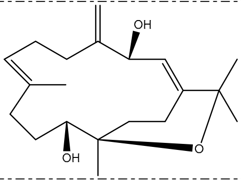	–6.5
278	Sarcophytrol T	*S. trocheliophorum*	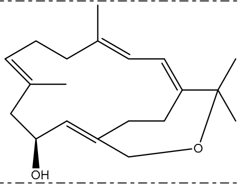	–7.0
279	Sarcophytrol U	*S. trocheliophorum*	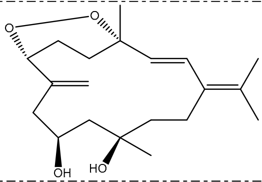	–8.0
280	Trocheliolide B	*S. trocheliophorum*	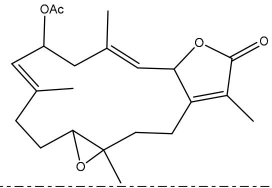	–7.0
281	9-Hydroxy-10,11-dehydro-sarcotrocheliol	*S. trocheliophorum*	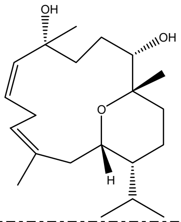	–7.7
282	Sarcophytonoxide A	*S. ehrenbergi*	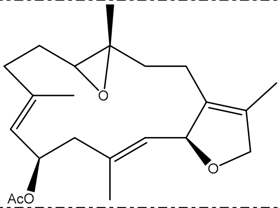	–7.3
283	Sarcophytonoxide B	*S. ehrenbergi*	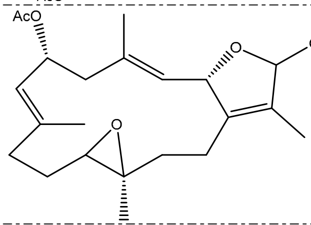	–7.9

284	Sarcophytonoxide C	*S. ehrenbergi*	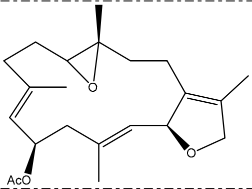	–6.9
285	Sarcophytonoxide D	*S. ehrenbergi*	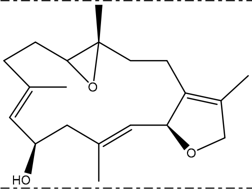	–6.5
286	Sarcophytonoxide E	*S. ehrenbergi*	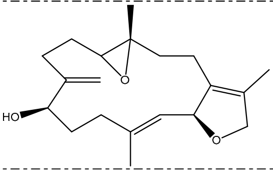	–8.0
287	Secodihydrosarsolenone	*S. trocheliophoru m*	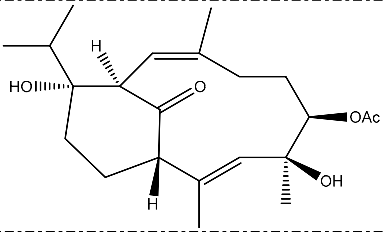	–8.1
288	(+)-(1E,3E,11E)-7,8-epoxycembra-1,3,11,15-tetraene	*S. stellatum*	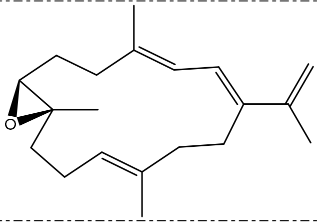	–7.8
289	(+)-(7R,8R,14S,1Z,3E,11E)-14-acetoxy-7,8-epoxycembra-1,3,11-Triene	*S. stellatum*	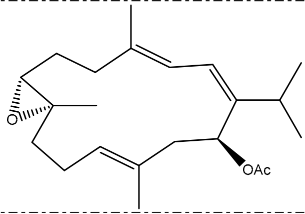	–7.6
290	3,4,8,16-tetra-epi-lobocrasol	*S. glaucum*	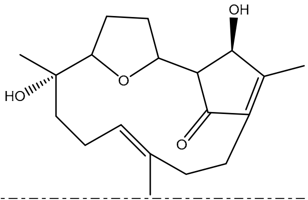	–7.1
291	1,15β-epoxy-deoxysarcophine	*S. glaucum*	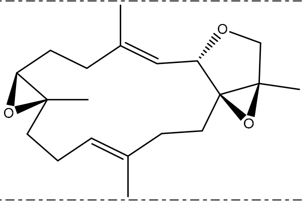	–7.3
292	3,4-dihydro-4α,7β,8α-trihydroxy-Δ^2-sarcophine	*S. glaucum*	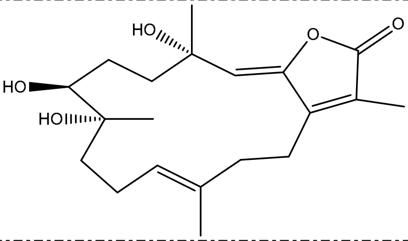	–8.6

293	*ent*-sarcophyolide E	*S. glaucum*	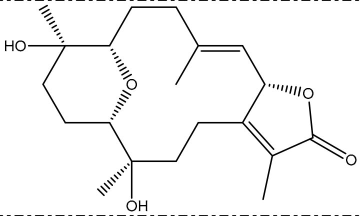	–7.8
294	3,4-dihydro-4α-hydroxy-Δ^2-sarcophine	*S. glaucum*	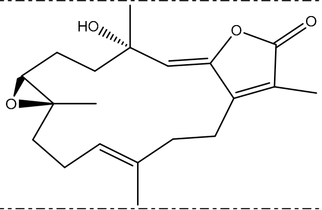	–7.3
295	3,4-dihydro-4β-hydroxy-Δ^2 -sarcophine	*S. glaucum*	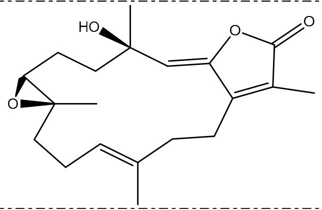	–7.7
296	Klyflaccicembranol F	*S. glaucum*	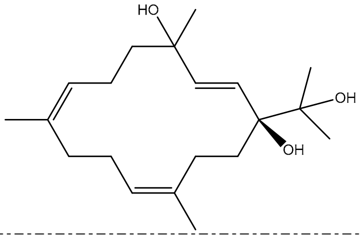	–7.6
297	Sarelengan C	*S. elegans*	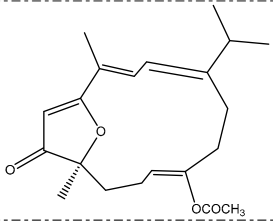	–8.2
298	Sarelengan D	*S. elegans*	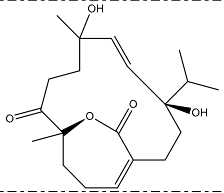	–6.7
299	Sarelengan E	*S. elegans*	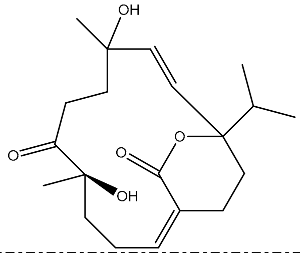	–8.4
300	Sarelengan F	*S. elegans*	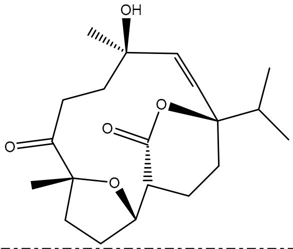	–7.8

301	Sarelengan G	*S. elegans*	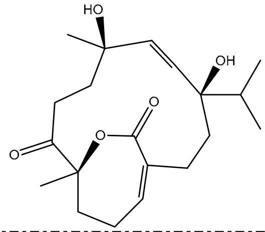	–7.4
302	Sarcoehrenbergilid A	*S. ehrenbergi*	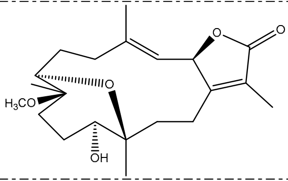	–6.9
303	Sarcoehrenbergilid B	*S. ehrenbergi*	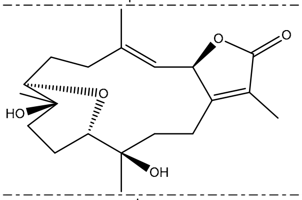	–8.8
304	Sarcoehrenbergilid C	*S. ehrenbergi*	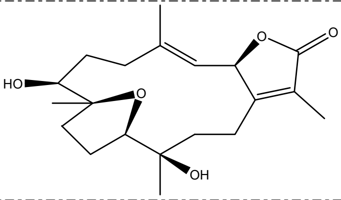	–8.2
305	Sinulolides A	*S. ehrenbergi*	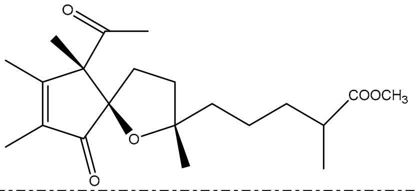	–7.4
306	Sinulolide B	*S. ehrenbergi*	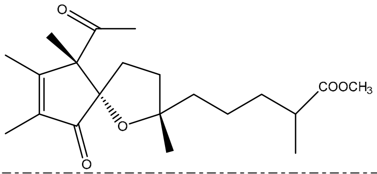	–7.3
307	Sarcoehrenbergilid D	*S. ehrenbergi*	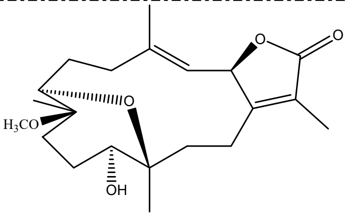	–7.2
308	Sarcoehrenbergilid E	*S. ehrenbergi*	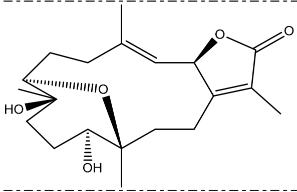	–7.0
309	Sarcoehrenbergilid F	*S. ehrenbergi*	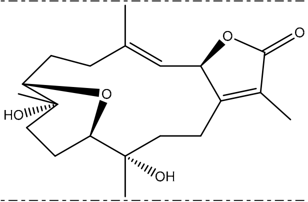	–7.8

310	Sarcoehrenolide A	*S. ehrenberg*	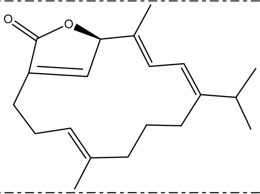	–7.7
311	Sarcoehrenolide B	*S. ehrenbergi*	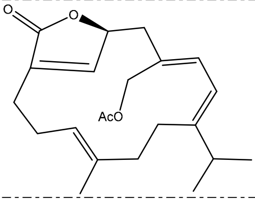	–7.0
312	Sarcoehrenolide C	*S. ehrenbergi*	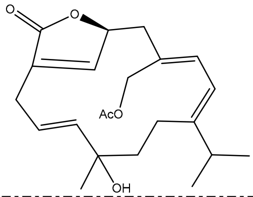	–6.8
313	Sarcoehrenolide D	*S. ehrenbergi*	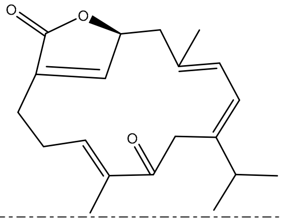	–7.6
314	Sarcoehrenolide E	*S. ehrenbergi*	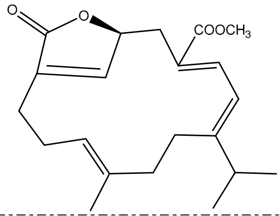	–7.5
315	Sarinfacetamide A	*S. infundibuliform e*	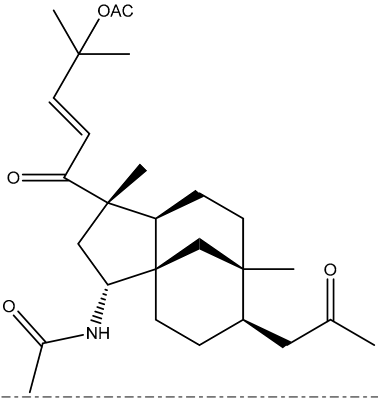	–7.4
316	Sarinfacetamides B	*S. infundibuliform e*	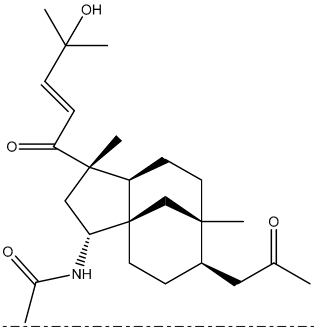	–8.4

317	Nanolobatin B	*S. infundibuliforme*	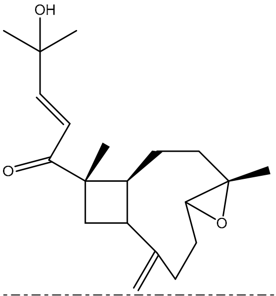	–8.1
318	(1S,2E,4R,6E,8S,11S,12S)-11,12-epoxy-8-hydroperoxy-4-hydroxy-2,6-cembradiene	*Sarcophyton sp.*	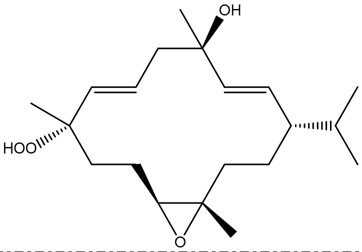	–8.6
319	Sarcomililatol A	*S. mililatensis*	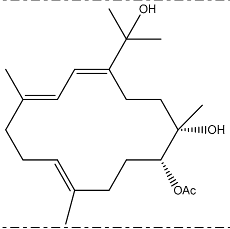	–7.2
320	Sarcomililatol B	*S. mililatensis*	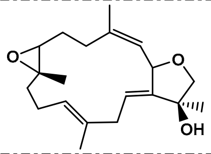	–7.8
321	Sarcomililate A	*S. mililatensis*	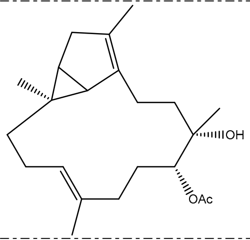	–5.9
322	Sarcophytol M	*S. mililatensis*	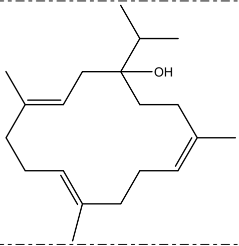	–7.7
323	9-hydroxy-7,8dehydro-sarcotrocheliol	*S. trocheliophorum*	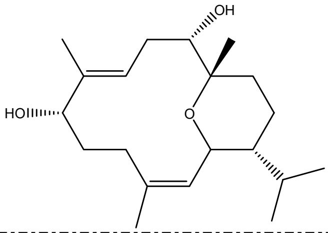	–6.6
324	8,9-expoy-sarcotrocheliol acetate	*S. trocheliophorum*	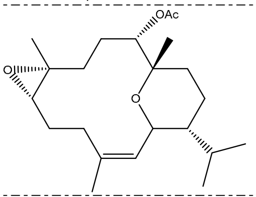	–6.9

325	Bisglaucumlide A	*S. glaucum*	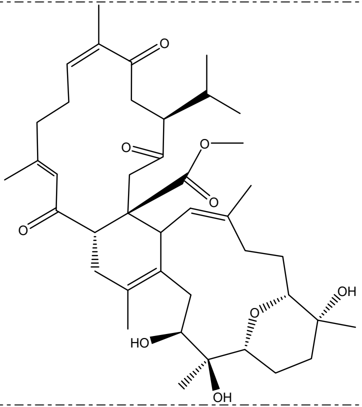	–9.6
326	Bisglaucumlide B	*S. glaucum*	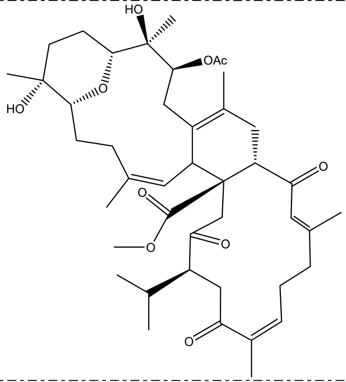	–8.1
327	Bisglaucumlide C	*S. glaucum*	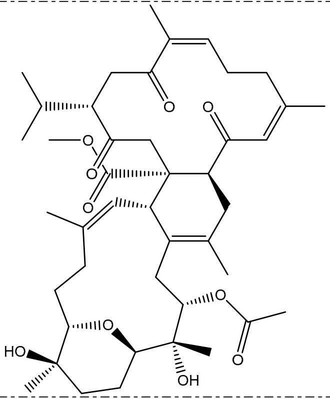	–5.1
328	Bisglaucumlide D	*S. glaucum*	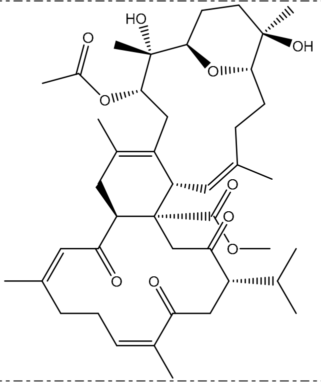	–5.7
329	Glaucumolide A	*S. glaucum*	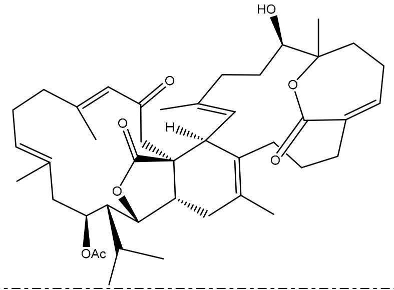	–8.0

330	Glaucumolide B	*S. glaucum*	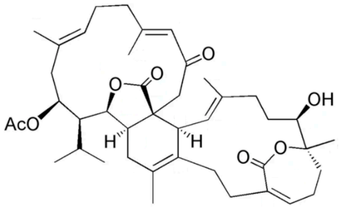	–7.3
331	Ximaolide A	*S. tortuosum*	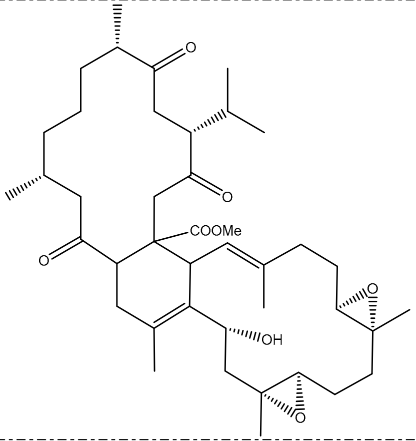	–9.3
332	Ximaolide B	*S. tortuosum*	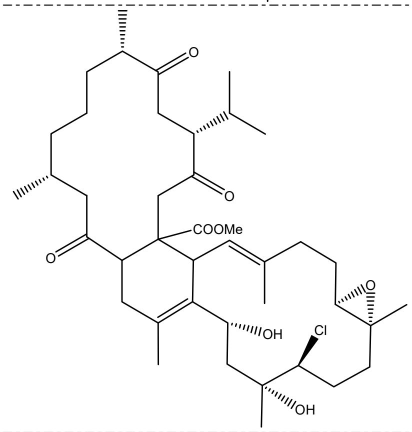	–8.2
333	Ximaolide C	*S. tortuosum*	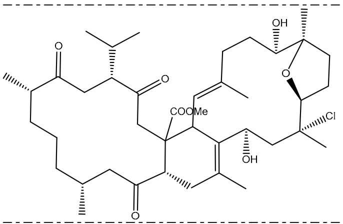	–7.6
334	Ximaolide D	*S. tortuosum*	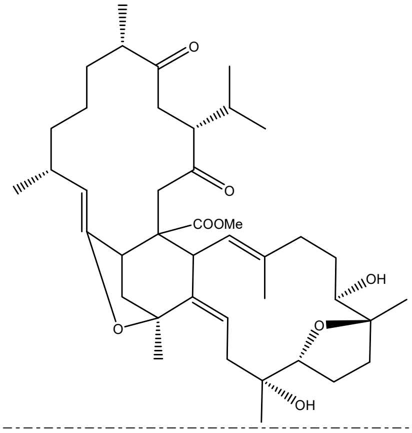	–8.0

335	Ximaolide E	*S. tortuosum*	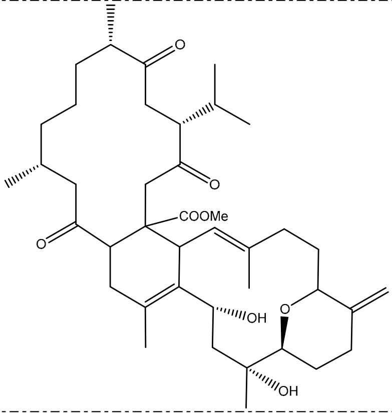	–9.0
336	Ximaolide F	*S. tortuosum*	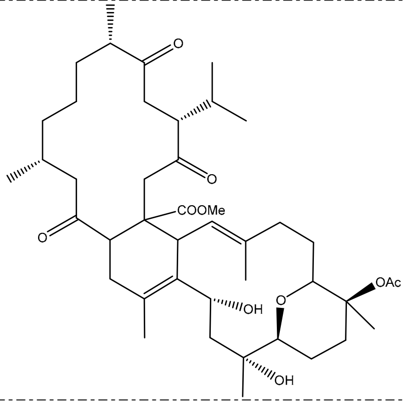	–9.5
337	Ximaolide G	*S. tortuosum*	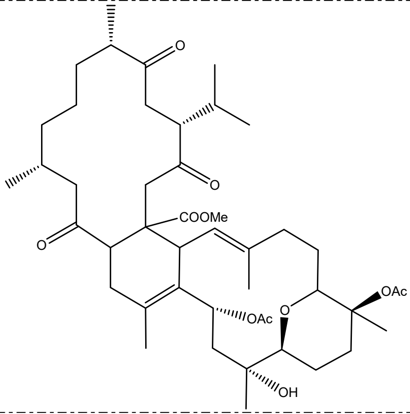	–7.3
338	Methyl tortuosoate A	*S. tortuosum*	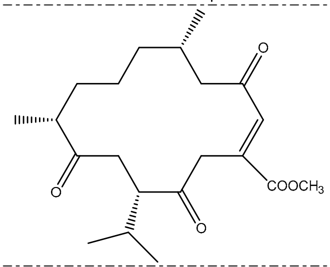	–8.4
339	Isosarcophytonolide D	*S. tortuosum*	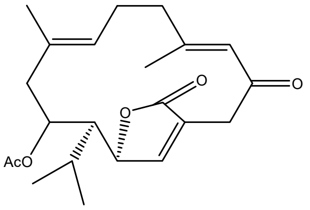	–7.6

340	Bislatumlide A	*S. latum*	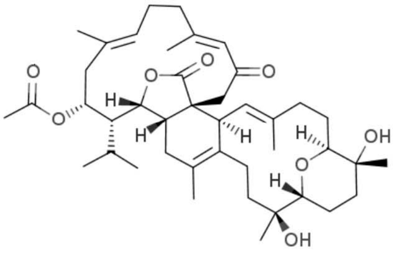	–9.6
341	Bislatumlide B	*S. latum*	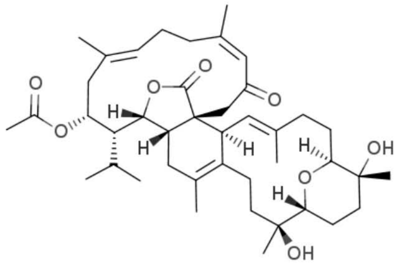	–8.2
342	Methyl tetrahydrosarcoate	*S. elegans*	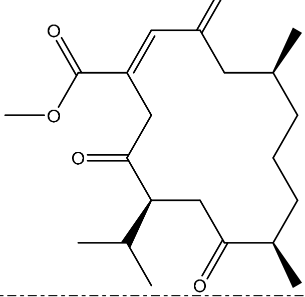	–8.1
343	Methyl tetrahydroisosarcoate	*S. elegans*	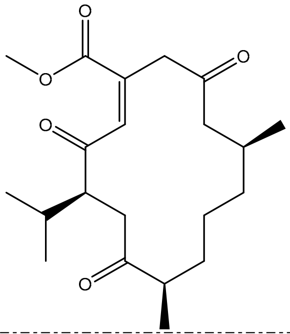	–7.4
344	Nyalolide	*S. elegans*	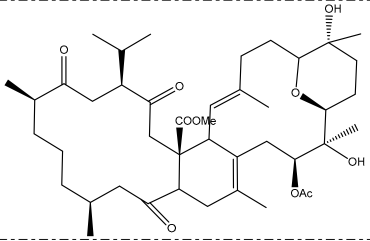	–7.5
345	Desacetylnyalolide	*S. elegans*	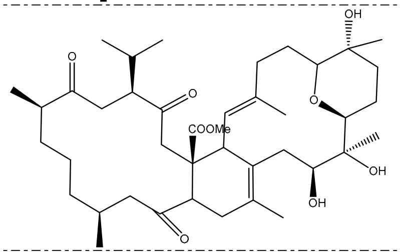	–8.3
346	Diepoxynyalolide	*S. elegans*	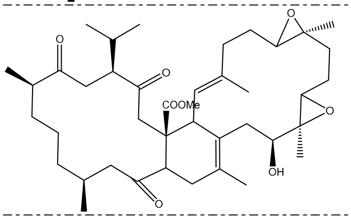	–7.7

347	Dioxanyalolide	*S. elegans*	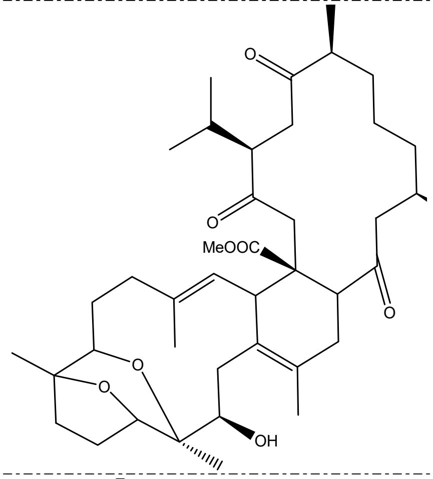	–9.5
348	Sarcophytolide G	*S. elegans*	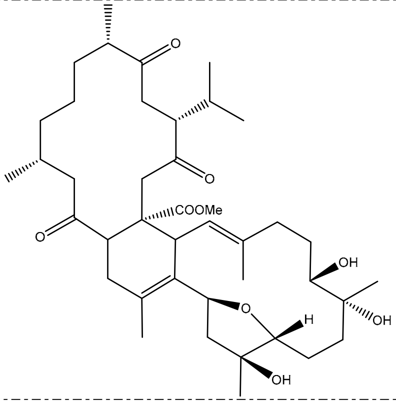	–7.4
349	Sarcophytolide H	*S. glaucum*	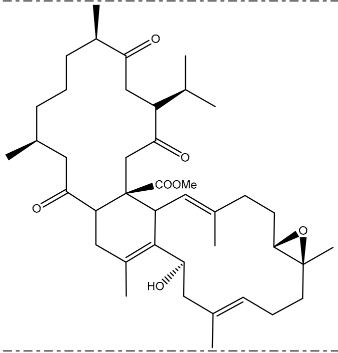	–8.5
350	Sarcophytolide I	*S. elegans*	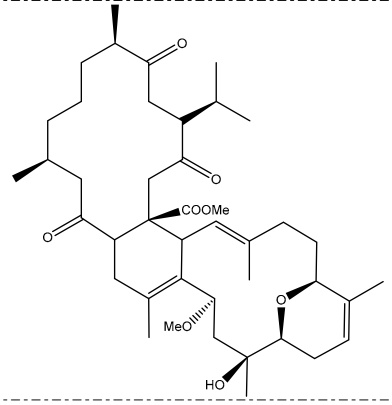	–7.5

351	Sarcophytolide J	*S. elegans*	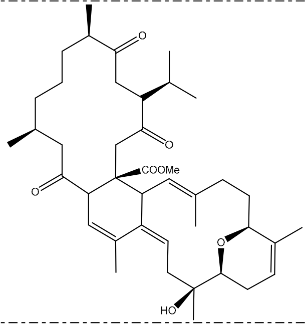	–8.0
352	Sarcophytolide K	*S. elegans*	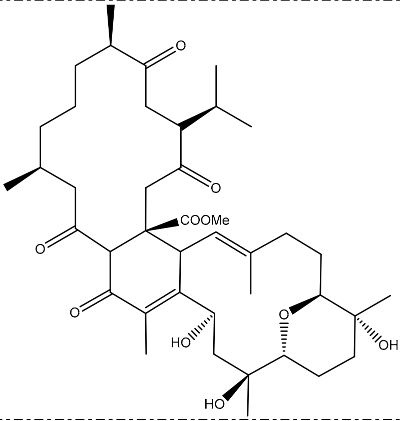	–7.1
353	Sarcophytolide L	*S. elegans*	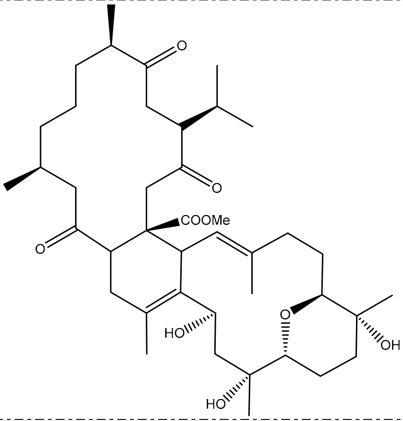	–6.9
354	Lobophytone H	*S. elegans*	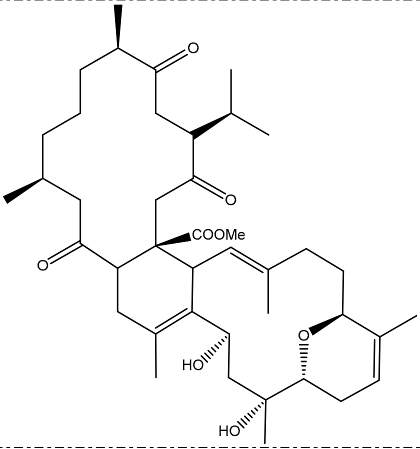	–8.1

355	Lobophytone I	*S. elegans*	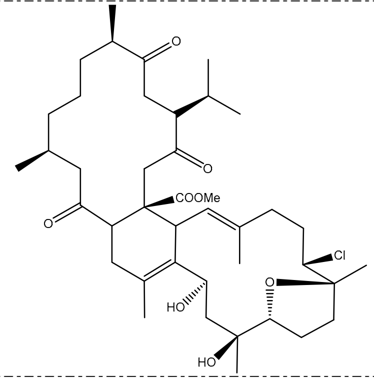	–8.5
356	Lobophytone K	*S. elegans*	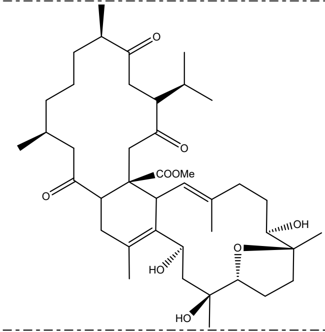	–8.3
357	Lobophytone W	*S. elegans*	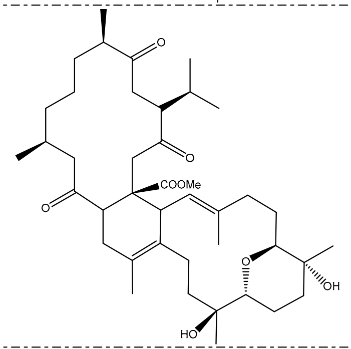	–8.7
358	Lobophytone U	*S. pauciplicatum*	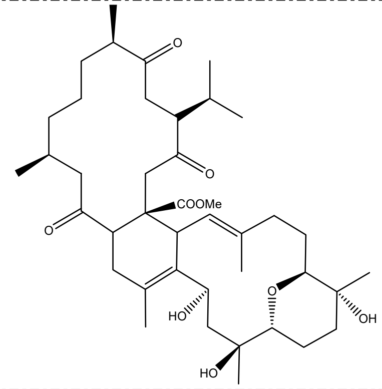	–8.6
359	Sarcophytolide M	*S. pauciplicatum*	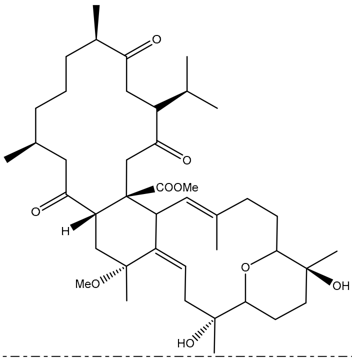	–7.2

360	Sarcophytolide N	*S. pauciplicatum*	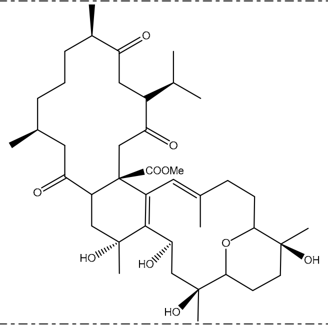	–7.0
361	Lobophytone O	*S. pauciplicatum*	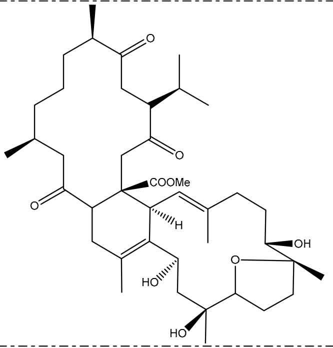	–8.6
362	Sarelengan A	*S. elegans*	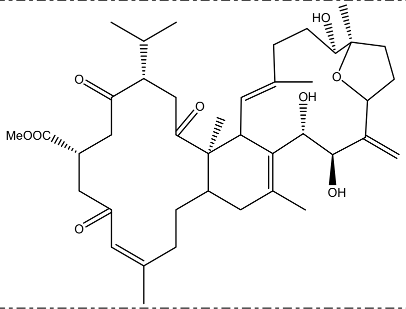	–8.1
363	Sarelengan B	*S. elegans*	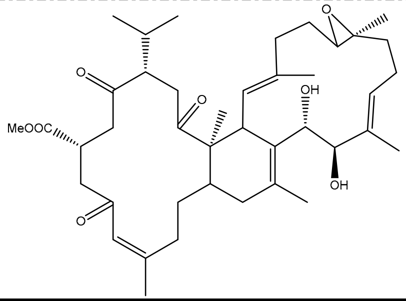	–9.8

[a]Data sorted according to the order of the compounds in Ref. (Elkhawas et al., 2020).

Table S2. Computed Autodock and MM/GBSA binding energies (in kcal/mol) for darunavir and the top 58 potent *Sarcophyton* cembranoid diterpenes metabolites against SARS-CoV-2 main protease (M^{pro}) over 250 ps implicit solvent MD simulations[a].

Compound Name	Docking Score (kcal/mol)	MM/GBSA Binding Energy (kcal/mol)
Darunavir	–8.2	–31.1
Bislatumlide A, B (**340**)	–9.6	–45.1
Desacetylnyalolide (**345**)	–8.3	–43.3
Dioxanyalolide (**347**)	–9.5	–40.8
Sarelengan B (**363**)	–9.8	–40.7
Lobophytone W (**357**)	–8.7	–39.2
Bisglaucumlide A (**325**)	–9.6	–31.0
Sarinfacetamides B (**316**)	–8.4	–31.0
Methyl sarcotroate B (**173**)	–8.5	–30.9
Sarcophytonolide B (**39**)	–8.7	–30.8
Sarcophytolide H (**349**)	–8.5	–30.8
Sarcocrassocolide E (**141**)	–8.3	–30.7
Sarcocrassocolide N (**151**)	–9.4	–30.7
Ximaolide A (**331**)	–9.3	–30.6
7-Acetyl-8-epi- sinumaximol G (**131**)	–8.3	–30.6
Ximaolide E (**335**)	–9.0	–30.5
Methyl tetrahydrosarcoate and methyl tetrahydroisosarcoate (**342**)	–8.3	–30.5
Sarcophinediol (**215**)	–8.6	–30.5
Lobophytone K (**356**)	–8.3	–30.5
Sarcocrassocolide H (**145**)	–8.3	–30.2
Sarcocrassocolide J (**147**)	–9.1	–30.1
Sarcocrassocolide I (**146**)	–8.3	–30.1
Lobophytone O (**361**)	–8.6	–30.0
Sarcofuranocembrenolide A (**127**)	–8.5	–29.9
Ent-sarcophine (**122**)	–8.6	–29.8
Lobophytone U (**358**)	–8.6	–29.6
Lobophytone I (**355**)	–8.5	–29.4
Tortuosene A (**201**)	–8.5	–29.4
Sarcophyolide A (**187**)	–9.3	–29.3
(1R,2E,4S,6E,8R,11R,12R)-2,6-cembradiene-4,8,11,12-tetrol (**204**)	–8.3	–29.2
Sarcophytrol G (**264**)	–8.3	–29.0
(+)-12-methoxycarbonyl-11Z-sarcophine (**158**)	–8.9	–28.8
(+)-1,15-epoxy-2-methoxy-12methoxycarbonyl-11E-sarcophytoxide (**248**)	–8.4	–28.5
Ximaolide F (**336**)	–9.5	–28.5
Sarcocrassocolide F (**143**)	–9.0	–28.5
Sarcophytonolide M (**174**)	–9.0	–28.3
3,4-dihydro-4α,7β,8α-trihydroxy-Δ^2-sarcophine (**292**)	–8.6	–28.1
Glaucumolide A (**329**)	–9.4	–27.9
Sarelengan E (**299**)	–8.4	–27.4
Sarcocrassolide A (**8**)	–9.0	–27.2
Methyl tortuosoate A (**338**)	–8.4	–27.2
(4Z,12Z,14E)-sarcophytolide (**31**)	–8.8	–26.2

Sarcoehrenbergilid B (**303**)	–8.8	–26.2
Ehrenbergol B (**180**)	–8.8	–26.0
Sarcocrassocolide G (**144**)	–8.5	–25.9
Sarsolenone (**223**)	–8.9	–25.4
Crassolide (**7**)	–8.5	–25.3
Sarcophytonins F (**129**)	–8.3	–25.2
Lobophynin C (**162**)	–8.5	–25.2
(1S,2E,4R,6E,8S,11S,12S)-11,12-Epoxy-2,6-cembrane-4,8-diol (**17**)	–8.3	–24.9
Secosarcophinolide (**121**)	–8.4	–24.8
Sacostolide D (**105**)	–8.4	–24.3
Acetyl ehrenberoxide B (**194**)	–8.4	–24.2
Sarcophytonolide C (**40**)	–8.4	–23.6
Sarcocrassocolide B (**138**)	–8.6	–23.6
Denticulatolide (**10**)	–9.9	–23.3
(1S,2E,4R,6E,8S,11S,12S)-11,12-epoxy-8-hydroperoxy-4-hydroxy-2,6-cembradiene (**318**)	–8.6	–22.8
11(S)-hydroperoxylsarcoph-12(20)-ene (**169**)	–8.3	–22.3
Sarcocrassocolide A (**137**)	–8.3	–21.1
Sarcocrassocolide K (**148**)	–8.3	–20.0

[a]Data sorted according to the calculated MM/GBSA binding energies.

Table S3. Network topological analysis for the predicted targets for bislatumlide A (**340**).

Target	Betweenness Centrality	Closeness Centrality	Degree
SRC	0.140277931	0.674603175	46
MAPK1	0.097973132	0.625000000	37
AR	0.076882543	0.586206897	32
MAPK8	0.043685125	0.582191781	29
MDM2	0.05093431	0.548387097	27
ERBB2	0.060499285	0.551948052	26
PTGS2	0.062644806	0.562913907	24
IGF1R	0.019882126	0.534591195	22
HSP90AB1	0.031359945	0.521472393	22
MMP2	0.045495406	0.53125000	21
GSK3B	0.020712726	0.537974684	21
PRKCA	0.031388573	0.527950311	21
PLCG1	0.015506616	0.515151515	21
CDKN1B	0.014823023	0.518292683	20
LCK	0.026151452	0.518292683	19
RPS6KB1	0.01034058	0.515151515	19
CASR	0.043481068	0.459459459	18
MAPK14	0.013888034	0.512048193	18
NR3C1	0.034134996	0.534591195	18
CDK4	0.015939274	0.505952381	18

Table S4. Interactors list showing the top 10 genes stimulated by bislatumlide A (**340**), their UniProt Ids and list of genes Ids which Interacts with SARS-CoV-2.

Input	UniProt Id	Interacts with
**APP**	P05067-4, P05067, P05067-PRO_0000000093, P05067-PRO_0000000092,	P21359, P29353, P02649, P04629, P78352, P00533, P49841, 28815, P08138, Q96FE5, P01100
**JAK2**	O60674, Q62120	P46527, O60674, P18031
**AR**	P10275	P24385, Q92793, Q92993, O14686, P35222, O75593, P55317, Q15596, Q02790, Q9NSA3, Q9UBL3
**JUN**	P05412	P05412, O95644, P07900, Q9UPN9, P15336, P18846, Q00987, P16220, P17275, P52292, P45983, Q15796, Q99966, P01100
**LYN**	P07948-1, P07948	P46527, Q07666, Q9NWQ8, P12931, P00533, Q05397
**MAPK1**	P28482	P35813, P10415, Q9BUB5, P28482, P27361, Q15121, P35236, P32121, P46531, Q02750, Q12913, P29353-2
**MAPK3**	P27361	Q14160, P19419, P28482, P27361, Q15121, Q02750, Q12913
**MAPK3**	P27361	Q14160, P19419, P28482, P27361, Q15121, Q02750, Q12913
**MAPK8**	P45983	P05412, Q07817, P00533, P27986, P46108, P60953
**MDM2**	Q00987, EBI-2694074	P05412, P49674, Q13547, Q15648, P31749, P32121, P29590, P48729, Q00987, P04271, P08047, Q9Y297, P78352, Q93009, P49757
**SRC**	P05480, P00523, P12931, Q9WUD9	P35968, P07900, Q68CZ2, P22681, P18031, Q07666, P32121, P35222, P56945, Q05397, P00519, P08581, Q9Y4D1, P41240, P06241, P07550, P00533, 15532, P27986, Q14289, Q13177, P40763
